# Chemical Structure Controls Soil Biotransformation Kinetics of Bacillus Lipopeptide Biopesticides

**DOI:** 10.1021/acs.est.6c06707

**Published:** 2026-09-25

**Authors:** Richard Gruseck, Thilo Hofmann, Michael Zumstein

**Affiliations:** † Department of Environmental Geosciences, Centre for Microbiology and Environmental Systems Science, 27258 University of Vienna, Vienna 1090, Austria; ‡ Doctoral School in Microbiology and Environmental Science, University of Vienna, Vienna 1030, Austria

**Keywords:** biodegradation, environmental benign by design, sustainable agriculture, HRMS-based transformation product analysis, surfactin, iturin, fengycin

## Abstract

*Bacillus* lipopeptides (surfactins, iturins, and fengycins) are an emerging class of biopesticides. However, their fate in agricultural soils, and specifically how variations in the chemical structure such as alkyl chain length, amino acid sequence, and cyclization affect biotransformation, remains poorly understood. Here, we assessed biotransformation kinetics and pathways of a representative set of lipopeptides by coupling laboratory soil incubation experiments with HPLC-HRMS analyses. Half-lives varied strongly across lipopeptide families, with fastest degradation for iturins (1–8 d), followed by fengycins (5–15 d) and surfactins (6–67 d). In one of the three tested soils, surfactins exhibited biphasic removal with a substantial fraction (i.e., ∼21% of the spiked amount) remaining after >100 days of incubation, indicating formation of a poorly bioavailable fraction. Additionally, our results revealed a strong effect of the alkyl chain length for certain lipopeptides, with a half-life increase per additional CH_2_ unit up to ∼45% for iturins and ∼110% for surfactins. Lastly, we report that linearized surfactins were transformed substantially faster than cyclic surfactins. Together, the reported links between chemical structure and soil biotransformation kinetics can inform the selection, and possibly also the (re)­design, of lipopeptide biopesticides for improved biodegradability in agricultural soils.

## Graphical abstract

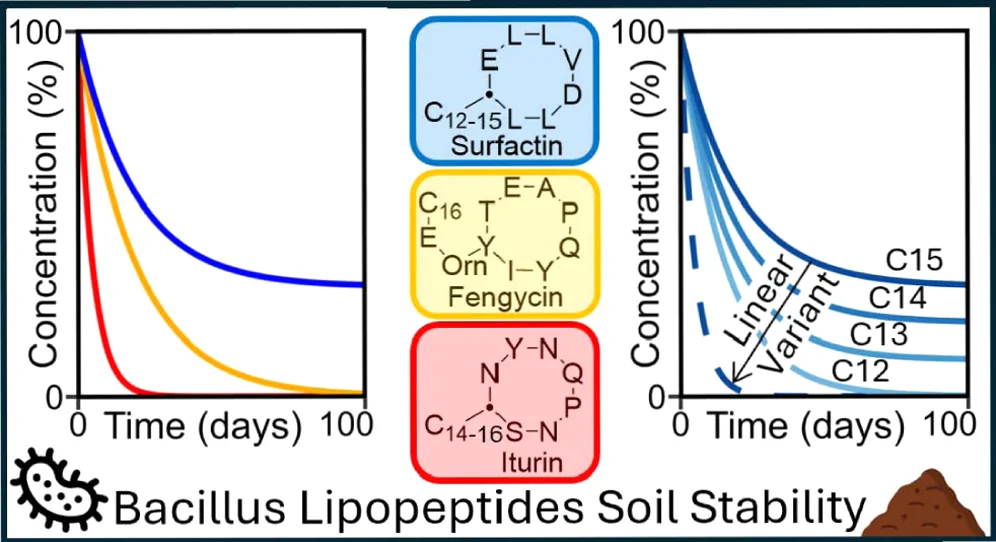

## Introduction

There is a great demand for chemicals that are effective during use yet do not persist in the environment.^1,2^ To match this demand, we need to understand the key factors that control the stability of a chemical in the respective compartment, particularly for chemicals that are intentionally employed in an environmental compartment, such as pesticides applied to agricultural fields.^3,4^ In this context, biomolecules are increasingly proposed as alternatives for xenobiotic chemicals, with biopesticides and biosurfactants being prominent examples.^5–8^ One class of biomolecules that are increasingly discussed for agricultural applications is lipopeptides from *Bacillus* spp., which can provide a range of biological activities (e.g., antifungal or antibacterial), surface activity, and emulsification capacity.^5,9,10^

Lipopeptides from *B*. spp. (hereafter, lipopeptides) are categorized into three families: surfactins, fengycins, and iturins.^5,11^ Although often combined under the term lipopeptides, these three families vary in their chemical structures and physicochemical properties – including charge, cyclization pattern, and surfactant activity (Figure 1).^12^ Surfactins are cyclic heptapeptides containing an alkyl chain that is anchored in the cycle through an ester bond. They are among the most potent biosurfactants.^13,14^ Iturins are also cyclic heptapeptides, but their alkyl chain is anchored in the cycle through an amide bond. Fengycins are decapeptides with eight amino acid residues linked into a cycle through an ester bond, and with the alkyl chain being linked to two amino acid residues that are not part of the cycle. Members within these three families are further distinguished by their peptide sequence and by differing lengths (typically between C13 and C17) and branching patterns of the alkyl chain. Because *Bacillus* strains commonly coproduce several members, lipopeptide applications are mostly based on lipopeptide mixtures rather than on individual purified compounds.^15–17^ These structural differences are expected to influence both sorption behavior and susceptibility to enzymatic transformation.

**Figure 1:**
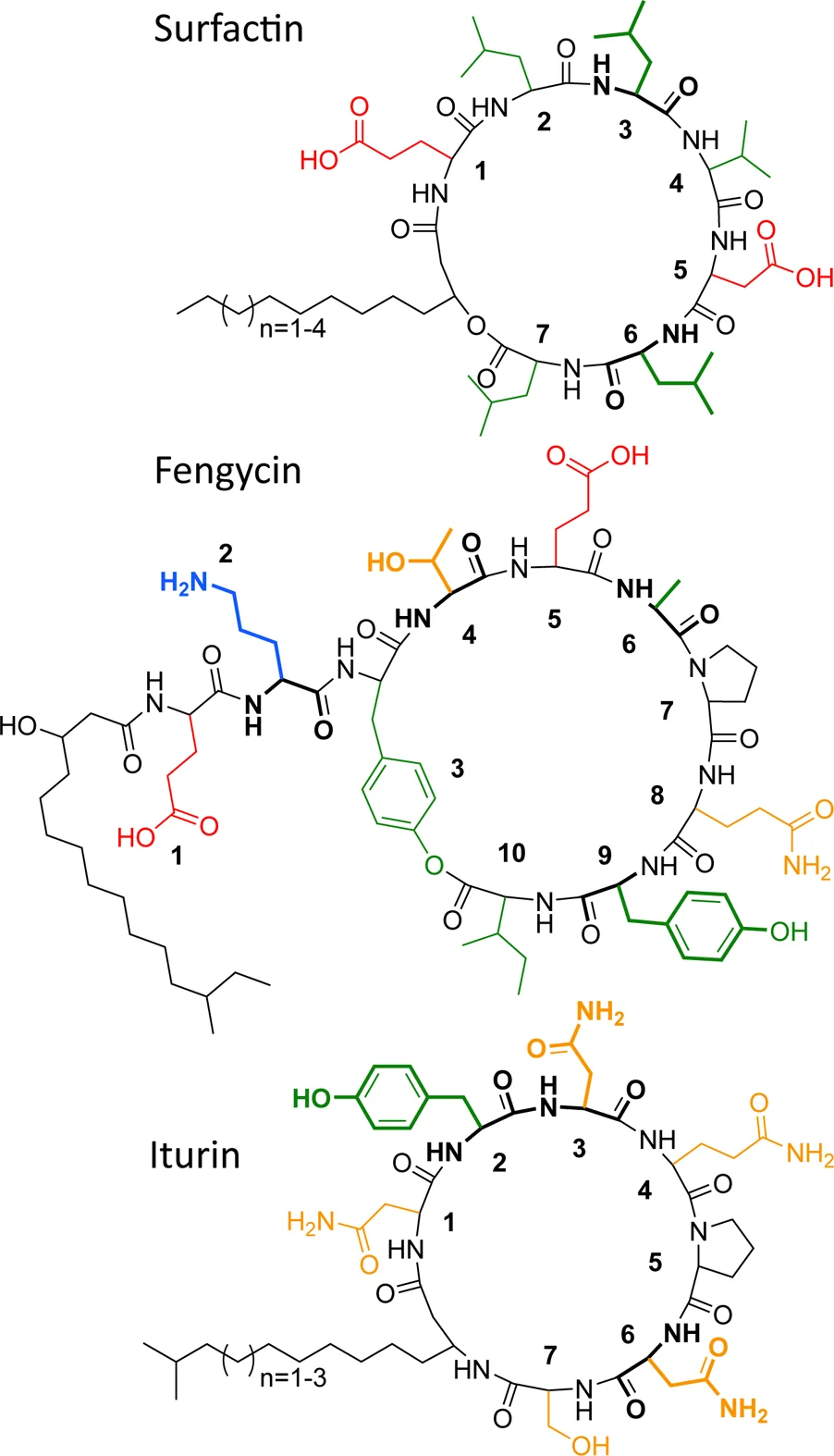
Structures of investigated lipopeptide families with numbered amino acid residues. Colored side chains are hydrophobic (green), polar (orange), or pH-dependent positively (blue) or negatively (red) charged. d-amino acid residues are depicted in bold. We note that while one of the three possible alkyl chain branching patterns is shown per family, each pattern can occur in all three families.

The potential of lipopeptides in agriculture has been emphasized in several recent publications.^5,16,18^ For example, a field study reported that a mixture of fengycins and iturins was successful in protecting banana plantations against black sigatoka, suggesting that lipopeptides are a promising alternative to fungicides such as chlorothalonil and mancozeb.^16^ These two fungicides have recently been banned in the European Union due to toxicity and groundwater pollution concerns, but remain widely used globally.^19,20^ Beyond scientific studies, a lipopeptide-producing *Bacillus* strain has also already been commercially marketed as a broad-spectrum biofungicide for foliar and soil diseases in three countries (i.e., Ivory Coast, Kenya, and Morocco), and a mixture of metabolites from *Bacillus subtilis*, most likely containing lipopeptides, has been marketed as a biostimulant in the European Union.^21,22^ In addition to these agricultural applications, lipopeptides have also been investigated to substitute synthetic surfactants for soil remediation.^23–25^ For example, one study showed that mixtures containing all three lipopeptide families enhanced the bioavailability of 4-, 5-, and 6-ring polycyclic aromatic hydrocarbons (including fluoranthene, chrysene, benzo­[*a*]­pyrene, and others) and thereby accelerated soil remediation from these contaminants.^25^ Similarly, it was shown that lipopeptides can potentially replace synthetic surfactants for remediation of oil-contaminated soils.^26–28^ These applications imply direct release of lipopeptides into environmental compartments, particularly soils, where their stability determines both efficacy and environmental risk.

While the above-mentioned developments are promising, it is important to emphasize that lipopeptides, and biomolecules in general, should not be considered rapidly biodegradable solely based on their natural origin and that a thorough understanding of their environmental stability is needed for an informed environmental risk assessment. Environmental stability is determined by an interplay between the chemical structure of a compound and the specific environmental conditions (e.g., presence and activity of enzymes with a matching substrate specificity).^29–31^ In soils, biotransformation is expected to be controlled not only by enzymatic processes but also by bioavailability limitations arising from sorption to soil constituents.^32^ For lipopeptides, several structural features, such as cyclization and nonproteogenic amino acid residues, were described to enhance proteolytic stability.^33,34^ Surfactins are expected to adsorb strongly onto soil components, as the increased conformational rigidity resulting from cyclization leads to a physical separation of charged and hydrophobic amino acid residues, thereby creating a hydrophobic side together with the alkyl chain.^35–37^ Based on the aforementioned chemical properties, it is possible that some lipopeptides exhibit a similar soil stability compared to xenobiotic pesticides. In line with a precautionary approach, soil persistence of lipopeptides can be viewed as a cause for concern on its own.^38,39^ However, the fate and stability of lipopeptides in soil have not been thoroughly investigated. The only study that, to our knowledge, examined the biodegradability of lipopeptides in soil is based on a sterile soil that was inoculated with specialized microorganisms capable of degrading petroleum hydrocarbons.^40^ Lipopeptides, including several surfactins, were then incubated in these soils, and respirometric analysis revealed that lipopeptide mineralization reached up to 70% within 1 week. Beyond this soil-focused study, lipopeptide (specifically surfactins) biodegradation has only been assessed in activated sludge.^41,42^ Although surfactin was biodegraded in activated sludge during a few days, up to 50% of surfactin was adsorbed to the activated sludge, and the fate of the adsorbed part was not further assessed. In summary, these studies suggest that some surfactins can biodegrade under certain conditions; however, there is no generalizable evidence for the complete biodegradability of lipopeptides and no insights into how biotransformation rates and kinetics are affected by the numerous varied structural features of lipopeptides.

The goal of this study was to address this knowledge gap by assessing structure-dependent biotransformation kinetics and by elucidating biotransformation pathways of a set of relevant lipopeptides in representative agricultural soils. Therefore, we selected (i) the name-giving lipopeptide of each family (surfactins, fengycins, and iturins) and (ii) a chemically linearized surfactin and performed laboratory incubation experiments with three standardized agricultural soils for >100 days. Lipopeptides were extracted at defined time points during these incubations and quantified through HPLC-HRMS analyses. For elucidating biotransformation pathways, we detected transformation products based on a combination of suspect screening and nontarget analysis.

## Methods

### Chemicals and Materials

All solutions were prepared with ultrapure water (ELGA PURELAB Chorus, 0.055 μS). Iturins (from *Bacillus subtilis*, ≥95%), surfactins (from *Bacillus subtilis*, ≥98%), hydrogen chloride (HCl, 1 M, Tritripur), sodium hydroxide (NaOH, ≥99.0%), potassium phosphate monobasic (≥99.0%), and potassium phosphate dibasic (≥98.0%) were purchased from Sigma-Aldrich. Fengycin (>90%) was purchased from Biosynth. Acetonitrile (ACN, LC–MS grade, ≥99.9%), methanol (MeOH, LC–MS grade, ≥99.9%), acetone (Extra Pure), and formic acid (LC/MS grade, ≥99.0%) were purchased from Fisher. Methanol (hypergrade for LC–MS, Merck) and 15 and 50 mL centrifuge tubes (metal-free) were purchased from VWR. 1.5 and 2 mL centrifuge tubes (protein LoBind tubes) were purchased from Eppendorf. A mixed stock solution of cyclic surfactins, fengycin, and iturins (each family 1 mg/mL) was prepared in 70% acetone (aq.) and stored at −18 °C. Calibration series were prepared from this stock solution in 35/35/30% methanol/acetonitrile/water covering a concentration range of 5–2000 μg/L.

### Linearization of Surfactins

For linearization of surfactins, we adapted a procedure from Morikawa et al.^43^ A solution of cyclic surfactins (5 mg) and NaOH (60 mM) in 40% aqueous methanol (1 mL) was horizontally shaken in a 2 mL centrifuge tube at 37 °C and at 750 rpm. After 17 h, the solution was acidified with HCl (1 M) to a pH of 4 and centrifuged (20,800*g* for 2 min). The precipitate was resolubilized in 40% ACN (0.5 mL) and neutralized to pH 7 by adding aqueous NaOH (0.1 M). After centrifugation (20,800*g* for 2 min), the clear solution was transferred to an HPLC vial. The crude product was further purified by preparative LC (column: ReproSphere 100 C18, 5 μm, 250 × 10 mm), and multiple fractions were collected individually. After LC–MS analyses, the fractions containing substantial amounts of linear surfactins were combined, dried under reduced pressure, and freeze-dried to obtain a mix of linear surfactins as a white powder (3.2 mg, yield: ∼60%). Linearization of surfactins yielded a mixture of homologues with a similar relative composition to the cyclic stock solution, except for 7-valine variants, which were not detected (Figures S1 and S[Fig fig2]). A stock solution of linearized surfactins (1 mg/mL) was prepared with 70% acetone (aq.) and buffered with PO_4_ (5.33 mM) at pH 7.

### Soil Incubation Experiments

The experimental protocol for soil incubation experiments and lipopeptide extraction was adapted from a previous study.^44^ Three soils from the “Landwirtschaftliche Untersuchungs und Forschungsanstalt (LUFA) Speyer” were selected [i.e., Soils 2.2, 5M, and 6 S, which vary in their soil textures (sand content: 23% to 74%) and pH (5.6 to 7.5)] (Table S1). The maximum water holding capacity ranged from 41 to 49 g/100 g soil, and the soil organic carbon content ranged from 1.2 to 1.8% carbon. These LUFA agricultural soils were stored at 4 °C upon shipment. Prior to the start of the experiment, the soil was prewetted to 50% of its maximum water holding capacity and incubated for 3 days at room temperature. For abiotic control incubations, soil was autoclaved 3 times on consecutive days at 121 °C for 30 min. We confirmed that autoclaving did not substantially affect the adsorption of lipopeptides: recoveries in autoclaved soils were 95% ± 17%, 97% ± 9%, and 92% ± 7% relative to those in nonautoclaved soils for Soil 2.2, 5 M, and 6 S, respectively. Soil incubations were performed in triplicate with 5 g soil (dry weight) per replicate. This amount of soil per replicate was sufficient to represent soil diversity, as reflected in a low mean relative standard deviation (RSD). The RSD was calculated for each time point across the three replicates, yielding a mean RSD of 5.9% ± 6.2% across all time points and soils. 10 μL of the mixed stock solution and 10 μL of the linearized surfactins stock solution were added to one-quarter of the soil (corresponding to 1.25 g dry weight) in a 50 mL centrifugation tube. After 30 min, the rest of the soil (3.75 g on a dry weight basis) was added and mixed. This resulted in a final lipopeptide concentration of 2 mg/kg dry soil for each lipopeptide family, which is at the lower range of a pesticide application scenario.^45^ For initial time points, the lipopeptides were directly spiked to 5 g of soil (dry weight). The tubes were then closed and incubated at room temperature and aerated once a week for 1 h.

Lipopeptides were extracted (sacrificial samples) from the soils at 0, 1, 2, 4, 7, 10, 14, 20, 28, 40, 55, 70, and 102 days. Therefore, 10 mL of 70% acetonitrile (aq.) was added to the tubes containing the soil, and the sample was briefly vortexed and subsequently mixed on a horizontal shaker at 150 rpm for 30 min. The sample was then centrifuged at 7000*g* for 5 min, and the supernatant was diluted 1:1 with 70% MeOH (aq.). After another centrifugation at 16,000*g* for 2 min, the sample was transferred to an HPLC vial and kept at −20 °C until analysis. We evaluated the recovery of all lipopeptides from soil using the initial time point (0 days) of soil incubations.

### HPLC-HRMS Analysis

We analyzed lipopeptides using high-performance liquid chromatography (Vanquish Horizon UHPLC System, Thermo Fisher) coupled with a high-resolution mass spectrometer (HRMS) (Orbitrap Exploris 240, Thermo Fisher). Separation was achieved using a C18 column (XSelect Premier CSH, 2.5 μm, 2.1 × 150 mm, Waters), with an injection volume of 20 μL, a flow rate of 0.4 mL/min, a column compartment temperature of 40 °C, and the following eluents: (A) water with 0.1% (v/v) formic acid and (B) acetonitrile with 5% (v/v) water and 0.1% (v/v) formic acid. The eluent gradient was as follows: 0–2.5 min: 5% B, 2.5–10.5 min: 5–100% B, 10.5–13 min: 100% B, 13–13.3 min: 100–5% B, 13–16 min: 5% B. The MS parameters were set as follows: ion source: heated electrospray ionization, sheath gas: 50, aux gas: 10, sweep gas: 1, ion transfer tube temperature: 320 °C, vaporizer temperature: 300 °C, spray voltage: +3500 V, EASY-IC: Scan-to-Scan, MS full-scan: range: 100–1500 *m*/*z*, resolution: 90,000, AGC target: 10^6^, maximum injection time: AUTO, time between full-scan: 0.45 s, data dependent MS/MS: resolution 22,500, AGC target: 10^5^, maximum injection time: AUTO, isolation window: 1.0 *m*/*z*, NCE: 15,25,40,50.

Lipopeptides were identified based on exact mass (±3 ppm), retention time, and MS/MS spectra and considered for our investigation if the peak shape in a 10 μg/L calibration sample was sufficient for integration (Text S1 and Figures S2 and S15).

### Data Analysis

Parent lipopeptides were semiquantified in Skyline (version 22.2.0.527) using calibration curves that were either linear or quadratic with a 1/x weighting factor. The calibration range was selected such that all calibration solutions had an accuracy of 80–120%, and the limit of quantification (LOQ) was defined as the lowest selected calibration level. Instrument drift was corrected by using repeated injections of a single calibration solution at regular intervals (<4 h) throughout the measurement sequence. We note that due to the microbial origin of the lipopeptides, we obtained mixtures as standards (Figure S1), and in order to quantify lipopeptides, we assumed uniform ionization efficiency, thus only achieving semiquantification. To account for this limitation, we report the relative concentrations in progress curves.

For all lipopeptides, soil half-lives were determined by either a 1-pool or 2-pool first-order model. A fit was applied to the lipopeptide progress curves with the following formulas

$$1-pool-model:{\left[IP\right]}_{t}={\left[IP\right]}_{0}\times {e^{-}}^{k*t}$$

$${2-pool-model:\left[lp\right]}_{t}=\left({\left[LP\right]}_{0,bioavailable}-{\left[LP\right]}_{0,sorbed}\times \frac{k_{sorbed}}{\left(k_{bioavailable}-k_{sorbed}\right)}\right)\times {e^{-}}^{k_{bioavailable}*t}+{\left[LP\right]}_{0,sorbed}\times \left(1+\frac{k_{sorbed}}{\left(k_{bioavailable}-k_{sorbed}\right)}\right)\times {e^{-}}^{k_{sorbed}*t}$$

where [LP]_0_ is the initial concentration (i.e., the recovered amount), *t* is the time, and *k* is the first-order rate constant. The 2-pool model was used if the initial sorbed fraction was >10% and the relative standard errors (RSE) for each modeled variable stayed below 400%. The RSE criterion was selected so that outlier models with an unreasonably high confidence interval were discarded, which also resulted in overestimated half-life values compared to a visual estimation. The confidence interval widths for all 2-pool fits are shown in Figures S3–S5. Parameter uncertainties for the 2-pool model were large despite tight confidence bands around the median fits, which we ascribed to possible trade-offs between initial pool sizes and rate constants (i.e., equally good fits achieved by compensating a larger initial pool with a lower rate constant, and vice versa). Consequently, pool sizes could not be determined, and half-lives were calculated numerically from the fitted models by a parametric Monte Carlo simulation. Specifically, 5000 parameter sets were generated based on the estimated model parameters and their variance–covariance matrix. For each set, the time required to reach 50% of the initial concentration was determined numerically by root finding. Simulations that did not yield a valid solution were excluded. The resulting distribution of half-lives was summarized by their medians and standard errors (68% confidence intervals). A lag phase was reported, and the fit was delayed accordingly, if the concentration remained above a predefined threshold based on the initial concentration. Thresholds of 85%, 90%, and 95% were evaluated (Table S2). Thresholds of 85% and 90% resulted in comparable lag phases, with longer lag phases only in a few cases for the 85% threshold. In contrast, the 95% threshold resulted in inconsistent (across homologues) lag phases, especially for surfactins in soil 2.2. Therefore, a threshold of 90% was selected as the highest threshold, providing maximally reliable lag phase detection. The fit was applied until the lipopeptide concentration decreased to 5% of the starting concentration.

TP analysis was conducted using mzmine (version 4.8.30). Features were kept if they were absent in blanks and at the initial time point (*t*_0_), unless they increased later by at least 3-fold. Additionally, features had to be detected in at least nine sacrificial samples. This workflow was applied for every soil and soil condition (i.e., active and autoclaved) separately. Complementarily, we compiled a suspect list comprising all possible TPs that were derived from hydrolyses of the parent lipopeptides. Potential TPs were selected if they (i) occurred in the suspect list or (ii) were among the 10 most intense features with progress curves, which either increased during incubation or first increased and later decreased. Structures and parent compounds were assigned based on MS/MS spectra (Figure S16). Relative quantification of TPs was performed in Skyline, analogous to the workflow for parent compounds.

## Results and Discussion

### Strong Differences in Soil Half-Lives Between and Within Lipopeptide Families

As a basis for our in-depth biotransformation analyses, we first characterized the lipopeptides contained in the commercially obtained samples for each of the three investigated lipopeptide families. Such precharacterization is essential to resolve structure-dependent biotransformation behavior. Surfactin and iturin samples contained homologues spanning different alkyl-chain lengths (C-12–C-16 for surfactin and C-14–C-16 for iturins), whereas a single compound (C-16) was detected in the fengycin sample. Further details are provided in Text S2 and Figure S1. We evaluated the recovery for all lipopeptides from soil using the initial time point of soil incubations. Recoveries (concentration at 0 days) across all soils ranged between 67 and 90%, 53 and 73%, and 70 and 92% for surfactins, fengycins, and iturins, respectively (Figure S6). The unaccounted lipopeptides are likely irreversibly sorbed to the soil. Although incomplete, recoveries were reproducible across replicates, supporting robust quantification of temporal trends. We confirmed that no detectable background levels of lipopeptides were present in the tested soils by analyzing unspiked soil samples.

Incubation experiments with active soils revealed that all lipopeptides were substantially removed during the incubation time, but surfactins, fengycins, and iturins showed strongly distinct kinetics (Figures 2 and S7–S9). Across all soils, iturins were removed rapidly in a smooth, single-phase manner. Fengycins were removed more slowly but following similar kinetics. By contrast, surfactins were degraded considerably more slowly and showed specific kinetics depending on the soil: in soil 2.2, the only acidic soil tested, surfactins exhibited a pronounced lag phase of up to 2 weeks (Table S3), suggesting microbial adaptation as a prerequisite for degradation under these conditions. In soils 5 M and 6 S, surfactins followed a biphasic transformation pattern, with a distinct slower part after 4 weeks. This biphasic behavior indicates the formation of a less bioavailable fraction that limits further degradation. Consequently, in soil 6 S, a substantial fraction (21% ± 7%) remained after 102 days of incubation. These differences indicate that lipopeptide biotransformation is strongly structure-dependent across families. Soil incubation experiments of lipopeptides in autoclaved soil showed no or substantially delayed removal compared to active soil, confirming that lipopeptide transformation was predominantly biologically mediated. The logistic shape of the progress curves of the delayed removal in some autoclaved soil incubations suggests that the removal is mediated by regrown microbes; however, contribution from abiotic transformation or sorption cannot be excluded.

**Figure 2:**
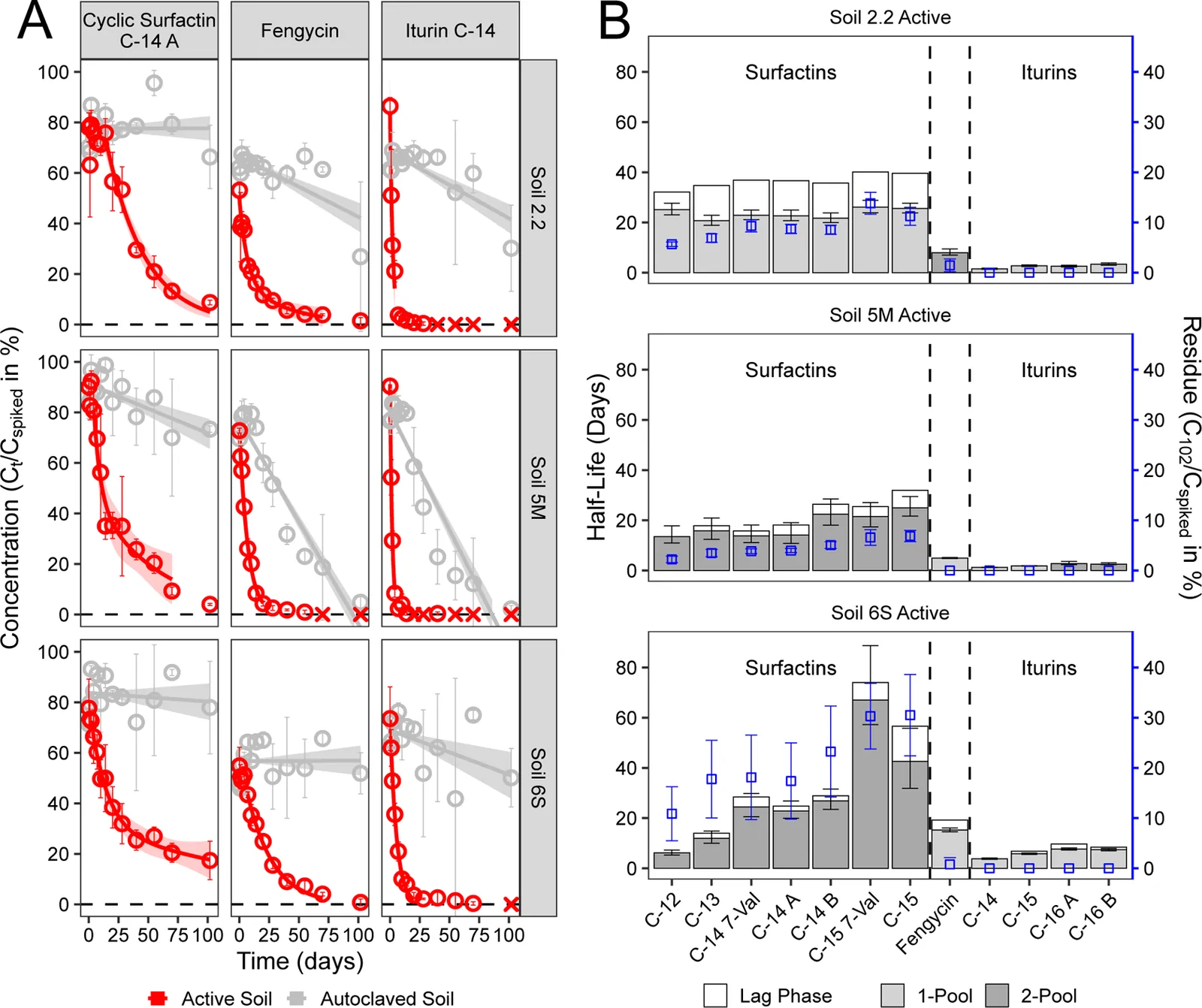
(A) Concentration progress curves of selected lipopeptides from all tested families during their incubation in active (red) and autoclaved (gray) soils. Data points and error bars represent means and standard errors, respectively, of triplicate sacrificial extractions for each time point. “X” marks values below the LOQ (dashed line). Solid lines represent fitted 1-pool or 2-pool first-order kinetic models (see text for details) for active soils and linear fits for autoclaved soils. The 95% confidence interval of these fits is shown as a ribbon. The fitted kinetics were adjusted to account for the lag phases observed in certain incubation experiments. (B) Half-lives of lipopeptide removal and residual lipopeptide concentrations derived from incubation experiments in active soils. Bar plots show half-lives (left *y*-axis) determined numerically from 1-pool (light gray) or 2-pool (dark gray) fitted kinetics (with bars and error bars representing means and standard errors), together with the quantified lag phase (white). Residual concentrations after 102 days (blue, right *y*-axis) with squares and error bars representing means and standard errors of triplicate extractions.

On the basis of the observed biphasic transformation, we fit a 2-pool (sorbed-bioavailable) first-order model (used here as an operational description of biphasic behavior, rather than a direct quantification of sorbed and bioavailable fractions) to each lipopeptide progress curve (see Section 2.5). Meaningful fits (i.e., *C*_sorbed_ > 10% and RSE <400%) were obtained for all surfactins in soils 5 M and 6 S, fengycin in soil 2.2, and iturin C-16 A/B in soil 5 M (Table S3). For the remaining compound–soil combinations, the data were well described by a 1-pool first-order kinetic model.

Across lipopeptide families, iturins exhibited the shortest half-lives in all soils (1–8 days), followed by fengycins (5–15 days) and surfactins (6–67 days) (Figure 2 and Table S3). The half-lives increase in accordance with the fraction of hydrophobic amino acid residues incorporated (iturins: 1/7, fengycin: 4/10, and surfactins: 5/7) (Figure 1), indicating that hydrophobicity may contribute to the observed differences in stability, likely through increased sorption and reduced bioavailability. The greater stability of fengycins over iturins suggests that rapid cleavage of the noncyclic alkyl chain or noncyclic amino acid residues is not the dominant transformation pathway under the tested conditions, contrary to our expectation.^33^ Also, the macrocyclic ester linkages in surfactins and fengycin do not exhibit faster apparent transformation compared to the macrocyclic amide linkages in iturins, despite esters generally being less stable than amides under environmental conditions.^46^

Comparing half-lives across lipopeptides with varying alkyl chain lengths but otherwise identical structures, we found that half-lives consistently increased with increasing alkyl chain length. Longer alkyl chains are expected to increase hydrophobicity, which was reflected by higher liquid chromatography retention times used here as a proxy for hydrophobicity. This suggests that hydrophobicity may contribute to the observed differences in stability, consistent with the trend observed across the different lipopeptide families. For iturins, half-lives increased for each additional CH_2_ unit by 31% ± 40%, 45% ± 6%, and 37% ± 13% for soils 2.2, 5 M, and 6 S, respectively. These increases did not differ significantly across all soils (*p* > 0.05). For surfactins, the increased half-lives were heavily influenced by the soil type, with an increase for each additional CH_2_ unit by 6% ± 12%, 19% ± 26%, and 110% ± 35% for soils 2.2, 5 M, and 6 S, respectively. The increase observed in soil 6 S was significantly greater than in soils 2.2 and 5 M (*p* < 0.001), whereas rates in soils 2.2 and 5 M did not differ significantly from each other (*p* > 0.05). This large influence of the alkyl chain length on the half-life in soil 6 S likely reflects an increased adsorbed pool size with increasing alkyl chain length. Beyond alkyl chain length, we did not observe any additional strong and consistent trends for minor structural variations: differences in branching of alkyl chains of the same length (i.e., between surfactin C-14 A and C-14 B and between iturin C-16 A and C-16 B) did not affect half-lives in a consistent manner. Likewise, substitution of leucine by valine at position 7 in surfactins had no consistent influence on the detected half-lives across soils.

Across the three investigated soils, half-life patterns of lipopeptide families were similar in soils 2.2 and 5 M but differed in soil 6 S. For fengycins and iturins, half-lives in soils 2.2 and 5 M were comparable, whereas in soil 6 S, half-lives were 2–3 times longer. For surfactins, we observed a different pattern with half-lives increasing from 18 ± 4.8 days in soil 5 M to 23.6 ± 2.1 days in soil 2.2 to 29 ± 20 days in soil 6 S. Notably, surfactins’ half-lives in soil 6 S had a significantly larger variability between homologues than in the other soils, encompassing the shortest and longest observed half-lives for surfactins (6–67 days). We speculate that these overall slower and more variable half-lives in soil 6 S, especially compared to soil 5 M, are caused by interactions between hydrophobic lipopeptide parts and hydrophobic sorption sites of the organic matter. This is consistent with the 40% higher organic matter content in soil 6 S compared to soil 5 M (Table S1). Also, second phase transformation rate constants were considerably slower in soil 6 S (*k*: 5.7 ± 2.5 * 10^–3^/day) compared to soil 5 M (*k*: 19.3 ± 1.1 * 10^–3^/day), with remarkable consistency across homologues. The absence of a strong biphasic transformation in soil 2.2, besides a slightly higher organic carbon content than in soil 6 S, suggests that other soil-specific factors (e.g., pH or organic matter composition) are also influencing the sorption of lipopeptides in soil.

Compared with the literature half-lives of protein-derived peptides in soil, surfactins and fengycins are considerably more stable. In a comparable experimental setup, substantially larger proteins (65, 75, and 120 kDa) exhibited mineralization half-lives of approximately 5 days.^47^ In our study, parent-compound half-lives (averaged across homologues) were 3.6–5.8× longer for surfactins and up to 3× longer for fengycins, despite the observed half-lives for parent removal being typically shorter than for complete mineralization. This result is consistent with the expected stabilization through the incorporation of cyclization, d-amino acid residues, and alkyl chains in lipopeptides.^33,34,37^ To further address such stabilization, we assessed the isolated effect of cyclization on the example of surfactins.

### Cyclization Is a Major Driver of Surfactin Stability

To assess the isolated effect of cyclization on the stability of surfactins in soils, we produced chemically linearized surfactins by hydrolyzing the ester bond and subsequently incubated these linearized surfactins simultaneously with the cyclic variants in soil. In a control experiment where only cyclic surfactins were spiked to the soils, we did not detect these linear variants as transformation products, indicating that ester-hydrolysis-based ring opening is not a dominant transformation pathway under the tested conditions (Figure S10). Nonetheless, assessing the stability of linearized surfactins remains informative for two reasons. First, it is the most widely studied linear surfactin, with potential application on its own (e.g., to vary properties of cyclic surfactins).^48–50^ Second, it is fundamentally interesting far beyond these applications to understand how susceptibility to biotransformation of lipopeptides is affected by a loss of the rigid cyclic backbone.^43,48–50^

Across all soils, recoveries of linear surfactins range from 54 to 68% (Figure S6), which is lower than the recovery range for the cyclic variants (67–90%). This reduced recovery is consistent with more available sorption sites for the more flexible linear structures. Soil incubation experiments in active soils revealed that all linear lipopeptides were completely removed during the incubation time (Figures S7–S9). Transformation of linear surfactins was substantially faster than that of cyclic variants, particularly in soils 5 M and 6 S, demonstrating that cyclization markedly increases stability in soils. In autoclaved soil, linear lipopeptides showed greatly reduced or significantly delayed removal compared to active soil, indicating that removal of linear lipopeptides, similar to cyclic lipopeptides, is mainly biologically catalyzed. Specifically, in autoclaved soils 2.2 and 6 S, linear lipopeptides were removed steadily at a slow rate over the incubation time, suggesting abiotic transformation. In autoclaved soil 5 M, concentrations were initially stable with a logistic removal after 2 weeks, suggesting regrowth of not completely inactivated microorganisms and subsequent biotransformation; however, contribution from abiotic transformation or sorption cannot be excluded (Figure S8).

Akin to cyclic lipopeptides, we fitted a 1- or 2-pool first-order model to each linear surfactin progress curve to derive soil half-lives (Figure 3). As for cyclic lipopeptides, the 2-pool model is used here as an operational description of biphasic behavior rather than a direct quantification of sorbed and bioavailable fractions. Most progress curves were well described by a 2-pool model, except linear surfactins C-14 B and C-15 in soil 2.2 and C-15 in soil 6 S, for which a 1-pool model was fitted (Figures S7–S9 and Table S3). In soils 5 M and 6 S, linear homologues transformed substantially faster (by factors of 3.1–7.6) compared to their cyclic variants, demonstrating a strong stabilizing effect of cyclization. Resulting absolute half-lives were similar to values between 1.8 and 6.9 days in soil 5 M and 1.2–8.5 days in soil 6 S. In contrast, in soil 2.2, only linear surfactins with shorter alkyl chains were transformed faster than their cyclic variants, and absolute half-lives (4–23.4 days) for all homologues were considerably longer than in soils 5 M and 6 S. Interestingly, the 2 week lag phase observed for the cyclic variant was absent in soil 2.2. The slower second-phase transformation rate constants were enhanced compared to those for the cyclic variants. The rates increased by roughly 4× in soil 5 M and 10× in soil 6 S, respectively (Table S3). Consequently, after 102 days, no residual linear surfactins were detected in any of the tested soils (<5% of spiked amount). Half-lives increased with longer alkyl chain lengths by 82% ± 69%, 61% ± 14%, and 113% ± 54% per additional CH2 unit in soils 2.2, 5 M, and 6 S, respectively, indicating that hydrophobicity remains a key control even for linear variants. For cyclic variants, a comparable trend was only observed in soil 6 S (110% ± 35%).

**Figure 3:**
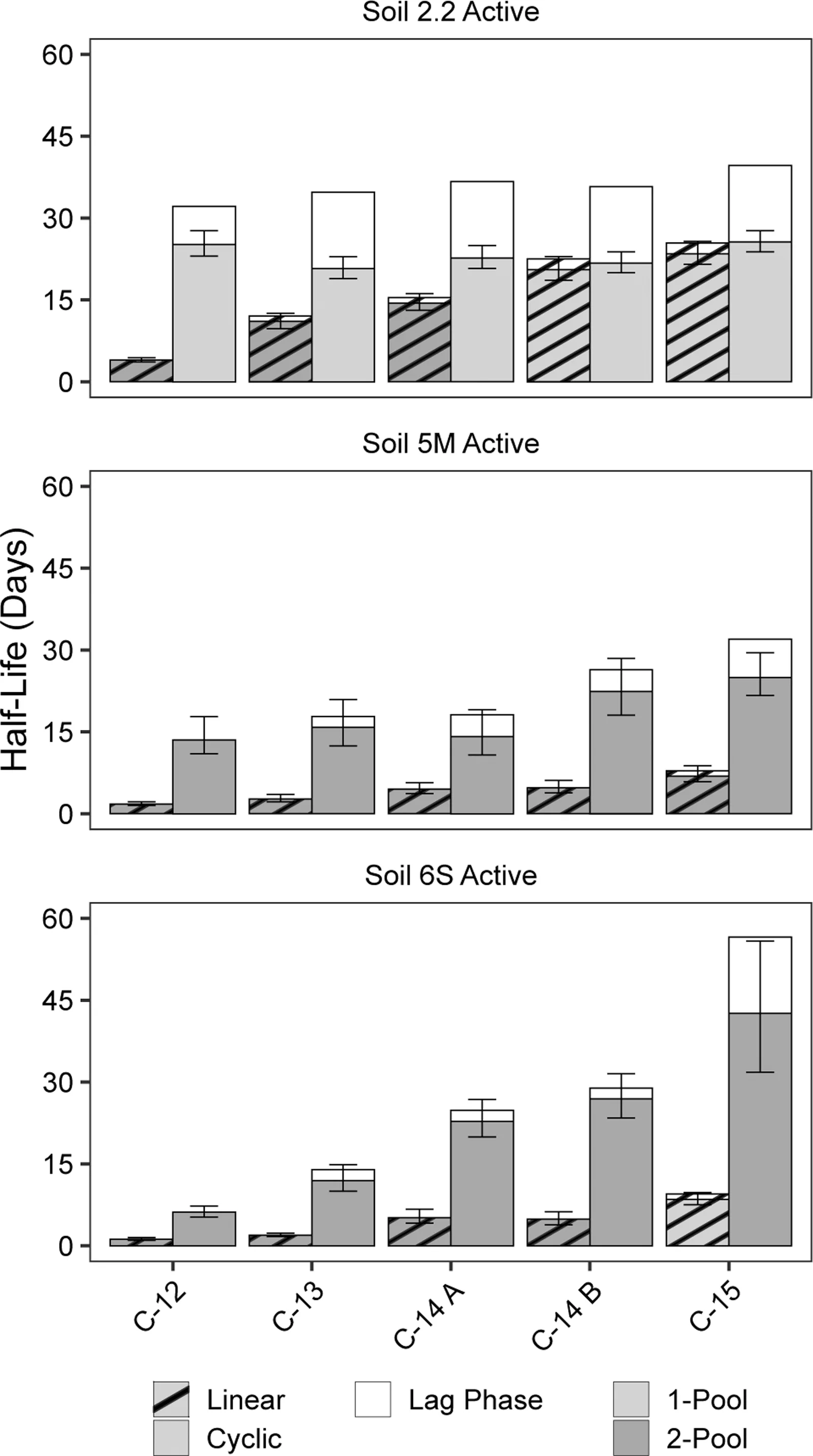
Half-lives of cyclic and linear lipopeptide removal derived from incubation experiments in active soils. Bar plots show half-lives determined numerically from 1-pool (light gray) or 2-pool (dark gray) fitted kinetics (with bars and error bars representing means and standard errors), together with the quantified lag phase (white) for linear (stripped) and cyclic (replotted from Figure 2) lipopeptides.

Our results indicate that cyclization is a major driver of surfactin stability in soils 5 M and 6 S. This is consistent with a cyclic scaffold that creates a hydrophobic side, which likely reduces both bioavailability, through enhanced sorption to soil components, and enzymatic susceptibility, through increased conformational rigidity.^35,36^ To differentiate between these two mechanisms, further work on lipopeptide sorption and transformation in isolated systems is needed. However, cyclization is not a major driver for surfactin stability in soil 2.2: the ∼2 week lag observed for the cyclic variant disappeared upon linearization, yet subsequent half-lives differed only for the variants with short alkyl chain lengths. This finding suggests that under certain conditions, biological factors (e.g., microbial adaptation and presence of suitable enzymes) can dominate over structure-driven effects.

### TP Analysis to Elucidate Lipopeptide Biotransformation Pathways

To investigate biotransformation pathways of lipopeptides and to identify potentially long-lived biodegradation intermediates, we combined a suspect screening approach and a nontarget analysis based on HRMS data. The suspect list encompassed all possible hydrolysis-derived transformation products (n = 310) for each parent lipopeptide. For the nontarget screening, we focused within each soil on the 10 most intense features that exhibited meaningful progress curves (i.e., either increased during the incubation time or first increased and later decreased). Additional features with signal intensities above 2 × 10^5^ are listed in Table S4. This prioritization ensures that only transformation products relevant to the overall biotransformation dynamics are considered. The transformation products were identified with a confidence level of 2b, unless otherwise stated (Text S1 and Figure S16).^51^

For fengycin and the major iturin variants (i.e., C-14 and C-15), we detected side chain modifications in the form of oxidation and nitration (Figure 4 and Text S1). Nitration in both lipopeptides occurred at a tyrosine residue, yielding a nitrotyrosine residue. Oxidation of iturin also occurred at the tyrosine residue and likely yielded a dopaquinone residue. Fengycin oxidation most likely occurred at the alkyl chain by hydroxylation and dehydrogenation (confidence level of 3). We speculate that the presence of a β-hydroxyl group in the alkyl chain, which only occurs in fengycin, promotes this oxidation pathway. These side-chain modifications were detected in most soils but varied in abundance without a consistent trend (Figure S11). While several of these transformation products were short-lived, nitrated fengycin in soil 2.2 was still detected after 102 days with a relative (area_TP_/area_parent_) intensity of 2%. We note that we did not detect linearized fengycin or iturins. It is possible that these linear forms could not be quantified with the extraction and analytical protocol used; however, as we did not see a large difference in the analytical behavior of cyclic and linear surfactin, we did not further optimize the method for linear lipopeptides. Another explanation could be that, similar to linear surfactins, these linear intermediates are also less stable than their cyclic precursors and, therefore, exhibit only low transient concentrations. Additionally, concentrations may be further reduced by the formation of multiple transformation products resulting from the combination of different initial hydrolysis (i.e., linearization) sites and side chain modifications.

**Figure 4:**
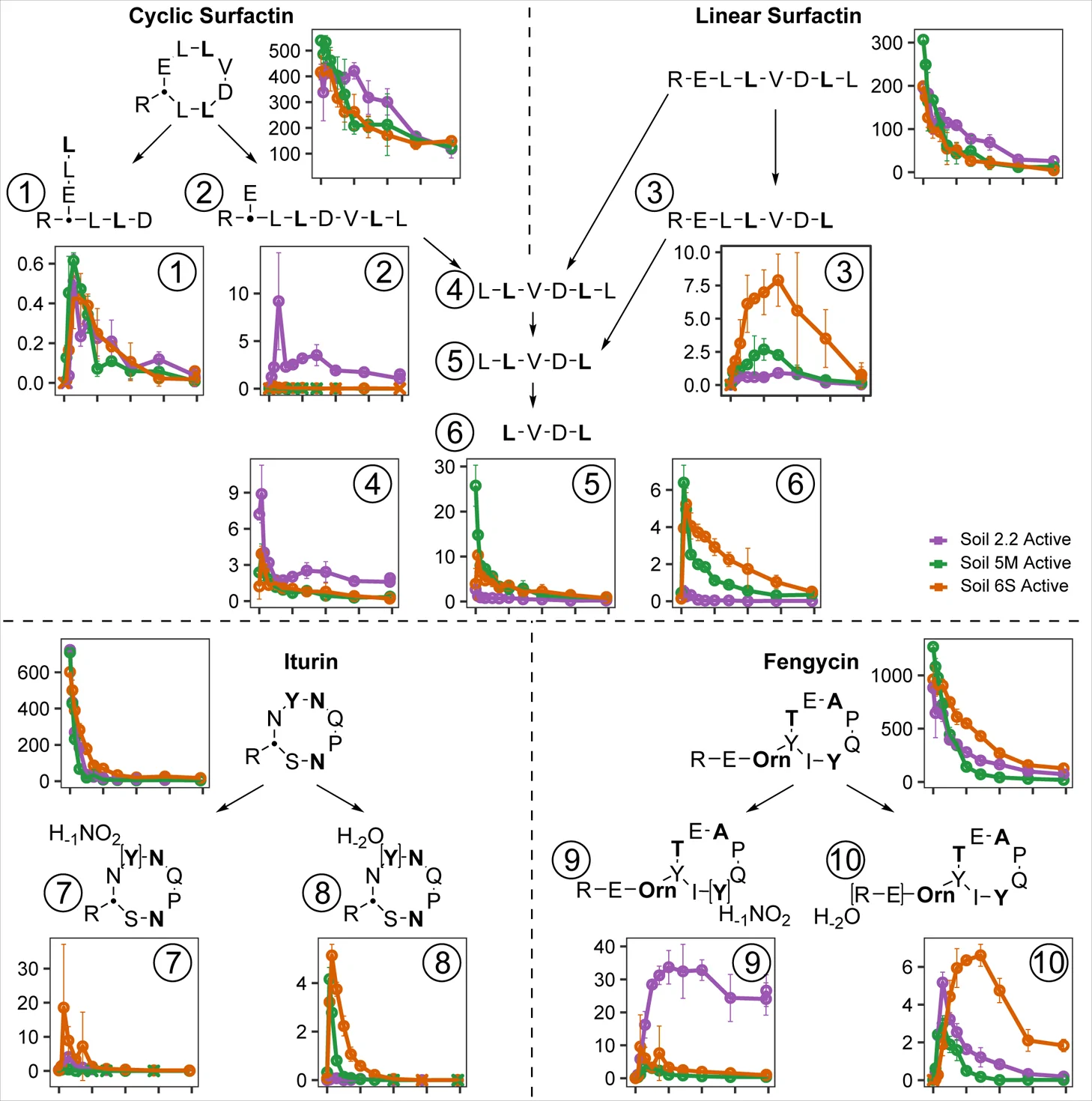
Proposed transformation pathways for lipopeptides during soil incubation. Progress curves display the peak area (a.u.) during 56 days and are shown for parent compounds next to the respective structure and for transformation products, as indicated by the numbering. Progress curves are shown for the C-14 A variant for surfactin, the C-16 variant for fengycin, and the C-14 variant for iturin. Amino acid residues are abbreviated with their one-letter code (except Orn for ornithine), d-amino acid residues are in bold, and *R* denotes the alkyl chain. “•” marks the cyclization point (i.e., ester or amide bond). H_–1_NO_2_ indicates addition of a nitro group, and H_–2_O indicates addition of oxygen.

For surfactins, we detected a set of short-lived intermediates that all lacked the alkyl chain and thus were common products to all surfactin variants (Figure 4). These products correspond to loss of *R*-Glu (LLVDLL), *R*-Glu-Leu (LVDLL), and the combination of 7-Leu and *R*-Glu-Leu (LVDL), where R denotes the alkyl chain (Text S1). This set of products is probably subsequently transformed from LLVDLL to LVDL. We confirmed through a control experiment that these products originated from both linear and cyclic variants, which were otherwise incubated simultaneously (Figure S12). Through this experiment, we could attribute ∼95% of the observed LLVDLL and LVDLL intensities and ∼80% of the LVDL intensity to production from linear surfactin, which is expected due to the faster turnover of the linear precursors. The transformation progress curves correlated well with those of the linearized surfactins, suggesting low stability in soil.

For linearized surfactins (except for the C-12 variant), we detected a major TP arising from loss of 7-Leu (*R*-ELLVDL, Figure S13). *R*-ELLVDL exhibits comparable or even greater stability than its respective parent compound (Figure 4). These products are probably precursors to the short-lived transformation products discussed above. The enhanced stability can be explained by the N-terminus of the respective *R*-ELLVDL being protected by the alkyl chain and the C-terminus by a d-amino acid residue, which reduces enzymatic susceptibility. Together with the observation that subsequent transformation products without an alkyl chain (i.e., LVDLL and LVDL) are short-lived, this indicates that dealkylation is the rate-determining step following initial hydrolysis (i.e., linearization) of surfactins. Notably, *R*-ELLVDL was least abundant in soil 2.2, with signal intensities reduced to 35% ± 13% and 10.6% ± 0.9% relative to soils 5 M and 6 S, respectively. This reduced transformation rate suggests slower leucine cleavage in soil 2.2, which potentially explains the overall longer half-life of linearized surfactin in soil 2.2 compared to the other soils.

For cyclic surfactins, we detected one linearized surfactin (LLVDLL-R-E, Figure S14) mainly in soil 2.2 and to a lesser extent in soil 6 S. In soil 2.2, the intensity of this feature increased during the lag phase, in which only a small fraction of cyclic surfactins was transformed. We speculate that this TP was rapidly transformed in soils 5 M and 6 S but accumulated in soil 2.2 because the microbial community has not yet fully adapted to metabolize surfactin. Additionally, we detected in all soils a product resulting from loss of valine (confidence level 3), indicating prior linearization next to 4-valine. This observation indicates that linearization occurs at multiple cleavage sites and is not restricted to a single pathway. We note that further extending incubation times beyond 100 days might result in the detection of additional, slowly formed and stable, transformation products.

## Environmental Implications

Our results show that the stability of lipopeptides in soils is strongly affected by their chemical structure, both between and within the tested lipopeptide families. Our findings on this structure–stability aspect are important to consider when selecting lipopeptides (and in most cases lipopeptide mixtures rather than pure single compounds) for their agricultural application as biopesticides. While some of the tested lipopeptides were readily transformed across the three tested agricultural soils (i.e., half-lives in the order of a few days), others were transformed with half-lives reaching up to months, with a substantial fraction of the spiked amount remaining after ∼100 days of incubation. This range indicates that not all lipopeptides are rapidly biodegradable. Compared to approved pesticides in the European Union, which have a median reported half-life of 24 days, the herein investigated lipopeptides, with a median half-life of 13 days, were overall slightly less stable.^52^ Across lipopeptide families and chain length variants, we also observed variability between the three different soil types tested. Most notably, surfactin transformation in clay-rich soil (i.e., LUFA 6 S) was slowest, consistent with stronger sorption and reduced bioavailability in this soil. Further research into the sorption of lipopeptides to individual soil constituents is, however, needed to establish broader relationships between soil properties and lipopeptide biotransformation. Additionally, to enable lipopeptide biodegradation predictions beyond the investigated soils, the identification of the key enzymes involved in this process is needed.

Our mechanism-focused investigation of linearized and cyclic surfactin showed that cyclization substantially increases surfactin stability in soil. Additionally, we observed that the presence of an alkyl chain increases stability and that the effect is strongly influenced by the length of the alkyl chain. Our investigation on the stability of transformation products showed that while many transformation products were short-lived, some low-intensity transformation products were more stable than their parent compounds. This finding highlights the need for further research on the fate and effects of lipopeptides to inform environmental risk assessments, particularly because some transformation products, such as linearized surfactins, are known to retain bioactivity, although at reduced levels.^49^ Together, our study provides insights into structure–stability relationships and biotransformation pathways of relevant lipopeptides in soils and can thus offer practical guidance for selecting and potentially even (re)­designing lipopeptide biopesticides with improved soil biodegradability (e.g., favoring shorter over longer alkyl chains or favoring fengycins and iturins over surfactins). We anticipate that this study on environmental *Bacillus* lipopeptide fate will also inform further studies in environmental systems beyond soils, where lipopeptide release is expected based on their use in personal care products,^53^ household detergents,^54,55^ and medical applications.^14^

## Supplementary Material





## Notes

Machine-readable information on transformation pathways and kinetics (ZIP)
Lipopeptide structure elucidation; characterization of investigated lipopeptides; mixture composition of investigated lipopeptides; LC–MS chromatograms of lipopeptide calibration solution; lipopeptides recovery from soil; confidence interval widths for 2-pool model in soil 2.2, 6 S, and 5 M; concentration of lipopeptides during their incubation in soil 6 S, 5 M, and 2.2; concentration of cyclic surfactins and linearized analogs during soil incubation, in which only cyclic surfactin was spiked; peak areas of transformation products of iturins and fengycins during soil incubation; intensities of cyclic and linear surfactins transformation products during soil incubation, in which only cyclic surfactin was spiked; peak areas of transformation products of linear surfactins during soil incubation; peak areas of transformation products of cyclic surfactins during soil incubation; annotated MS/MS spectra of investigated lipopeptides; annotated MS/MS spectra of transformation products; soil parameters; calculated lag phases; results of first-order kinetic fit; features, detected by nontarget screening, without tentative structure (PDF)
