# Supplementary material for: Chemical Structure Controls Soil Biotransformation Kinetics of Bacillus Lipopeptide Biopesticides
Source: Environ Sci Technol. 2026 Sep 25;60(40):29043–53. doi: 10.1021/acs.est.6c06707 (PMC13674060; doi:10.1021/acs.est.6c06707)
Supplement: Supplementary file 1 [file es6c06707_si_002.pdf]

Supporting Information

Chemical Structure Controls Soil Biotransformation Kinetics of Bacillus Lipopeptide Biopesticides

Richard Gruseck[1,2], Thilo Hofmann[1], Michael Zumstein[1*]

[1]University of Vienna, Department of Environmental Geosciences, Centre for Microbiology and Environmental Systems Science, Vienna, 1090, Austria

[2]University of Vienna, Doctoral School in Microbiology and Environmental Science, Vienna, 1030, Austria

[*]To whom correspondence should be addressed:

E-mail: michael.zumstein@univie.ac.at

This PDF file includes:

Text SI 1 Structure elucidation by MS/MS spectra

Text SI 2 Characterization of investigated lipopeptides.

Figure SI 1 Mixture composition of investigated lipopeptides.

Figure SI 2 LC-MS chromatograms of lipopeptide calibration solution.

Figure SI 3: Histogram of confidence interval widths for 2-pool model in soil 2.2.

Figure SI 4: Histogram of confidence interval widths for 2-pool model in soil 6S.

Figure SI 5: Histogram of confidence interval widths for 2-pool model in soil 5M.

Figure SI 6 Lipopeptide recovery from soil.

Figure SI 7 Concentration of lipopeptides during their incubation in soil 6S.

Figure SI 8 Concentration of lipopeptides during their incubation in soil 5M.

Figure SI 9 Concentration of lipopeptides during their incubation in soil 2.2.

Figure SI 10 Concentration of cyclic surfactins and linearized analogs during soil incubation, in which only cyclic surfactin was spiked.

Figure SI 11 Peak areas of transformation products of iturins and fengycins during soil incubation.

Figure SI 12 Intensities of cyclic and linear surfactin transformation products during soil incubation, in which only cyclic surfactin was spiked.

Figure SI 13 Peak areas of transformation products of linear surfactins during soil incubation.

Figure SI 14 Peak areas of transformation products of cyclic surfactins during soil incubation.

Figure SI 15 Annotated MS/MS spectra of investigated lipopeptides.

Figure SI 16 Annotated MS/MS spectra of transformation products.

Table SI 1 Soil parameters

Table SI 2: Calculated lag phases.

Table SI 3 Results of first-order kinetic fit.

Table SI 4: Features, detected by non-target screening, without tentative structure.

Text SI 1: Structure elucidation.

Surfactin structures were elucidated by MS/MS spectra (all masses refer to the surfactin variant with a C-14 alkyl chain and leucine at position 7) (**Figure SI 12**). We note that we could not distinguish between isoleucine and leucine. For better readability we will omit mention of isoleucine hereafter. Surfactins were generally identified based on the diagnostic fragment y6+H_2O : the fragment with m/z = 685.4492 contains one valine and four leucines, while the fragment with m/z = 671.4334 contains two valines and three leucines. The length of the alkyl chain was determined from the difference between the y6+H_2O fragment and the precursor (i.e., mz = 337.2257 for 14 carbon atoms). The present b-fragment series (with the exception of b5) and the information that Asp is at position 5 were used to identify the amino acid sequence[1]: R-Glu-Leu -Leu -Val-Asp-Leu -Leu/Val (b1: 356.2432, b2: 469.3272, b3: 582.4113, b4: 681.4797, b6: 909.5907). The difference between [M+H] and b6 was used to distinguish between surfactins with Leu or Val at position 7. Other fragments such as y6a3 (199.1805), y6b3 (227.1754), y3a6 (201.1234), y3b6 (229.1183), y5b5 (328.1867), y6b5 (441.2708), [M+H] – CO, and [M+H] - H2O were conserved across all variants. For linearized surfactin, we performed the analog analysis, using the same masses for the fragments, with the only difference being the increased mass of the precursor by 18.01056 (i.e., the mass of H_2O).

Iturin A was identified by fragments from the loss of up to three ammonia groups from the precursor, confirming the presence of several Asn and Gln amino acid residues (**Figure SI 12**). The amino acid sequence Pro-Asn-Ser-R-Asn-Tyr-Asn-Gln was identified based on the b-series (after ring opening between Pro and Gln, as shown by Yang et al.[1] All m/z values are given for a C-14 alkyl chain) consisting of b2 (212.1030), b3 (299.1350), b4 (638.3872), b5 (801.4504), and b6 (915.4934). The length of the alkyl chain was determined by the fragment y5b3 (313.2486) which contains the alkyl chain and Ser.

Fengycin A was identified based on the characteristic fragments y9 and y8 (m/z = 1080.5360 and 966.4567, respectively) (**Figure SI 12**). The length of the alkyl chain was determined based on the difference between the precursor and y9 (383.2672 for C-16). The amino acid sequence of the ring was determined by fragments after ring opening of the fragment y8 between Ala and Pro: b5 (-CO-NH3: 620.3079), b3 (389.1820), and b2 (226.1186) as well as y7b5 (-CO-NH3: 523.2551) and structural information from Yang et al.[1] The sequence was determined to be R-Glu-Orn-cyclo[Tyr-Thr-Glu-Ala-Pro-Gln-Tyr-Leu]

Transformation products of cyclic surfactins were identified as follows (**Figure SI 13**): the ion at m/z 1040.6853 (for C-14) and homologs for C-13 and C-15 corresponds to linearization between 1-Glu and 2-Leu confirmed by the y-series and the b4 fragment (H-Leu-Leu-Val-Asp). The ions at m/z 941.6169 (C-14) 955.6326 (C-15) correspond to the loss of valine. Although we could identify 4x leucine, 1x aspartic acid and the correct R-Glu group, the peptide sequence could not be aligned with the precursors sequence.

Transformation products of linearized surfactins were identified as follows (**Figure SI 13**): the ion at m/z 927.6013 (for C-14) and homologs for C-13 and C-15 corresponds to the loss of 7-leucine, confirmed by the unchanged b series (b4, b3, b2, and b1) compared to the precursor. The ion at m/z = 685.4495 is the diagnostic y6+H_2O fragment from surfactins, b4 and b2 fragments confirmed the structure of Leu-Leu-Val-Asp-Leu-Leu. Sequential losses of leucine yielded ions

at m/z = 572.3654 and 459.28133, the position of the cleaved leucine was identified by the b-series and y3, and y2 fragments respectively yielding Leu-Val-Asp-Leu-Leu and Leu-Val-Asp-Leu.

Transformation products of iturins were identified as follows (**Figure SI 13**): iturins were identified by fragments from the multiple losses of ammonia groups. The transformation product at m/z = 544.7722 (z=2, C-14) indicates nitration. The nitration position was located to be 5-tyrosine, supported by the increased b6 (+Δm/z 44.9851, +NO_2-H) and the unchanged b4 fragment, since only tyrosine and not asparagine can be nitrated the product is a nitrotyrosine residue. The transformation product at m/z = 529.2697 (z=2, C-14) indicates oxidation. The oxidation position was located to be 5-tyrosine by the increased y6 (z=1, +Δm/z 13.9792, +O-2H) and the unchanged b5 fragment. The tyrosine residue was likely oxidized to a dopaquinone residue.

Transformation products of fengycins were identified as follows (**Figure SI 13**): fengycins were identified by the characteristic y9 and y8 fragments. The transformation product at m/z = 754.8978 (z=2, +Δm/z = 22.4926) indicates nitration. The nitration site was located to 3-tyrosine because all fragments increased by +Δm/z 44.9851 (including b3: Pro-Gln-Tyr), except b2 (Pro-Gln) which remained unchanged. The transformation product at m/z = 739.3954 (z=2, +Δm/z = 6.9896, +O-2H) indicates oxidation. The oxidation site was located to be either at the alkyl chain or the glutamic acid residue because all fragments (from b2 to y9) remained unchanged. We speculate that hydroxylation and dehydrogenation close to the beta-hydroxyl group in the alkyl chain is the most likely transformation.

(1) Yang, H.; Li, X.; Li, X.; Yu, H.; Shen, Z. Identification of Lipopeptide Isoforms by MALDI-TOF-MS/MS Based on the Simultaneous Purification of Iturin, Fengycin, and Surfactin by RP-HPLC. *Anal Bioanal Chem* **2015**, *407* (9), 2529–2542. https://doi.org/10.1007/s00216-015-8486-8.

Text SI 2: Characterization of investigated lipopeptides.

The surfactin sample contained cyclic surfactins with alkyl chain lengths between C-12 and C-15 (**Figure SI 1**). For Surfactin C14 we found two peaks with similar MS/MS, which we attributed to different branching of the alkyl chain (**Figure SI 2, Figure SI 12**). We note that our liquid chromatography method did not fully separate all compounds, resulting in some broader peaks. This observation suggests that there are more branched variants which we summarized as one compound. As we did not detect any substantial changes of these peak shapes over time, we conclude that no distinguishable differences in transformation kinetics among these co-eluting variants occured. Two peaks had a significantly higher retention time than their respective isomers. Through MS/MS analysis we identified them as 7-Val variants compared to the dominant 7-Leu variant. Additional traces (e.g. four surfactins with a carbon alkyl chain of 16 carbons) were detected, but not at sufficiently high intensities to quantify them throughout the incubation experiments. Our surfactin mixture covers a broad range of alkyl chain diversity (C12-C17).[2]

We detected in the iturin sample variants with alkyl chain lengths between C-14 and C-16 (**Figure SI 1**). For Iturin C-16 we found two peaks with similar MS/MS, which we assigned to different branching of the alkyl chain (**Figure SI 2, Figure SI 12**). We note that our liquid chromatography method did not separate all compounds, resulting in some broader peaks (e.g. Iturin C-15). This observation suggests that there are more branching variants which we summarized under one compound. Iturin A is the major iturin out of seven known compounds in the iturin family. The detected alkyl chain lengths are covering the entire possible range.[2]

We detected in our fengycin sample fengycin A C-16. Fengycin A C-15 was also present but at too low intensities to quantify it in the incubation experiment (**Figure SI 2**). In our stock, we thus had limited chemical diversity. We note, however, that fengycin A is besides fengycin B the major fengycin variant and that an alkyl chain length of C-16 is most common, with ranges from C-14 to C-18.[2]

(2) Théatre, A.; Hoste, A. C. R.; Rigolet, A.; Benneceur, I.; Bechet, M.; Ongena, M.; Deleu, M.; Jacques, P. Bacillus Sp.: A Remarkable Source of Bioactive Lipopeptides. In *Biosurfactants for the Biobased Economy*; Hausmann, R., Henkel, M., Eds.; Springer International Publishing: Cham, 2022; pp 123–179. https://doi.org/10.1007/10_2021_182.

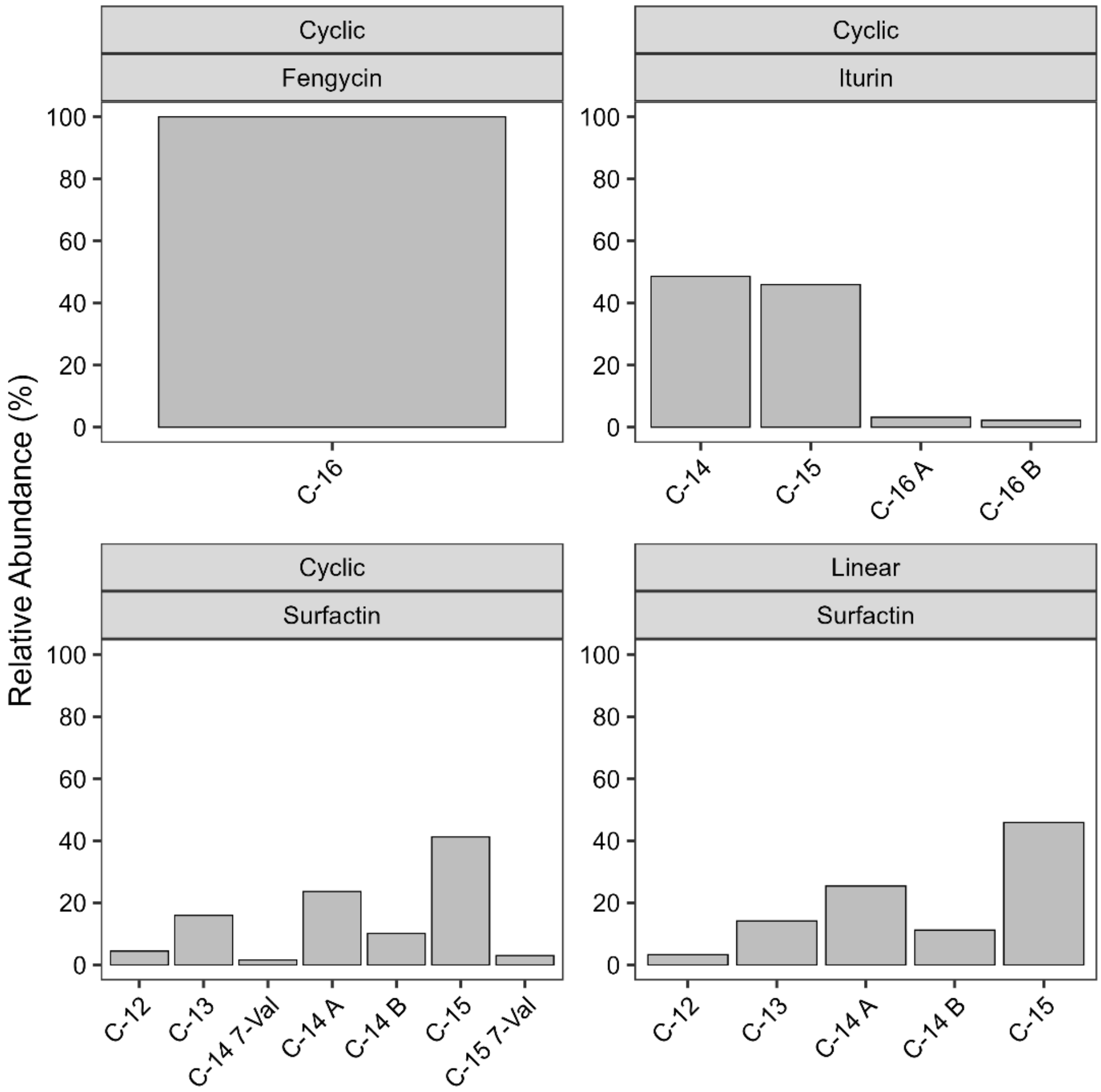

Figure SI 1: Composition of a 1 mg/L calibration solution showing the relative abundance of individual compounds based on peak area. For each lipopeptide family, peak areas were normalized so that the sum equals to 100%.

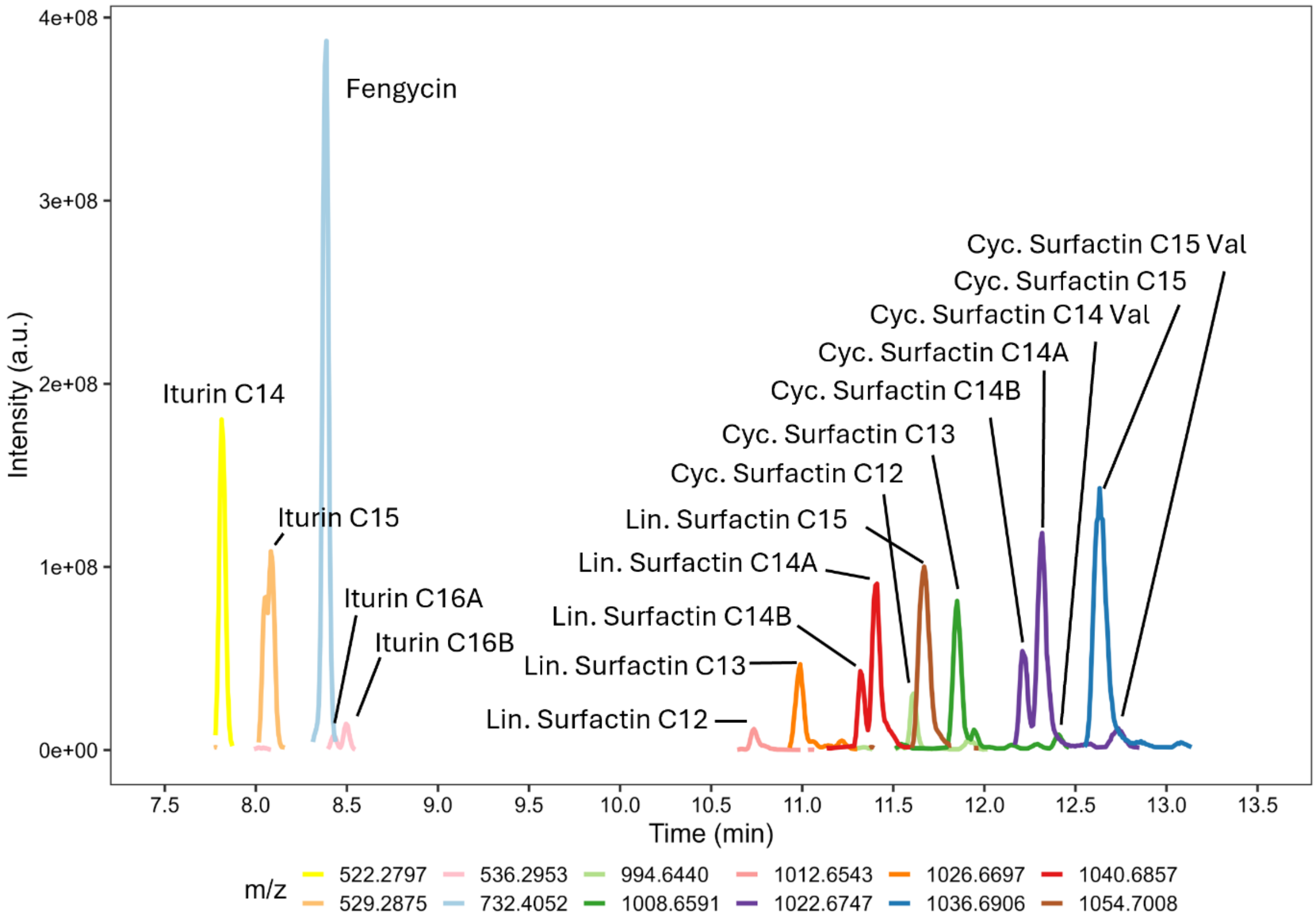

Figure SI 2: Extracted-ion chromatograms of a mixed lipopeptide calibration solution analyzed by HPLC-HRMS. Traces are cut off below 1% of the maximum of the respective extracted ion chromatogram. Compounds used for quantification in soil incubation experiments are labeled.

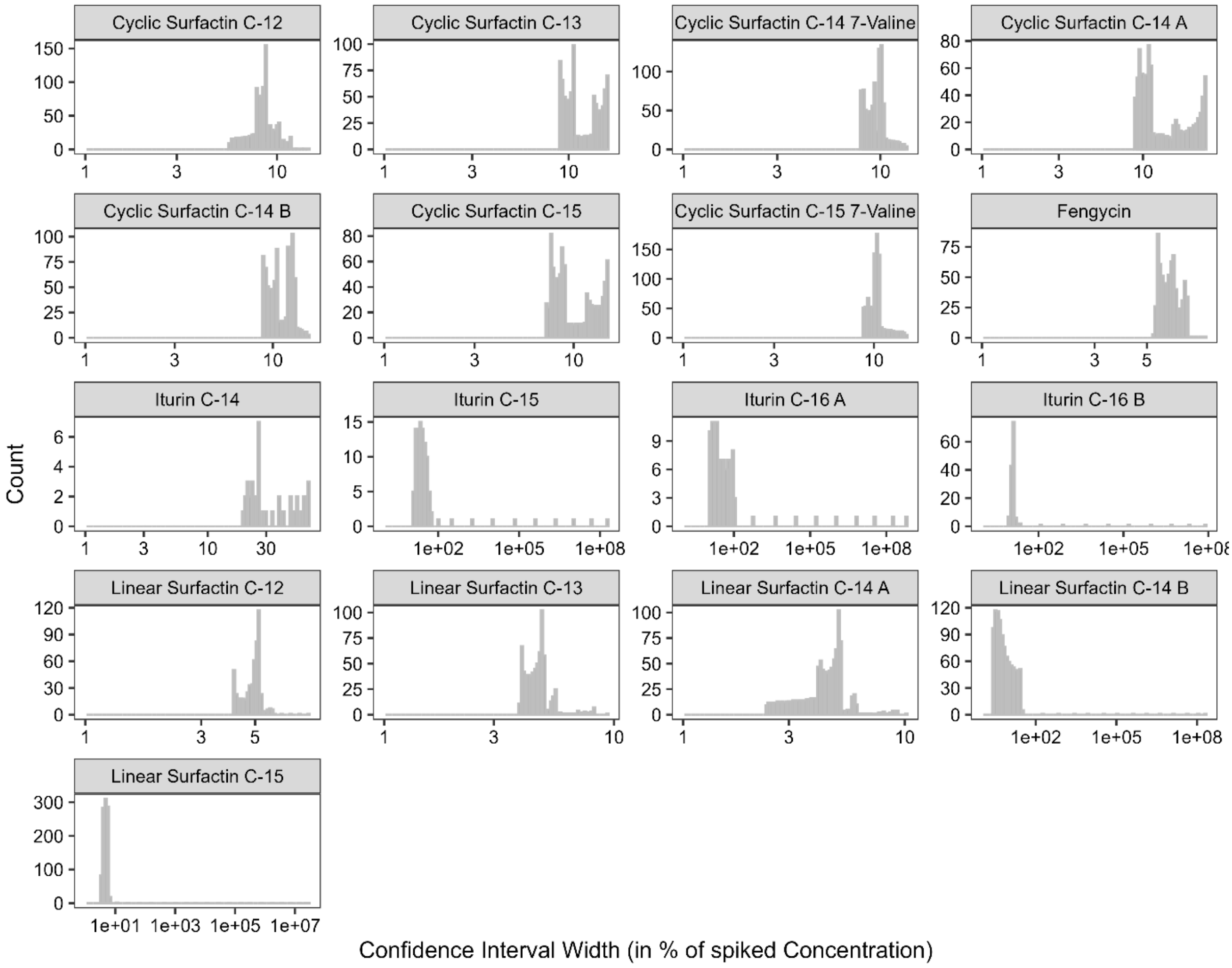

Figure SI 3: Histogram of confidence interval widths for 2-pool model in soil 2.2. Based on the criteria that the relative standard errors (RSE) for each modelled variable should stay below 400%, models with higher confidence interval widths (i.e., those for Iturin C-14, Iturin C-15, Iturin C-16 A, Iturin C-16B, linear Surfactin C-14, and linear Surfactin C-15) were discarded and the simpler 1-pool model fit was used.

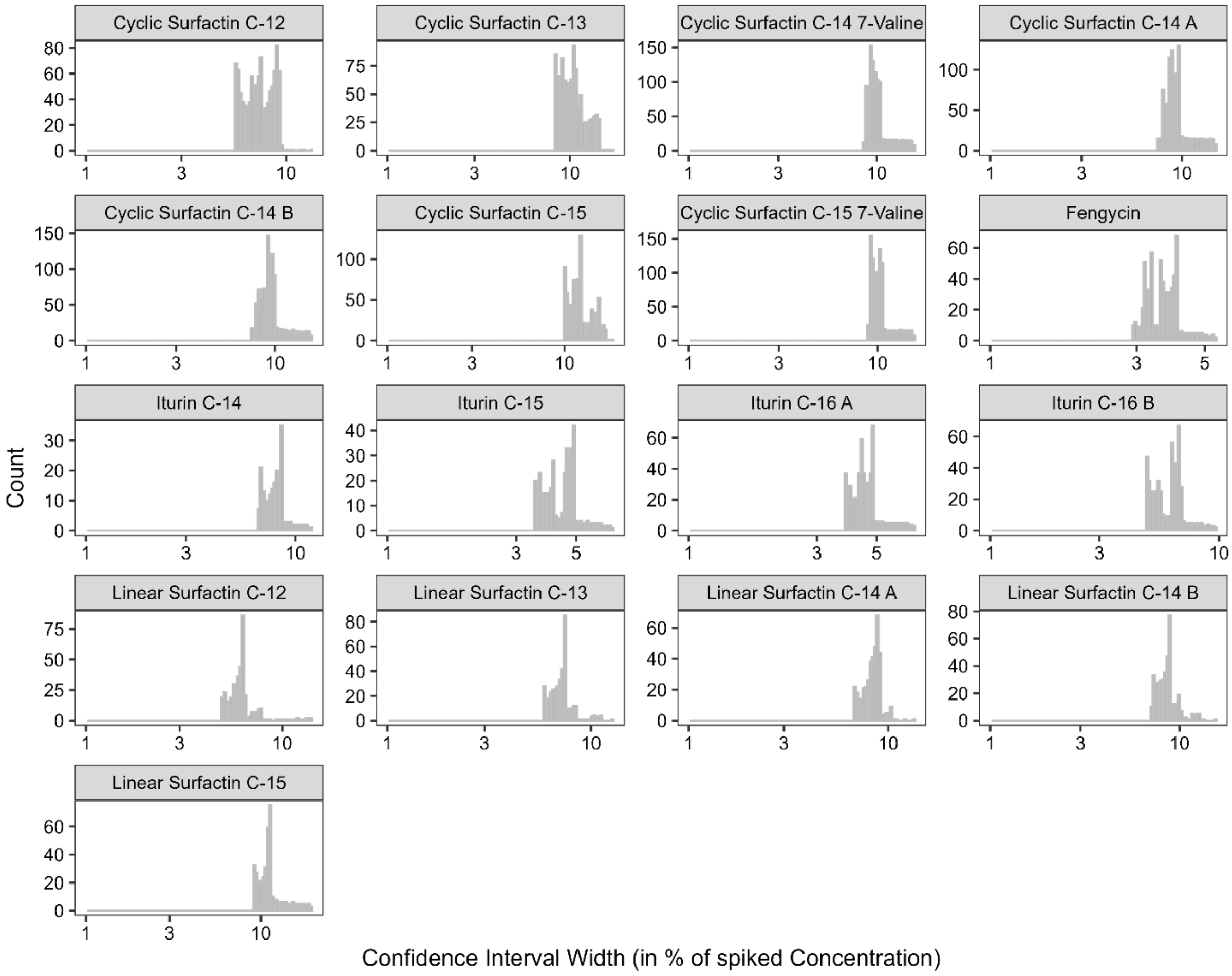

Figure SI 4: Histogram of confidence interval widths for 2-pool model in soil 6S.

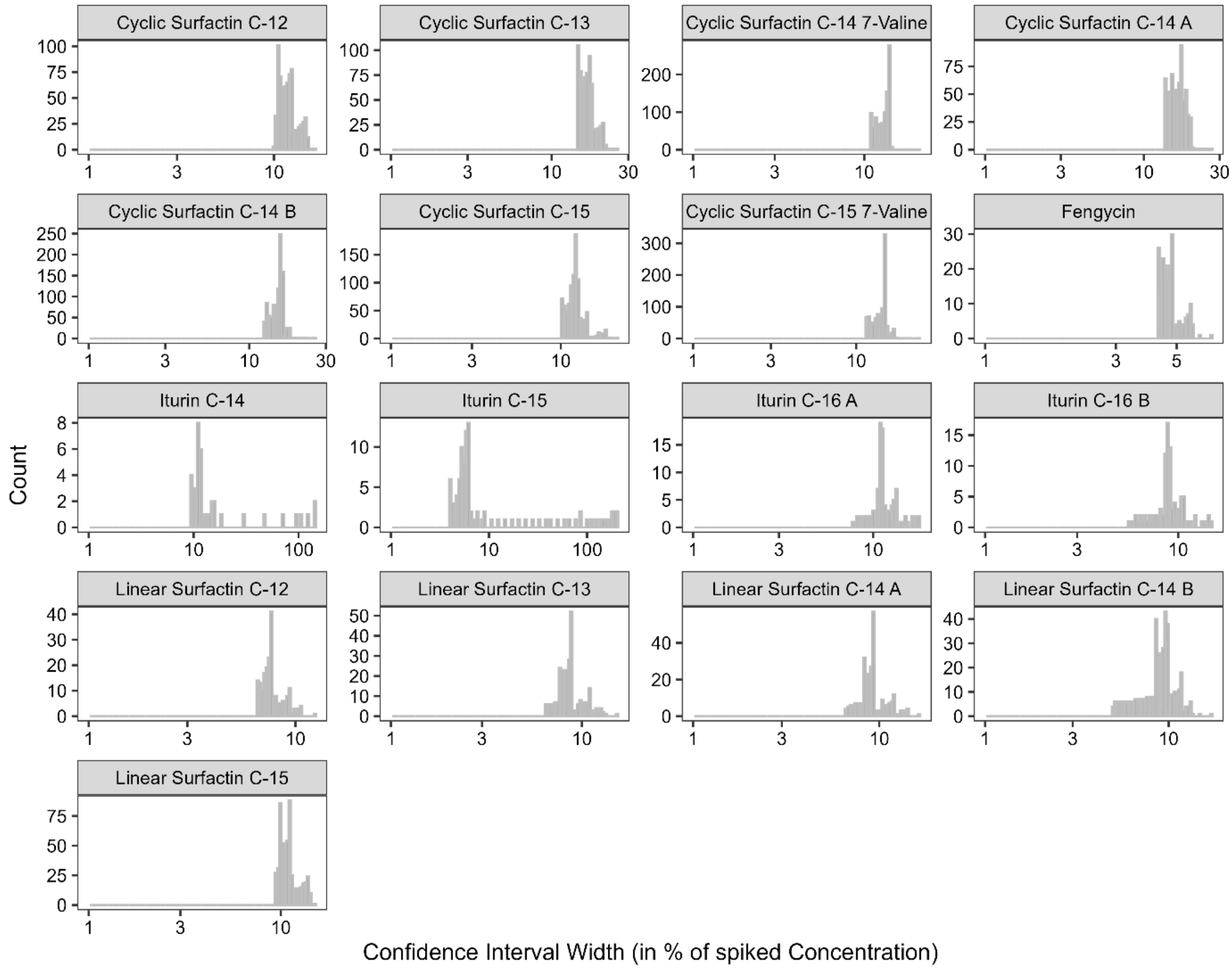

Figure SI 5: Histogram of confidence interval widths for 2-pool model in soil 5M. Based on the criteria that the relative standard errors (RSE) for each modelled variable should stay below 400%, models with higher confidence interval widths (i.e., those for Iturin C-14, Iturin C-15) were discarded and the simpler 1-pool model fit was used.

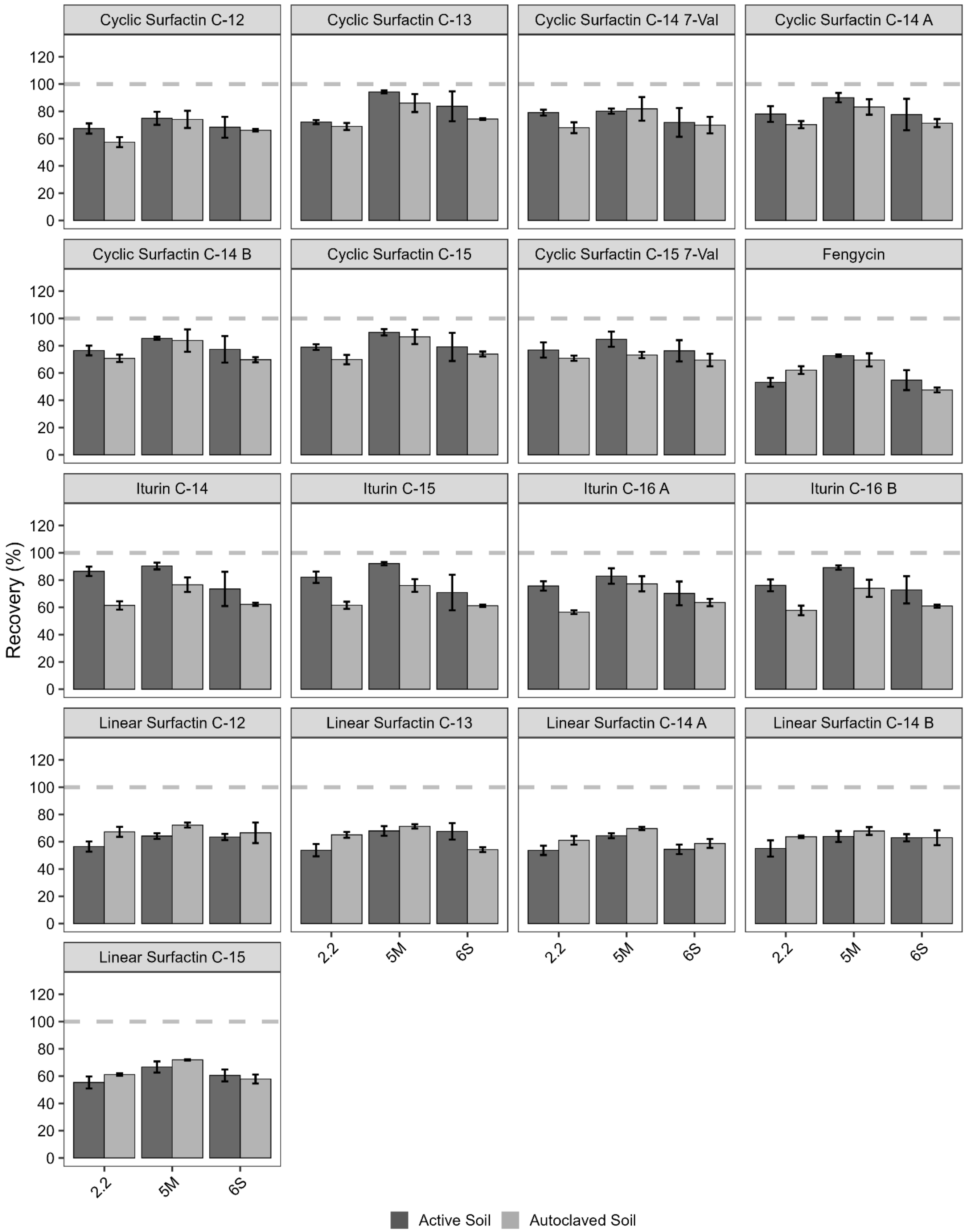

Figure SI 6: Lipopeptide recovery from the three different soils tested in this study. Data points and error bars represent mean ± standard deviation of triplicate (sacrificial) extractions. The grey horizontal dashed lines indicate 100% recovery.

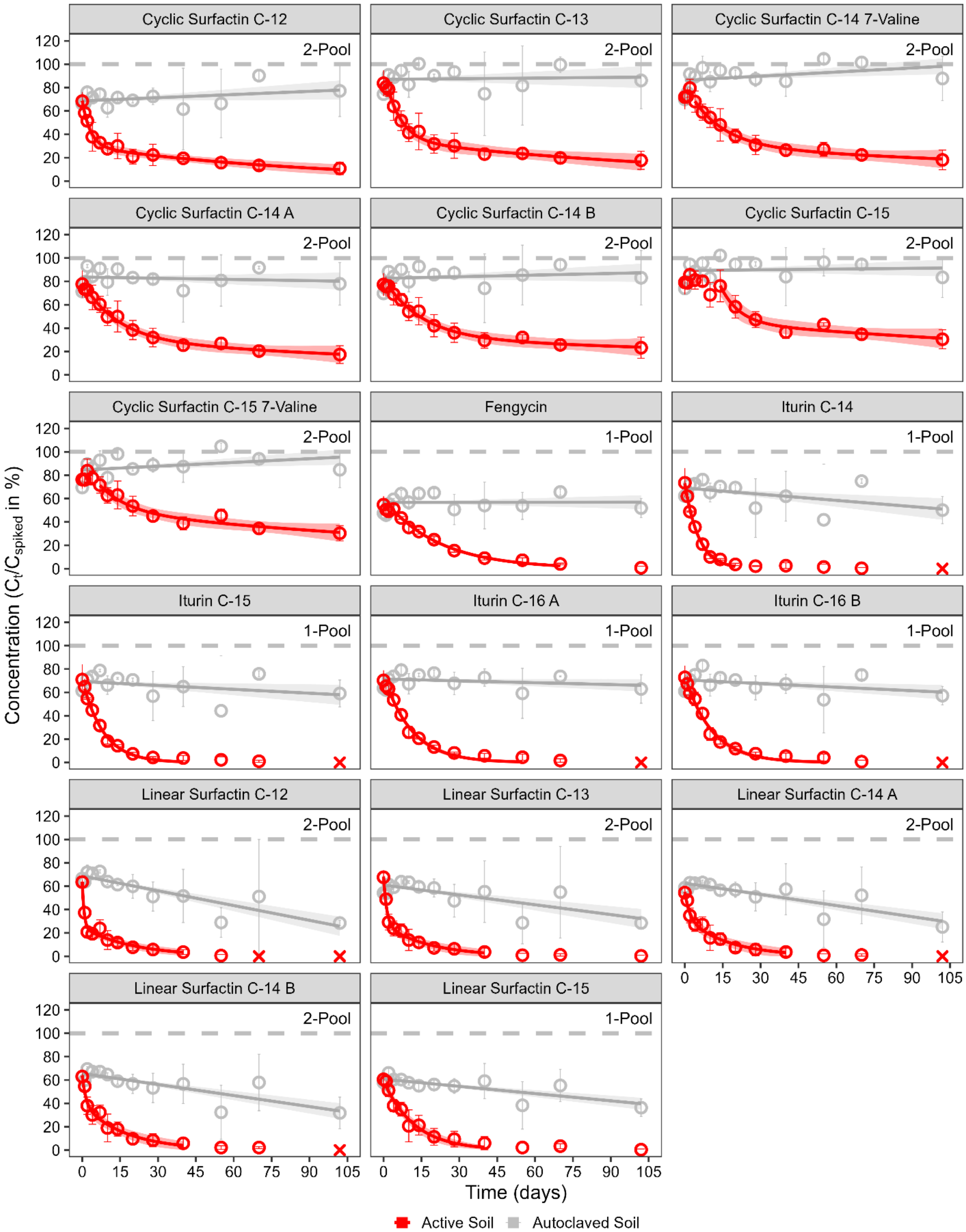

Figure SI 7: **A)** Concentration of lipopeptides during their incubation in soil 6S. “X” marks a value below the limit of quantification. The horizontal dashed line indicates the spiked concentration (grey). The red solid lines indicate the fitted model (i.e., 1-Pool or 2-Pool, provided as text in each panel) and the grey solid line indicates a linear fit of the concentrations detected in autoclaved-soil incubations. Shaded ribbons indicate the 95% confidence interval of the mean fit. Data points and error bars represent mean ± standard deviation of triplicate (sacrificial) extractions. The 2-pool model is used as an operational description of biphasic behavior and does not represent directly measurable sorbed and bioavailable fractions.

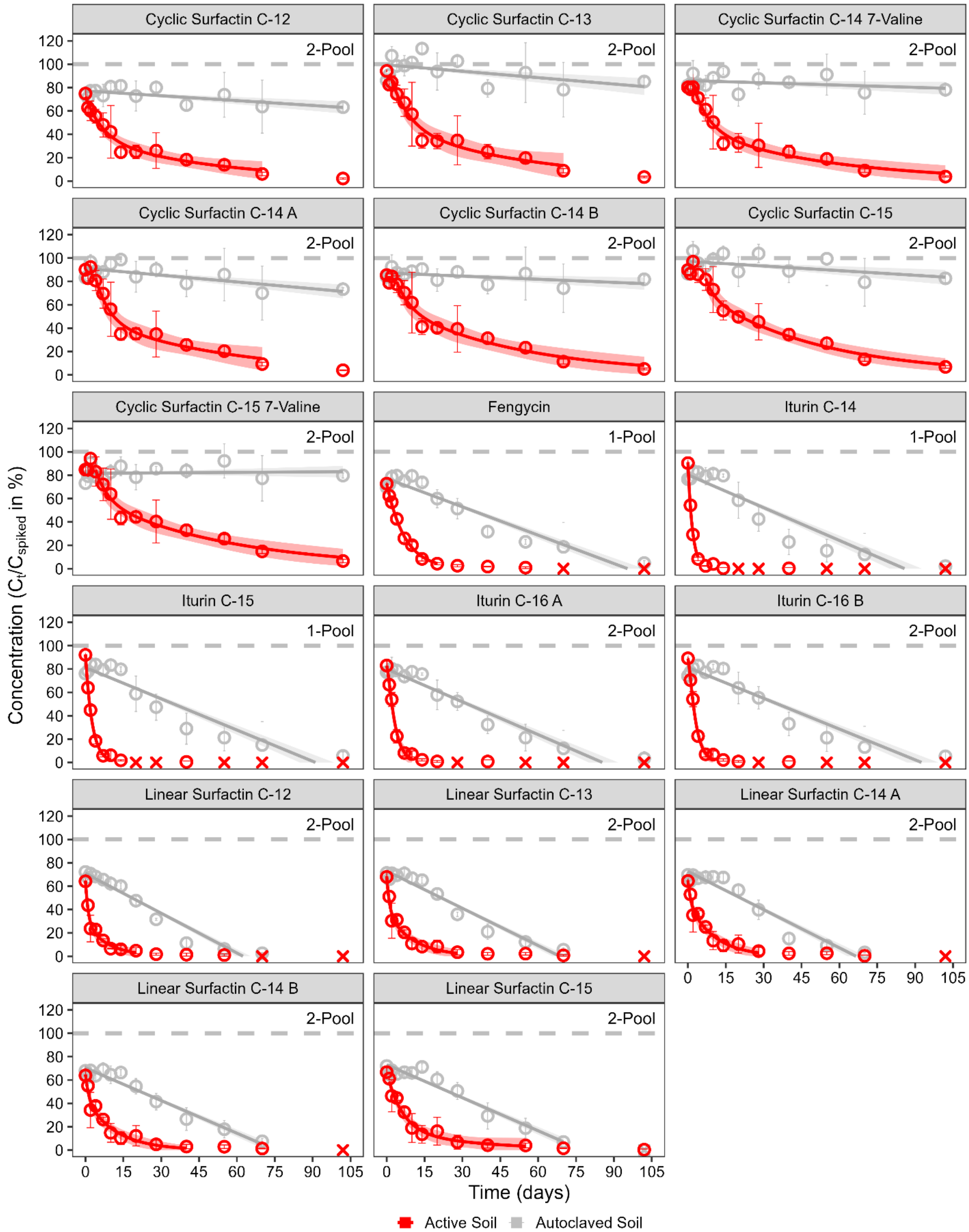

Figure SI 8: A) Concentration of lipopeptides during their incubation in soil 5M. "X" marks a value below the limit of quantification. The horizontal dashed line indicates the spiked concentration (grey). The red solid lines indicate the fitted model (i.e., 1-Pool or 2-Pool, provided as text in each panel) and the grey solid line indicates a linear fit of the concentrations detected in autoclaved-soil incubations. Shaded ribbons indicate the 95% confidence interval of the mean fit. Data points and error bars represent mean ± standard deviation of triplicate (sacrificial) extractions. The 2-pool model is used as an operational description of biphasic behavior and does not represent directly measurable sorbed and bioavailable fractions.

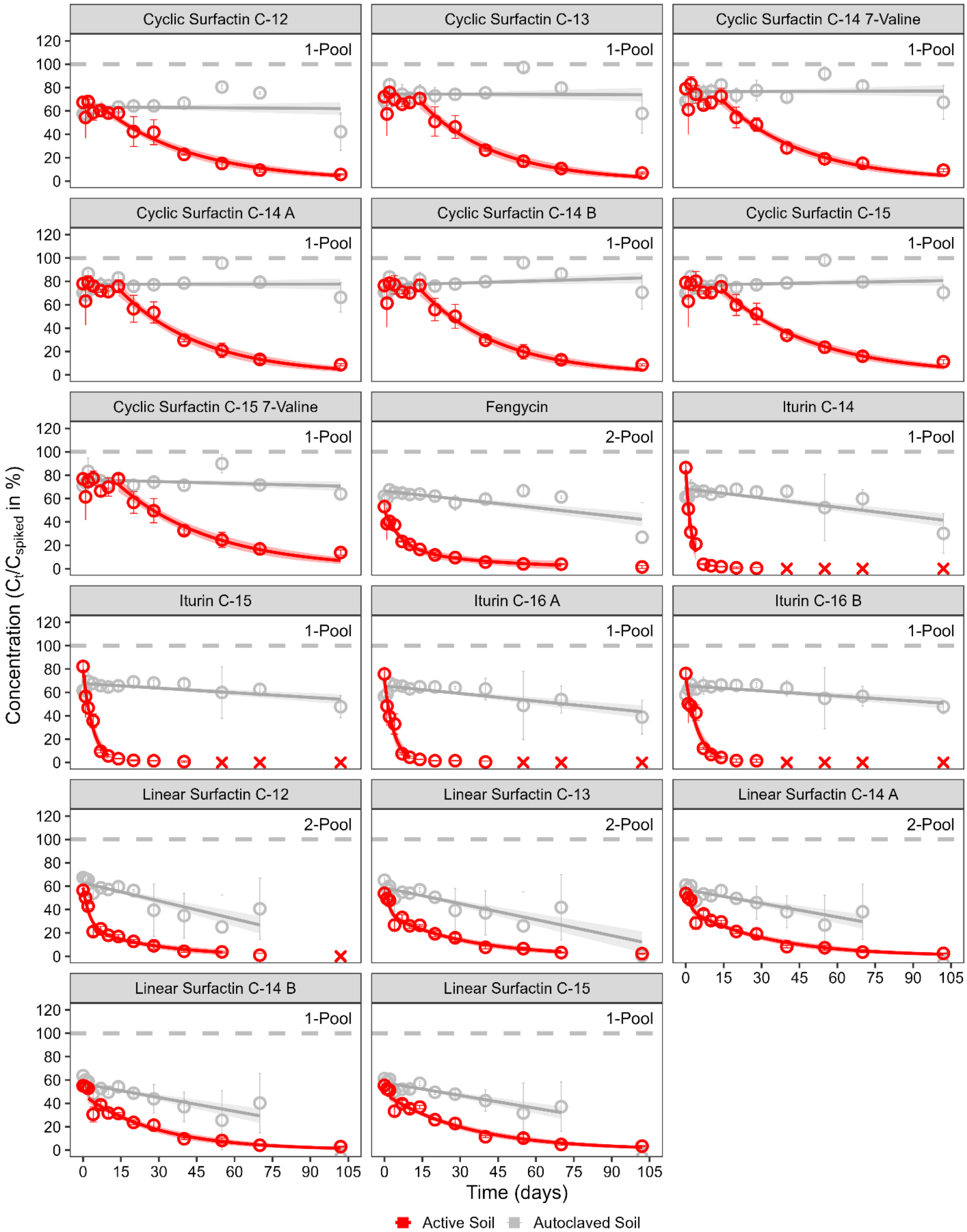

Figure SI 9: A) Concentration of lipopeptides during their incubation in soil 2.2. “X” marks a value below the limit of quantification. The horizontal dashed line indicates the spiked concentration (grey). The red solid lines indicate the fitted model (i.e., 1-Pool or 2-Pool, provided as text in each panel) and the grey solid line indicates a linear fit of the concentrations detected in autoclaved-soil incubations. Shaded ribbons indicate the 95% confidence interval of the mean fit. Data points and error bars represent mean ± standard deviation of triplicate (sacrificial) extractions. The 2-pool model is used as an operational description of biphasic behavior and does not represent directly measurable sorbed and bioavailable fractions.

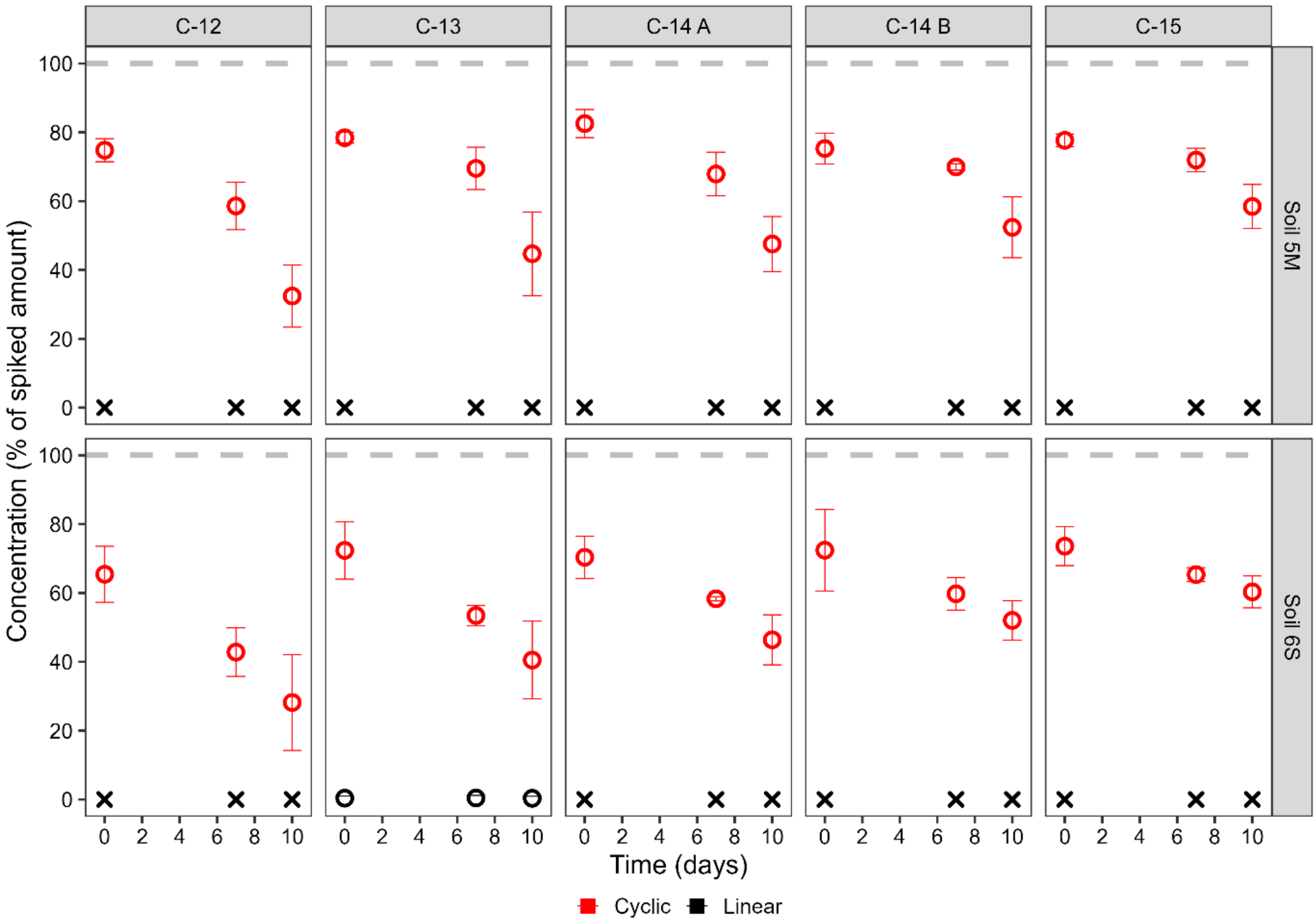

Figure SI 10: Concentration of cyclic surfactins and linearized variants during soil incubation in which only cyclic surfactins were spiked. "X" marks a value below the limit of quantification. The horizontal dashed line indicates the spiked concentration of cyclic surfactin (grey). Data points and error bars represent mean ± standard deviation of triplicate (sacrificial) extractions.

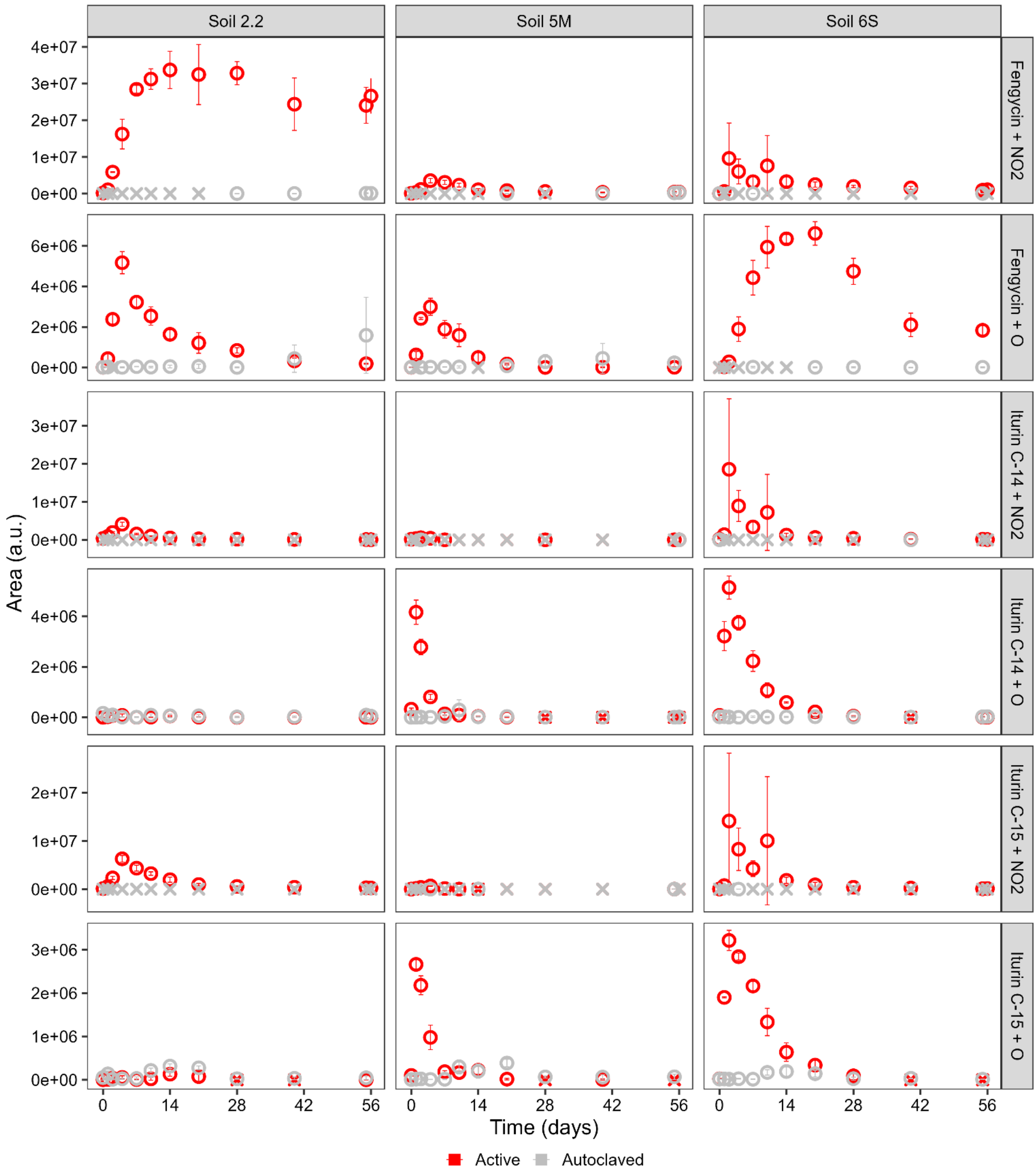

Figure SI 11: Peak area of transformation products assigned to iturins and fengycins during soil incubations. "X" marks a value below the limit of detection. Data points and error bars represent mean ± standard deviation of triplicate (sacrificial) extractions.

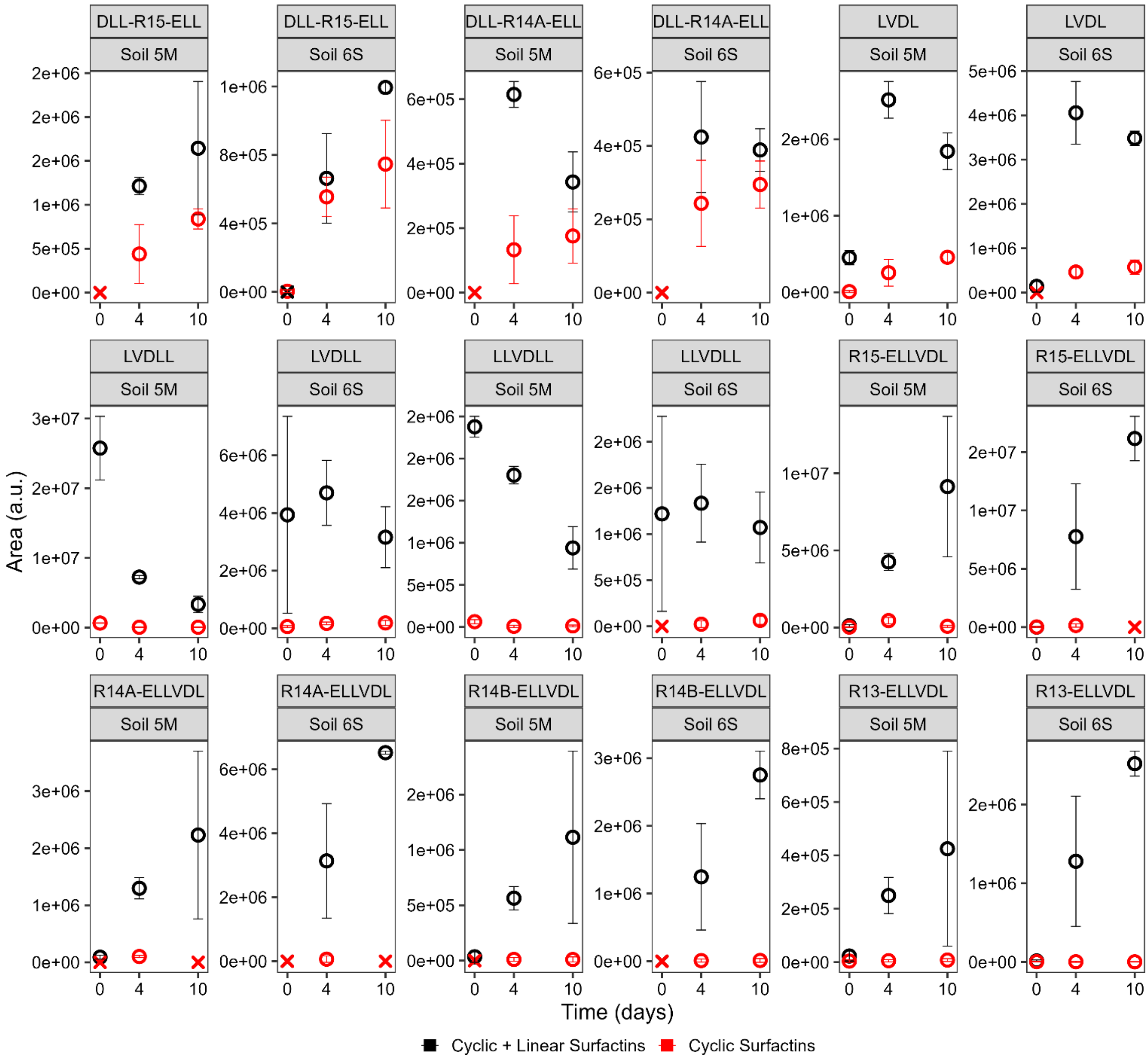

Figure SI 12: Peak area of transformation products assigned to cyclic and linear surfactins during soil incubations where either both cyclic and linear or only cyclic surfactins were spiked. "X" marks a value below the limit of detection. Data points and error bars represent mean ± standard deviation of triplicate (sacrificial) extractions. Peptides are described using the 1-letter code for amino acid residues (with R# indicating the length of the alkyl chain).

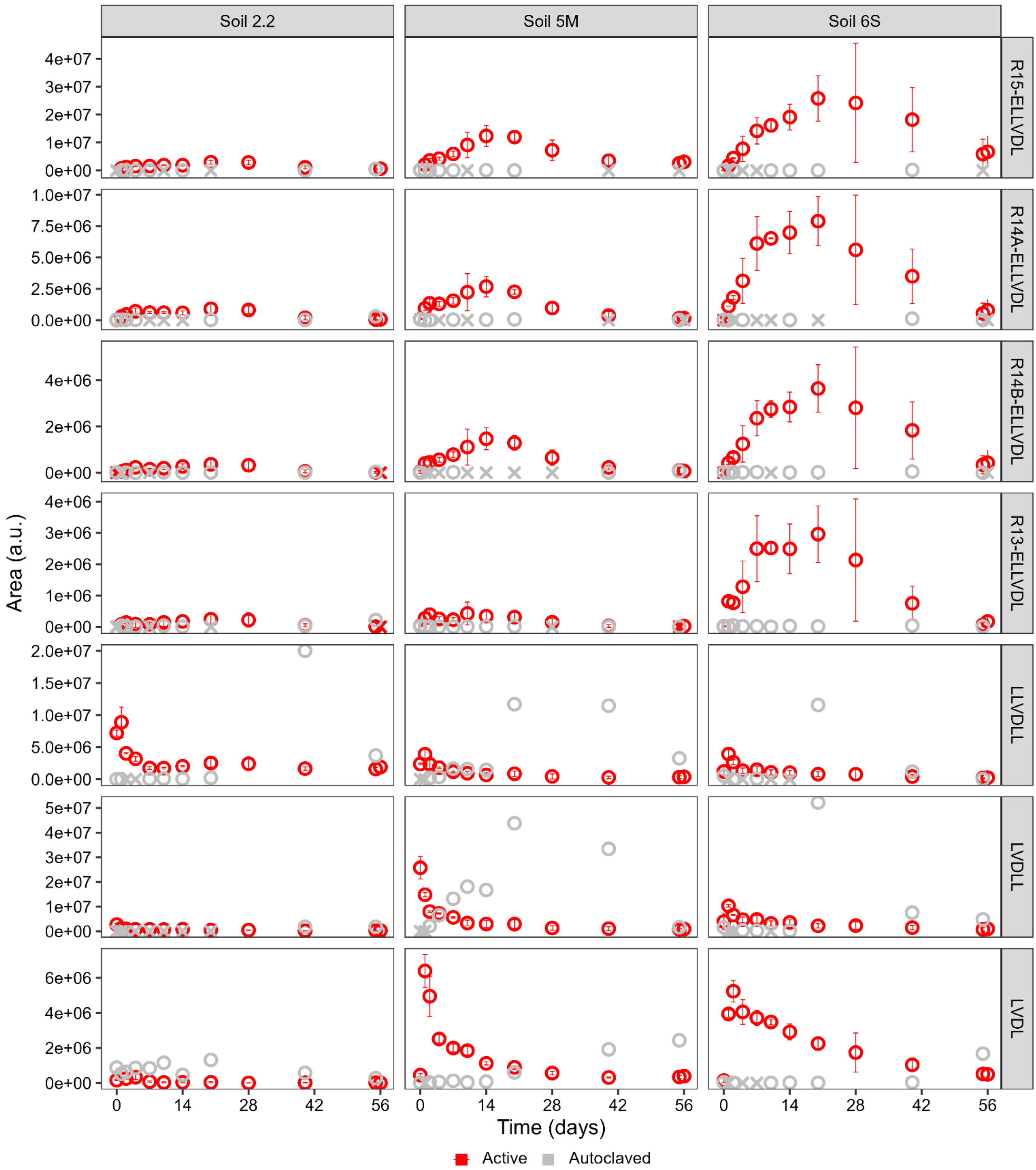

Figure SI 13: Peak area of transformation products predominantly assigned to linear surfactin during soil incubations. “X” marks a value below the limit of detection. Data points and error bars represent mean ± standard deviation of triplicate (sacrificial) extractions. Peptides are described using the 1-letter code for amino acid residues (with R# indicating the length of the alkyl chain).

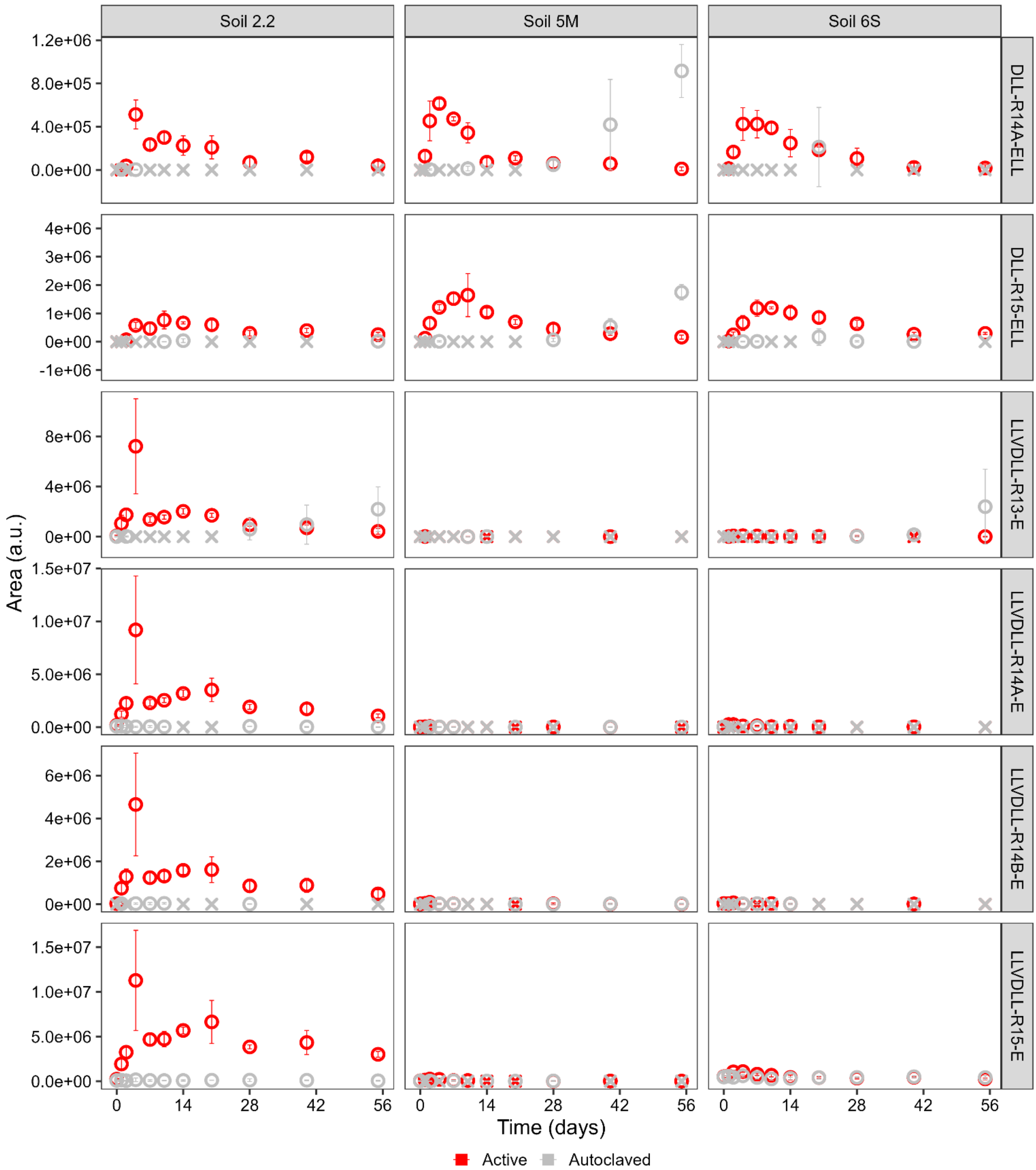

Figure SI 14: Peak area of transformation products predominantly assigned to cyclic surfactin during soil incubations. "X" marks a value below the limit of detection. Data points and error bars represent mean ± standard deviation of triplicate (sacrificial) extractions. Peptides are described using the 1-letter code for amino acid residues (with R# indicating the length of the alkyl chain).

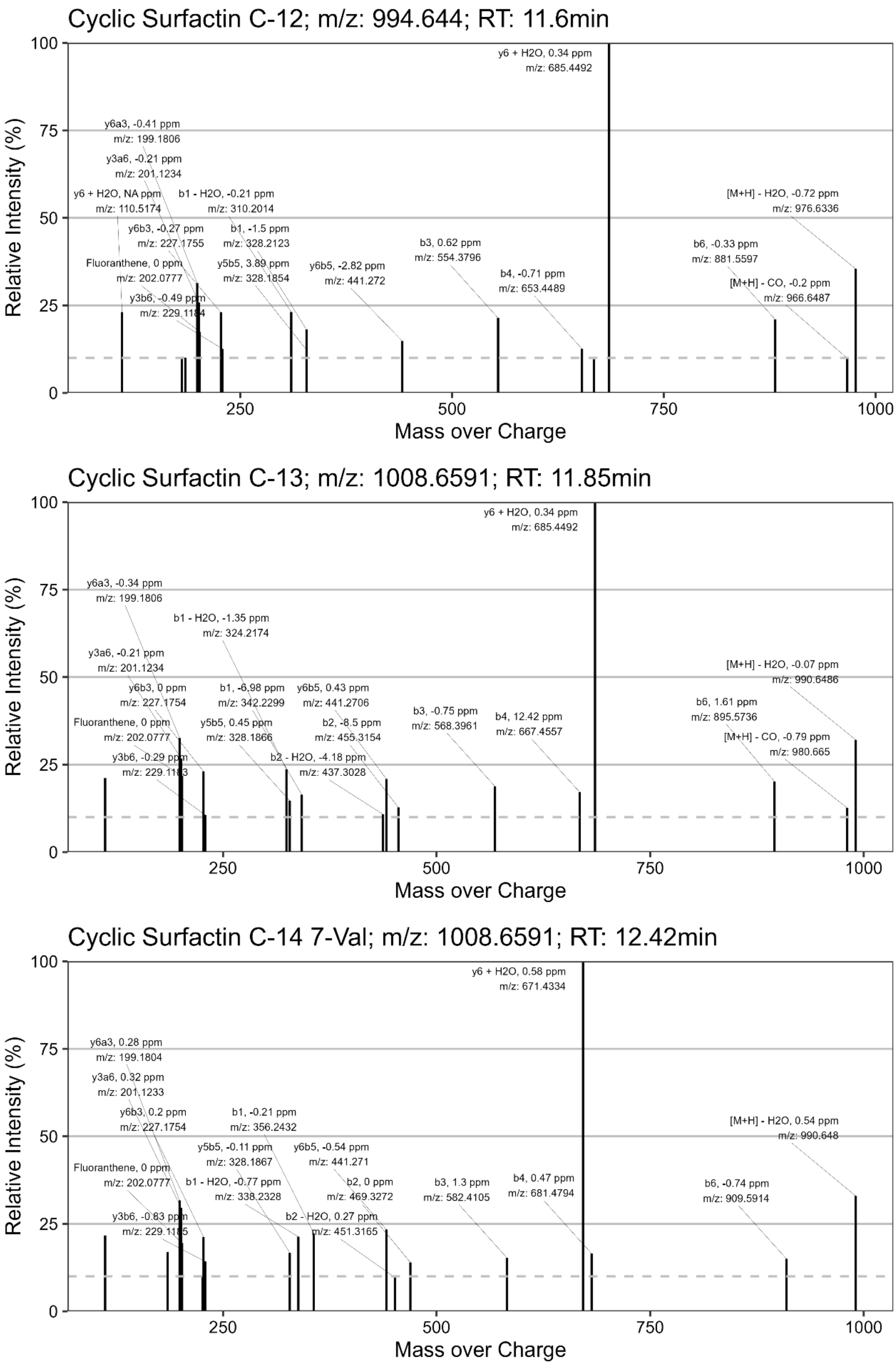

Figure SI 15: MS/MS Spectra of investigated lipopeptides with fragments annotated in Roepstorff-Fohlmann-Biemann format. For improved readability, the precursor was removed from the spectra. Remaining peaks were normalized to the most intense peak, and peaks smaller than 10% were removed (dashed horizontal grey line).

Cyclic Surfactin C-14 A; m/z: 1022.6747; RT: 12.35min

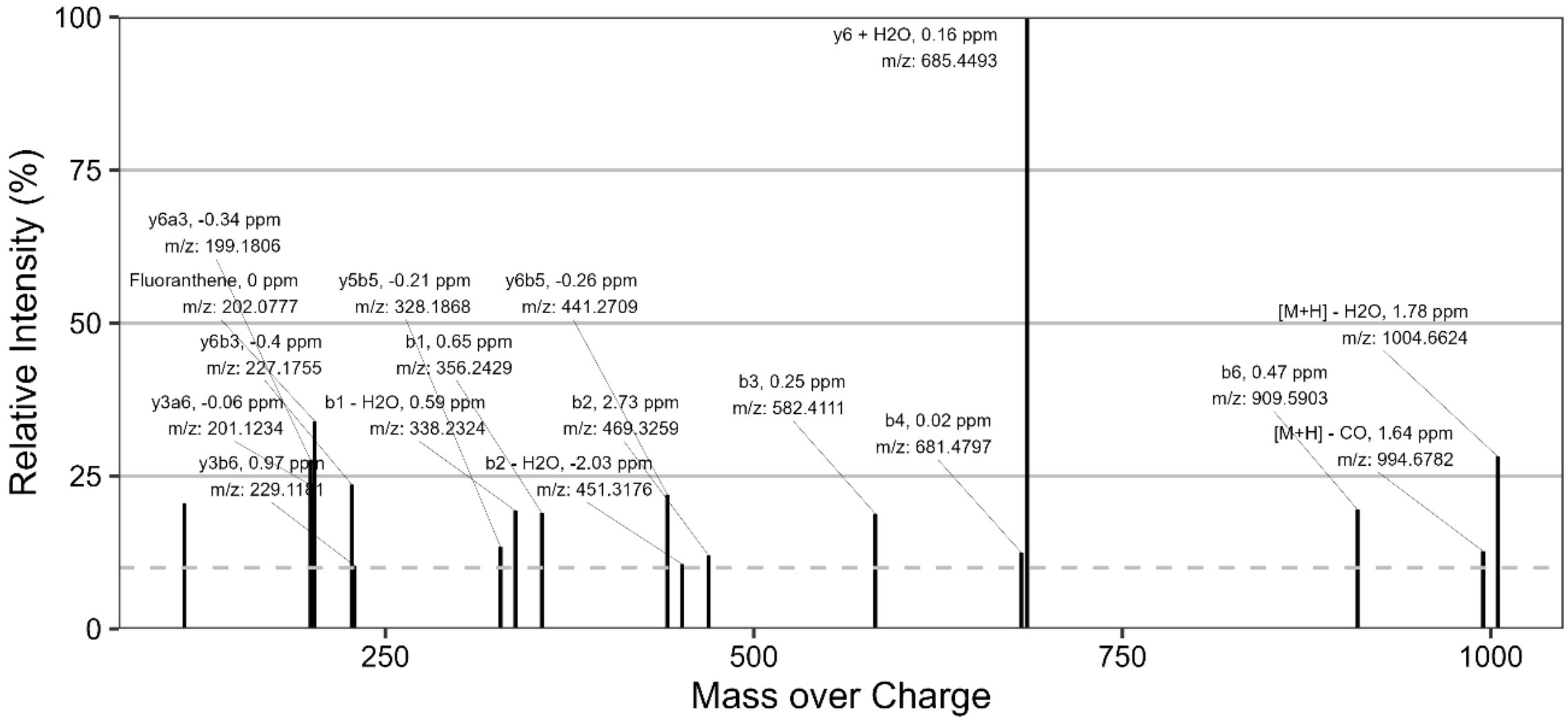

Cyclic Surfactin C-14 B; m/z: 1022.6747; RT: 12.22min

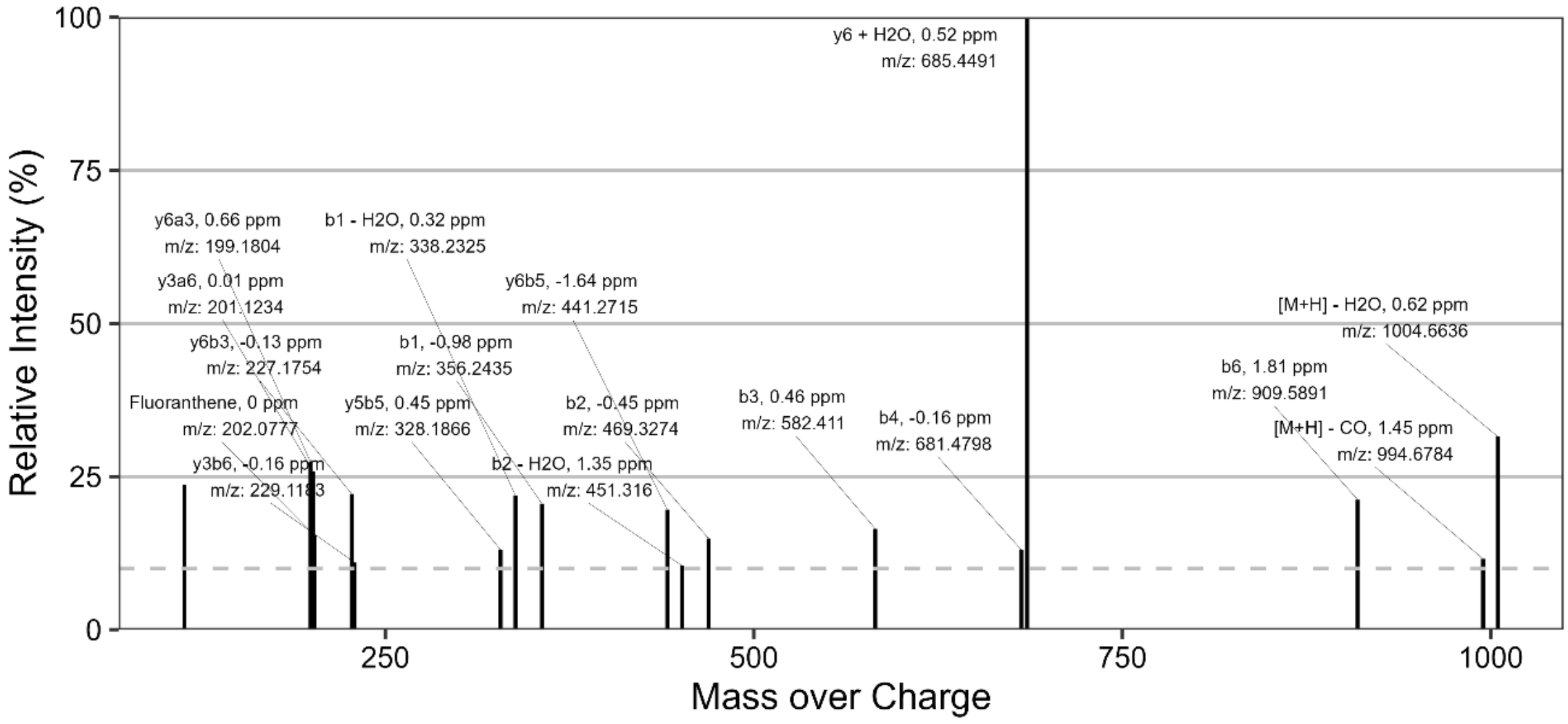

Cyclic Surfactin C-15 7-Val; m/z: 1022.6747; RT: 12.76min

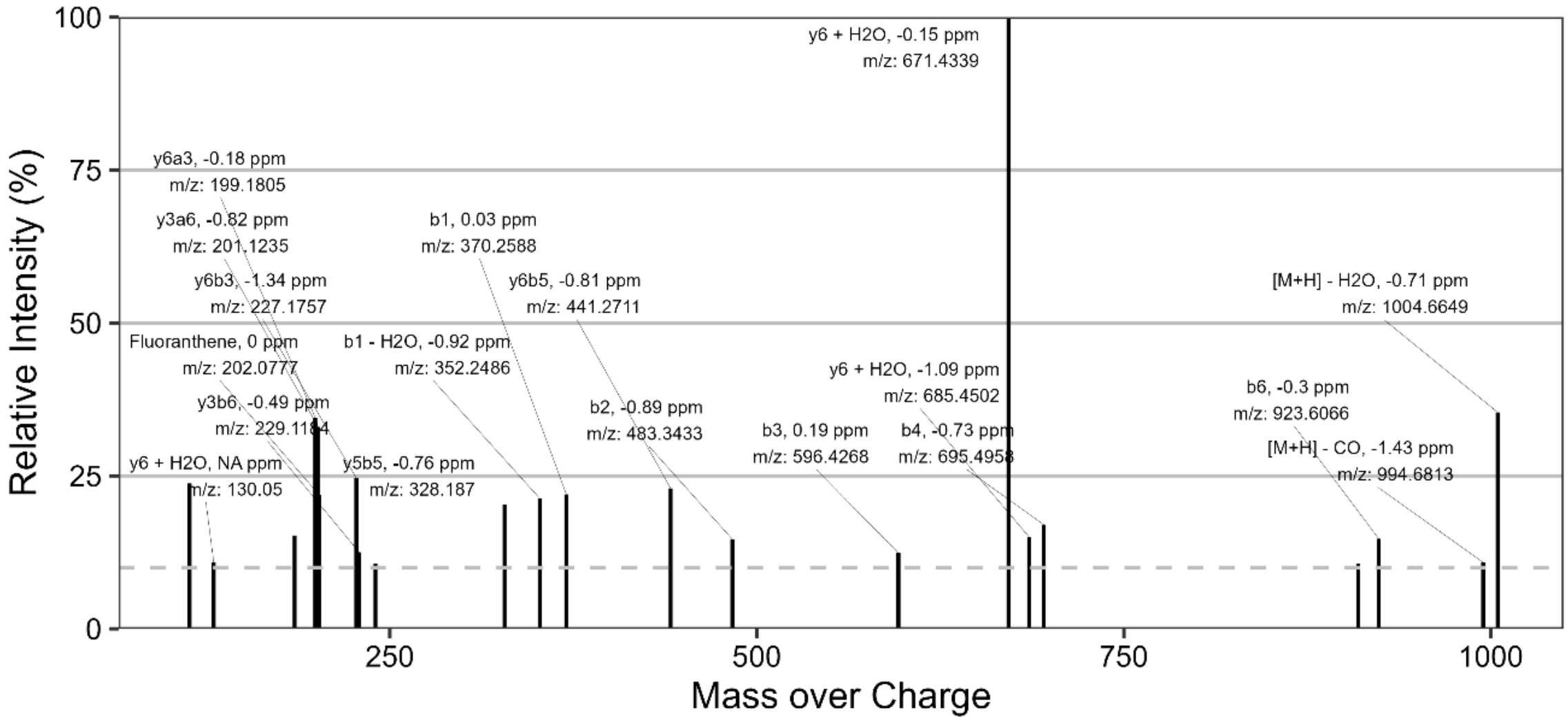

Figure SI 15 (continued)

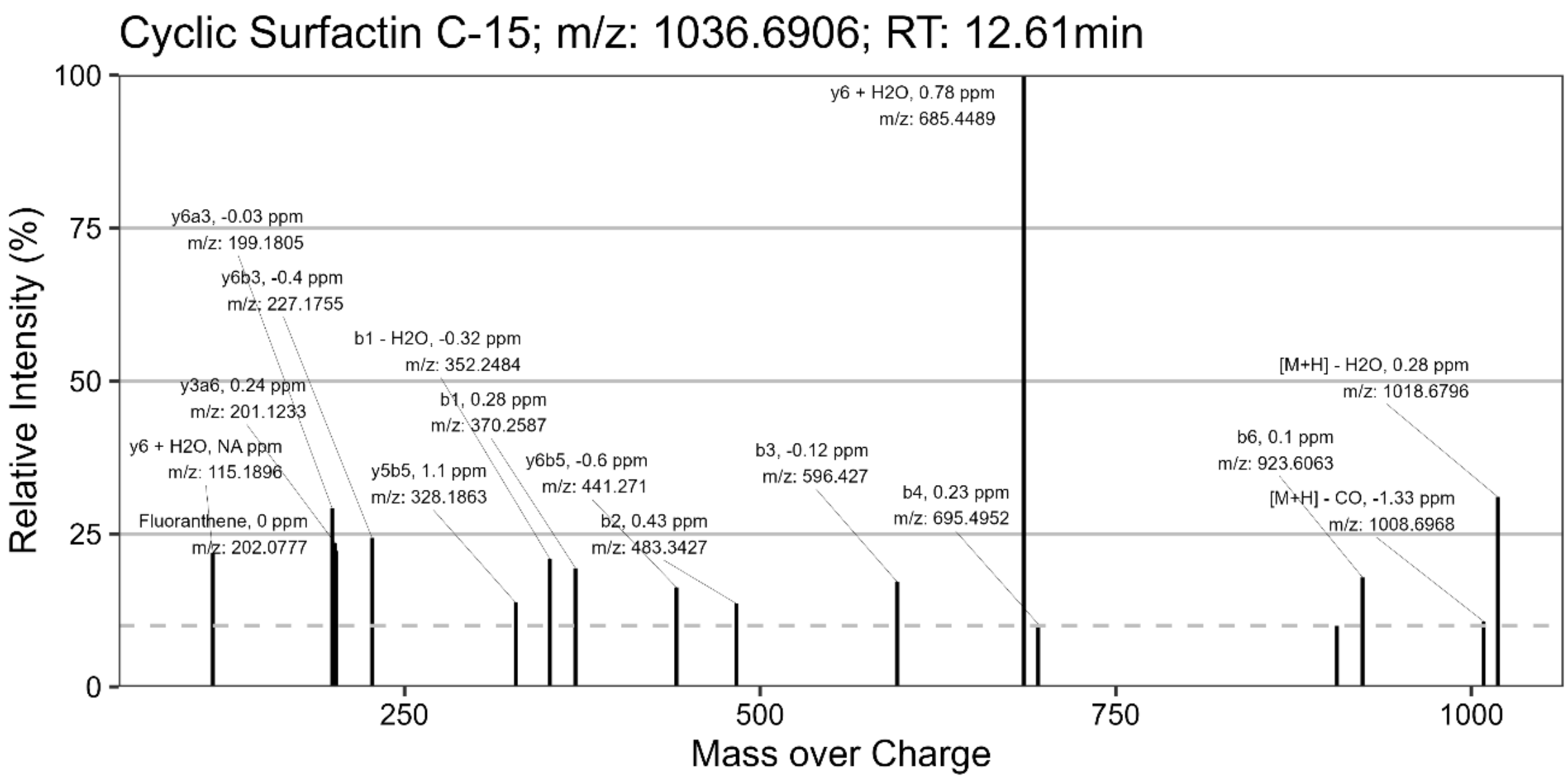

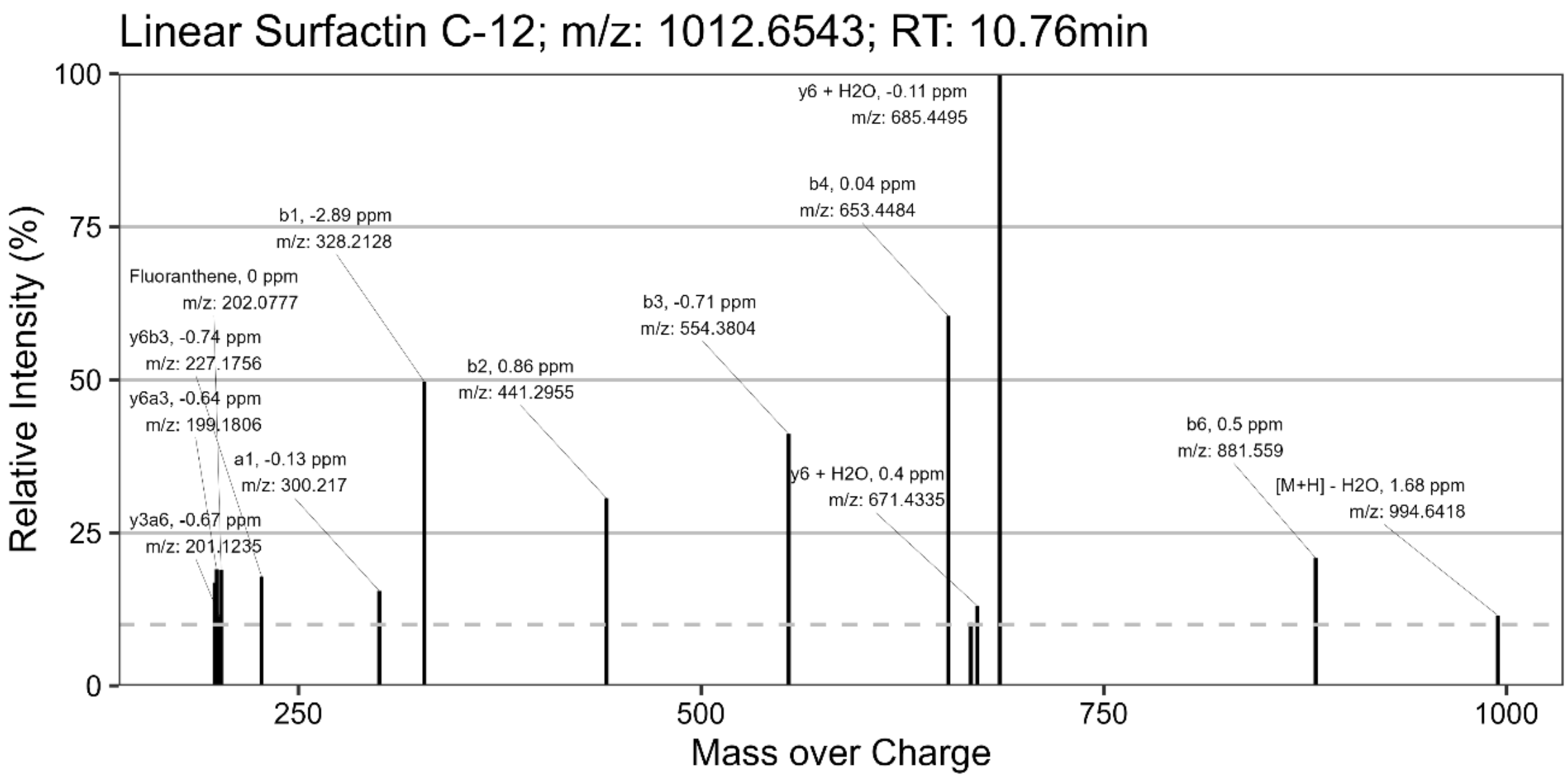

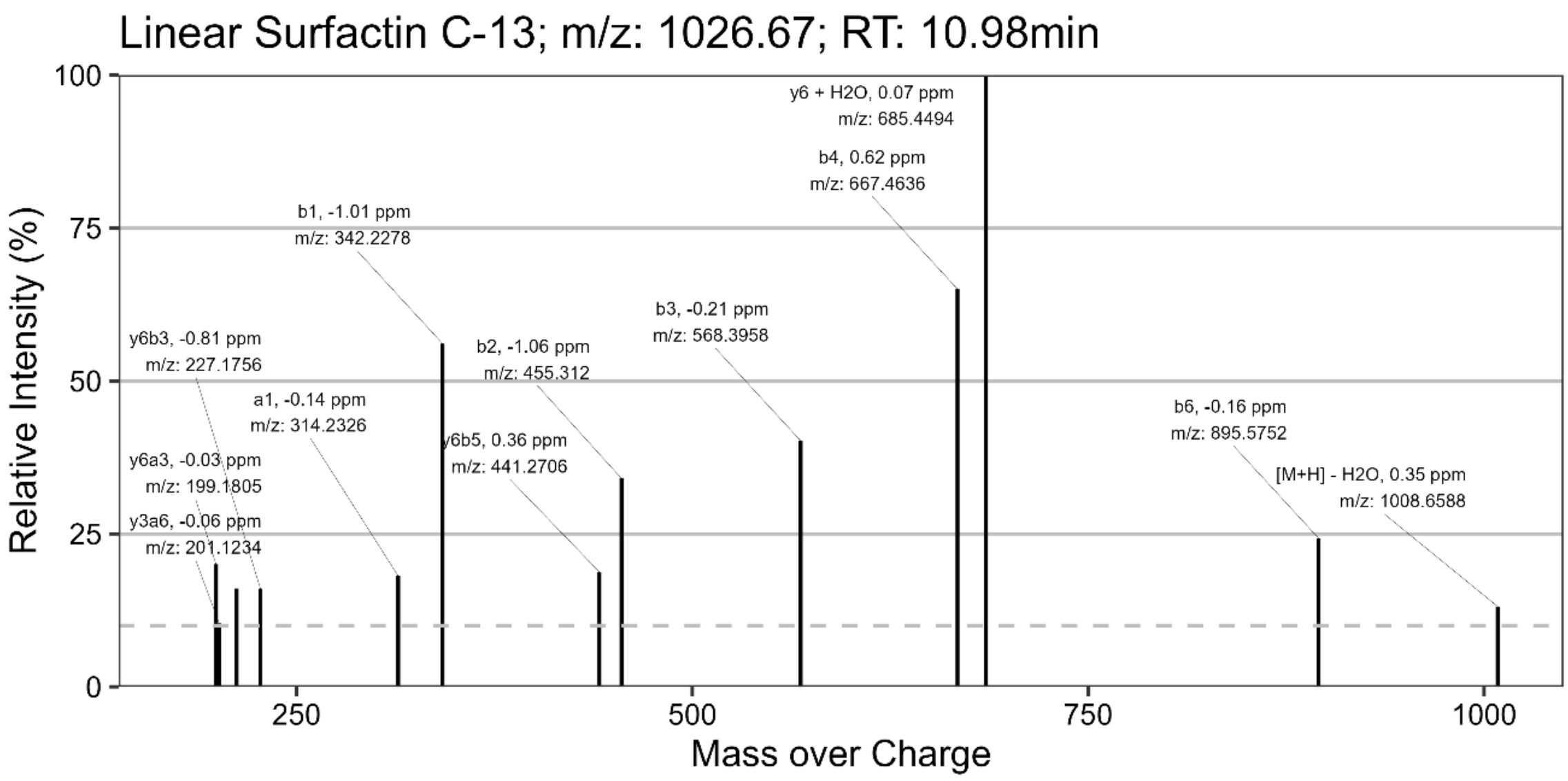

Figure SI 15 (continued)

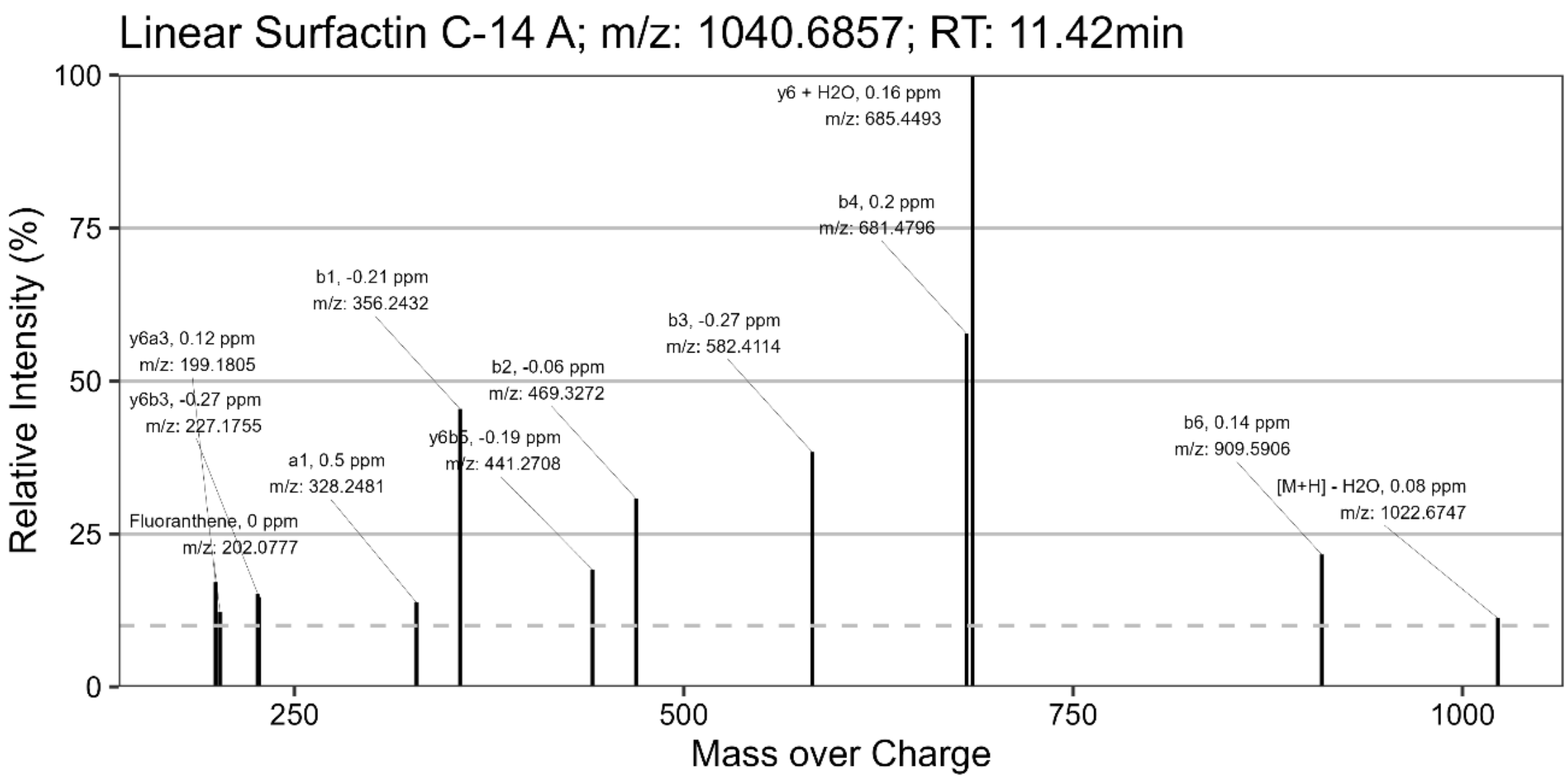

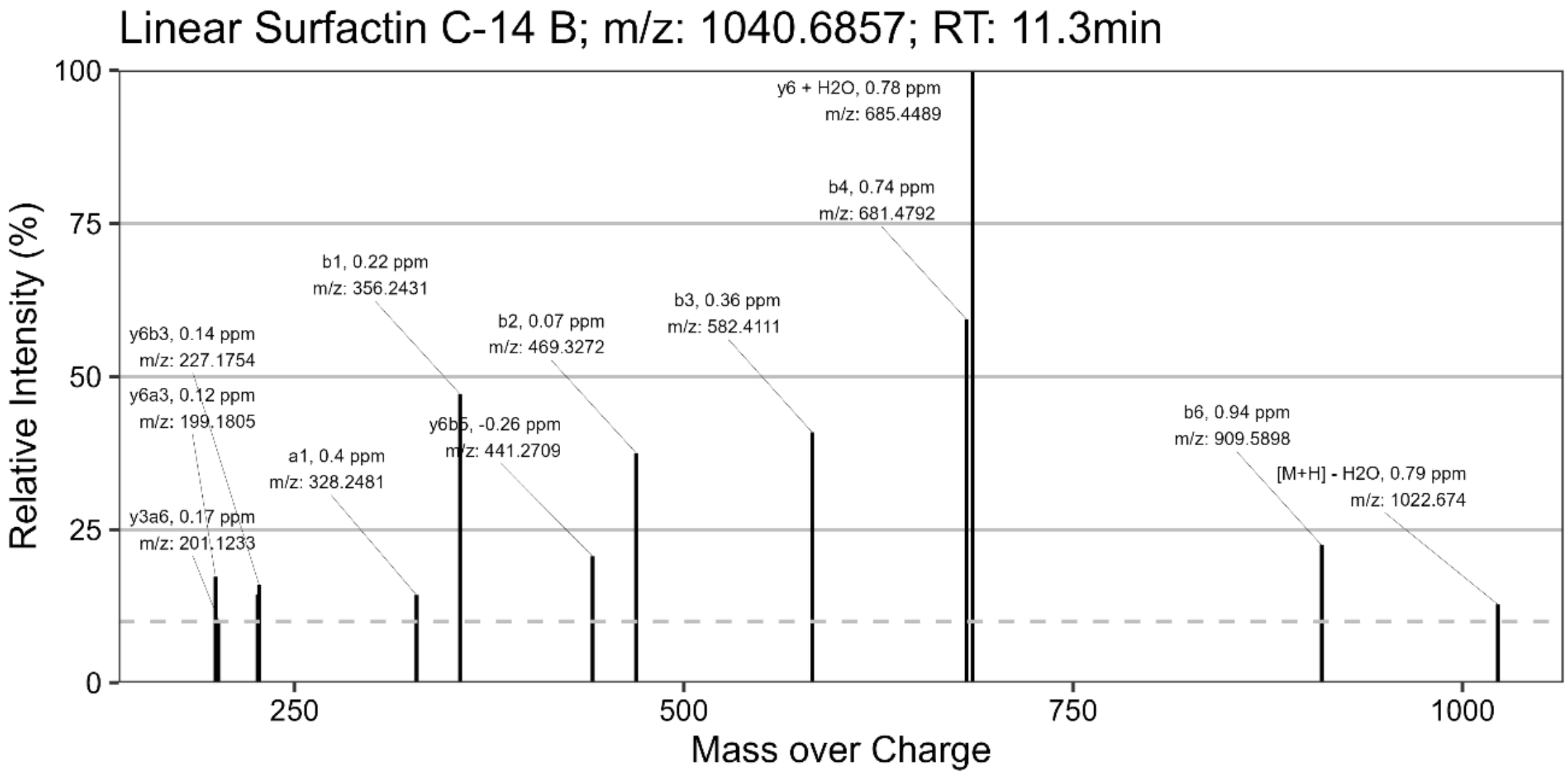

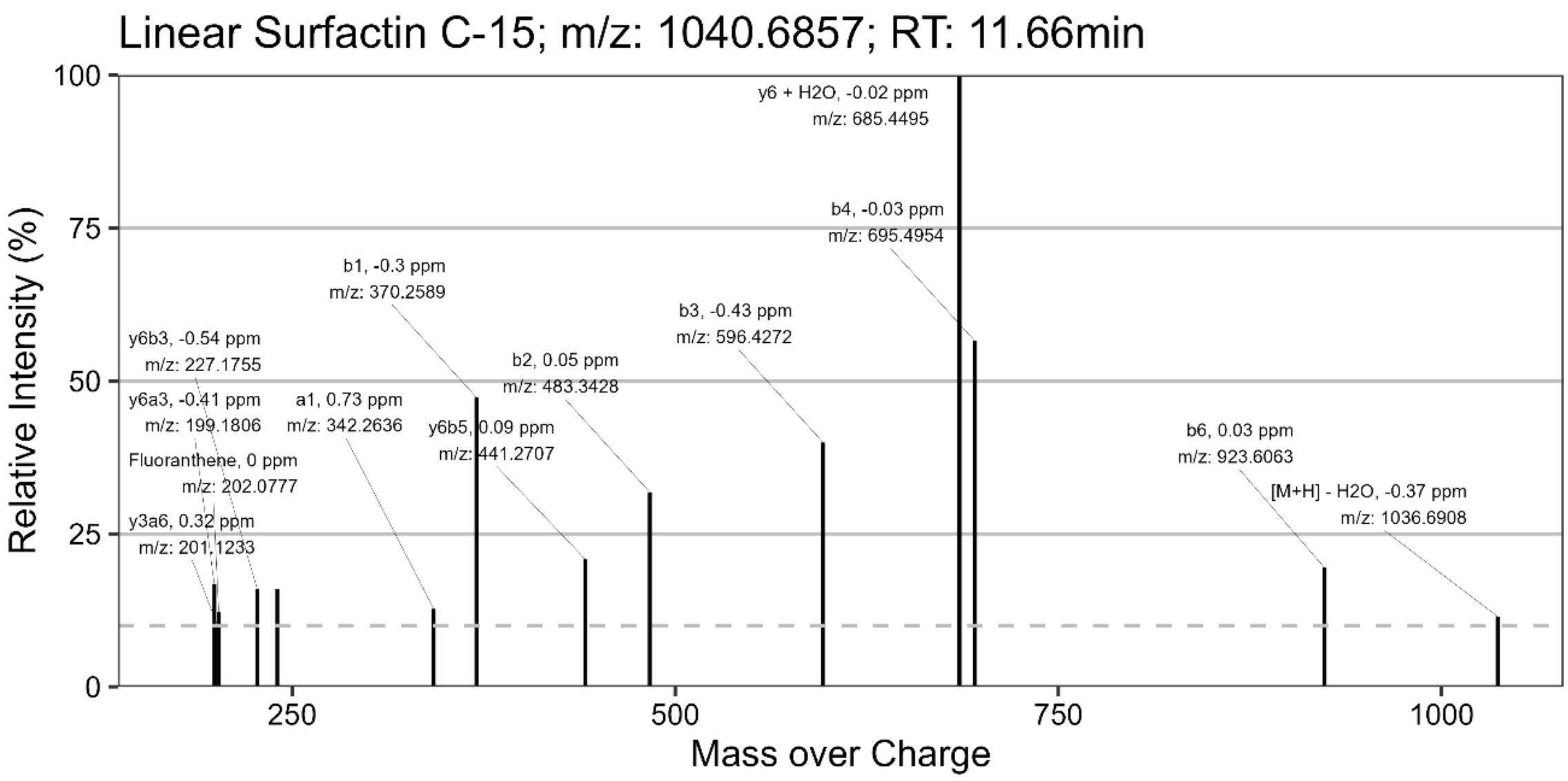

Figure SI 15 (continued)

Iturin C-14; m/z: 1043.5523; RT: 7.79min

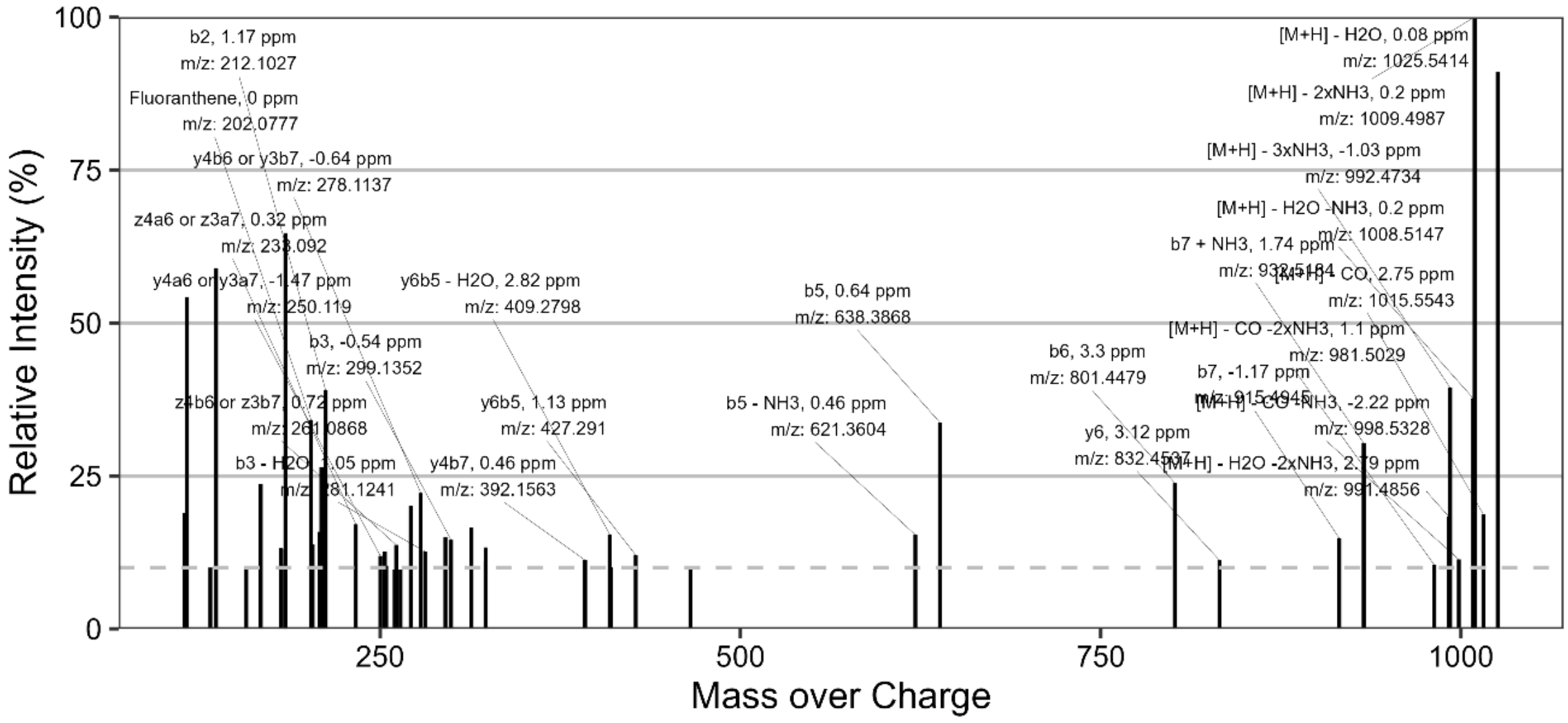

Iturin C-15; m/z: 1057.56794; RT: 8.11min

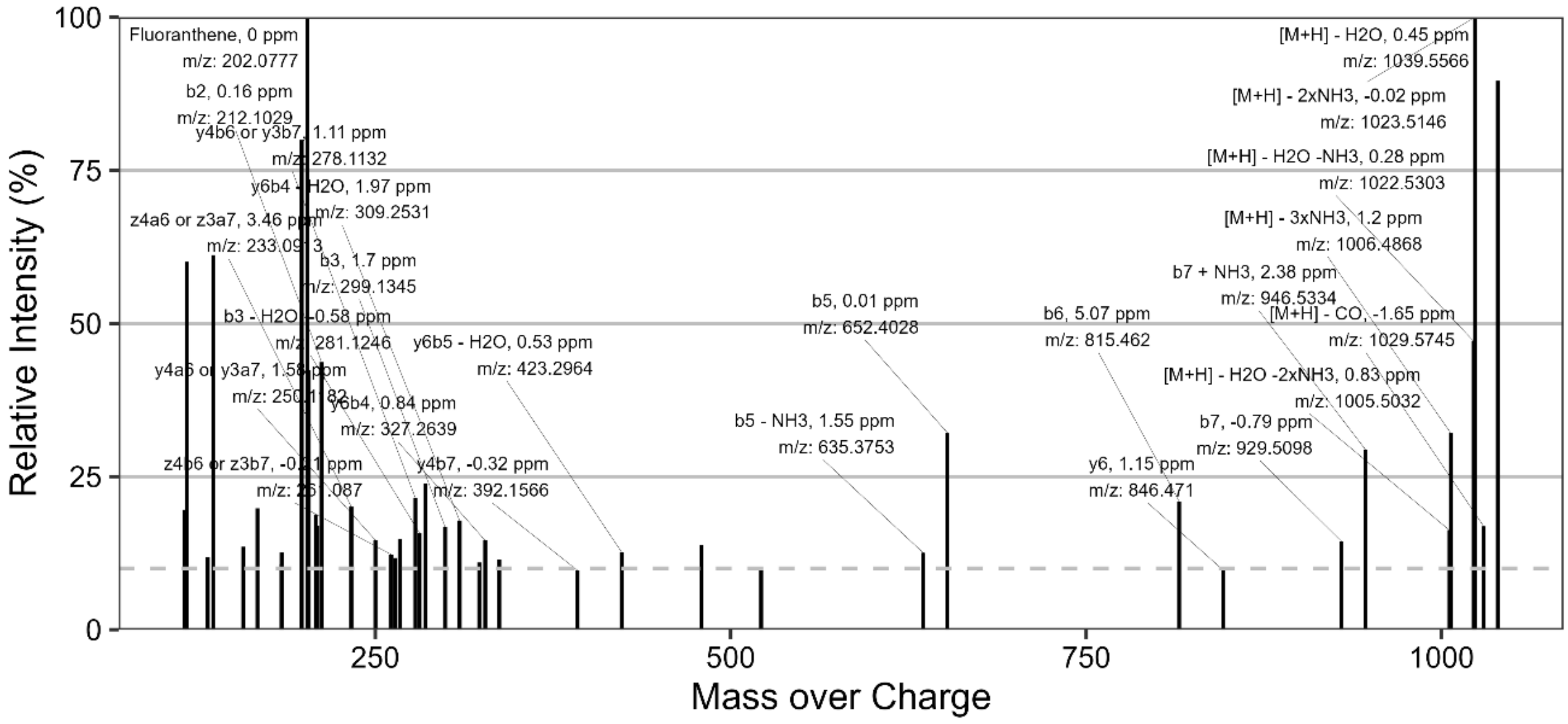

Iturin C-15; m/z: 1057.56794; RT: 8.03min

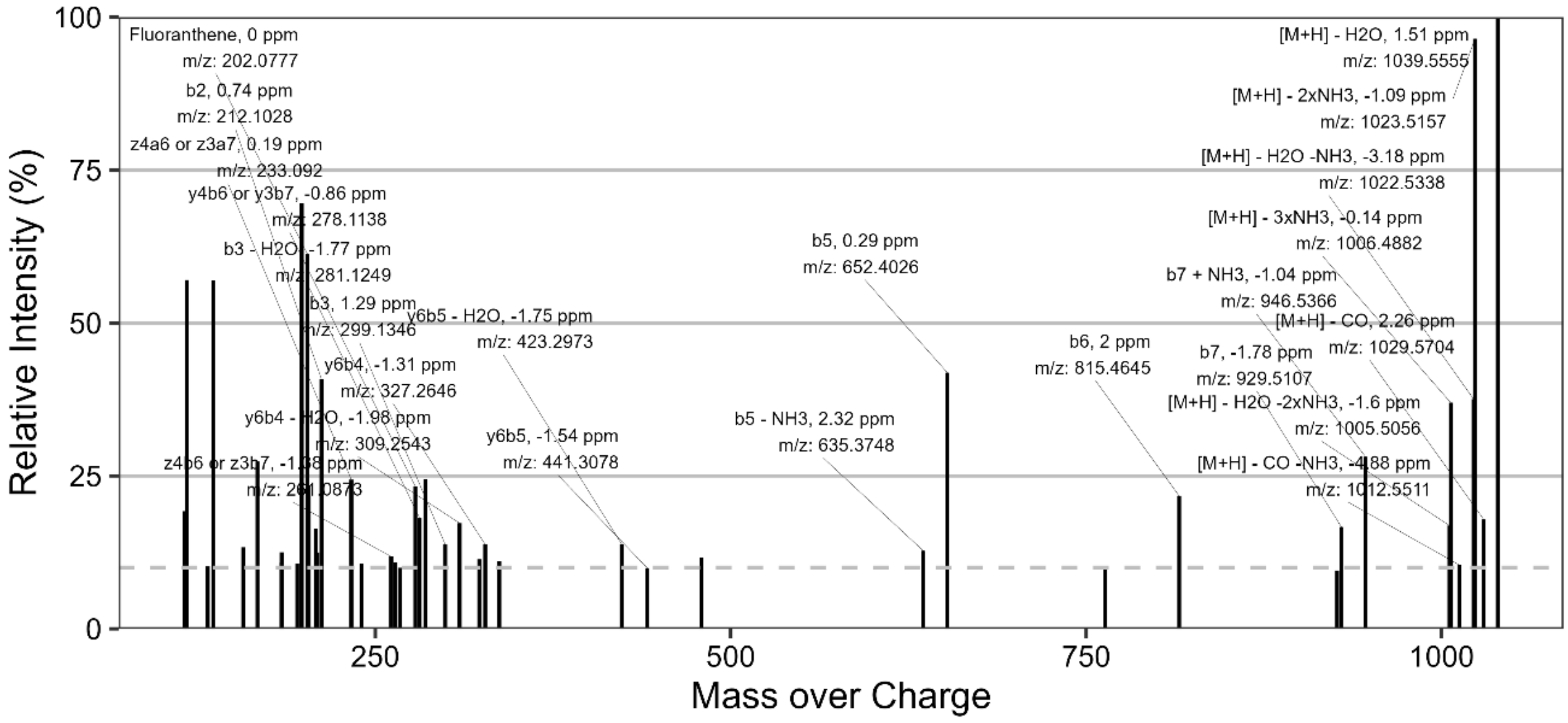

Figure SI 15 (continued)

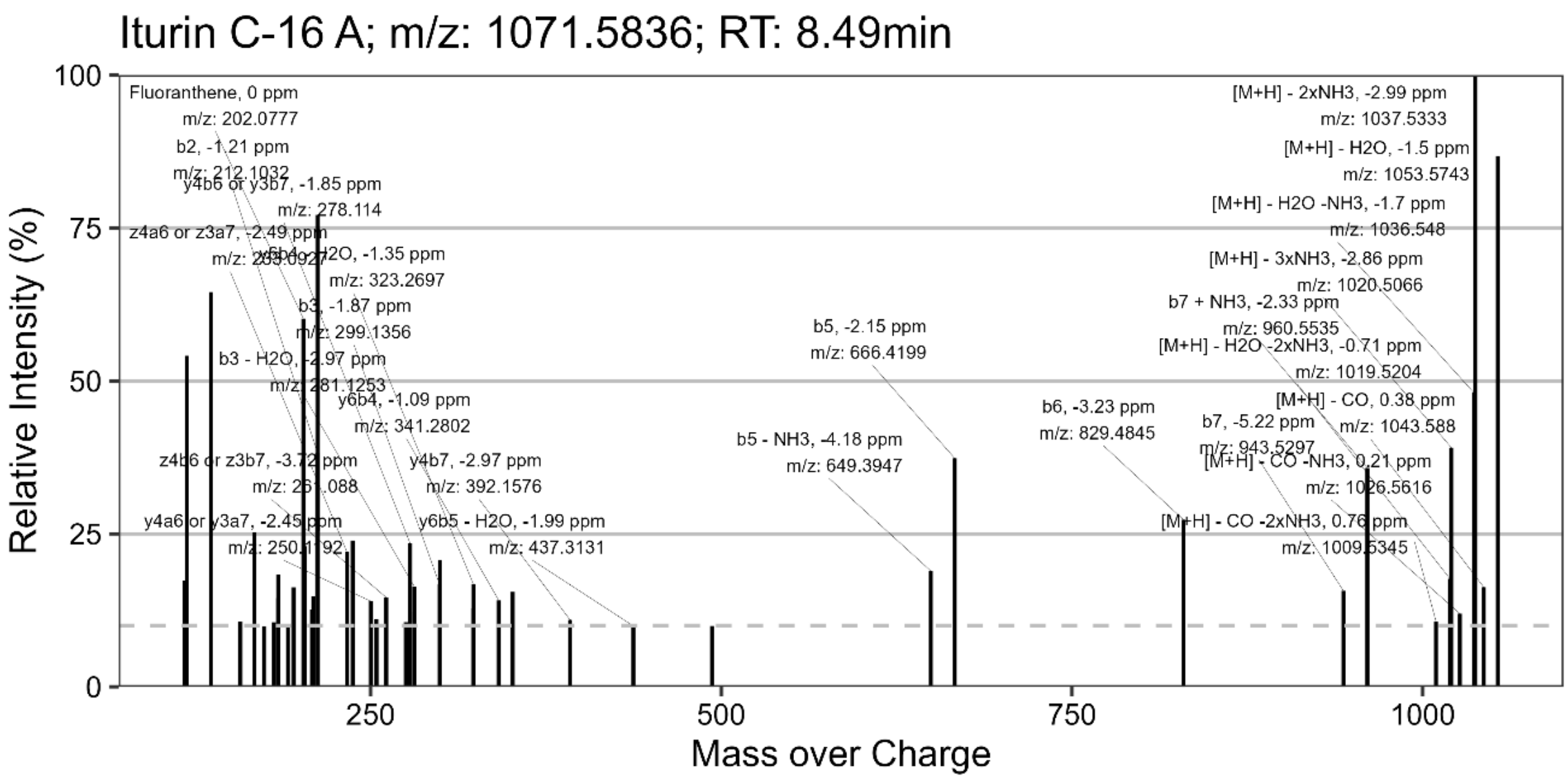

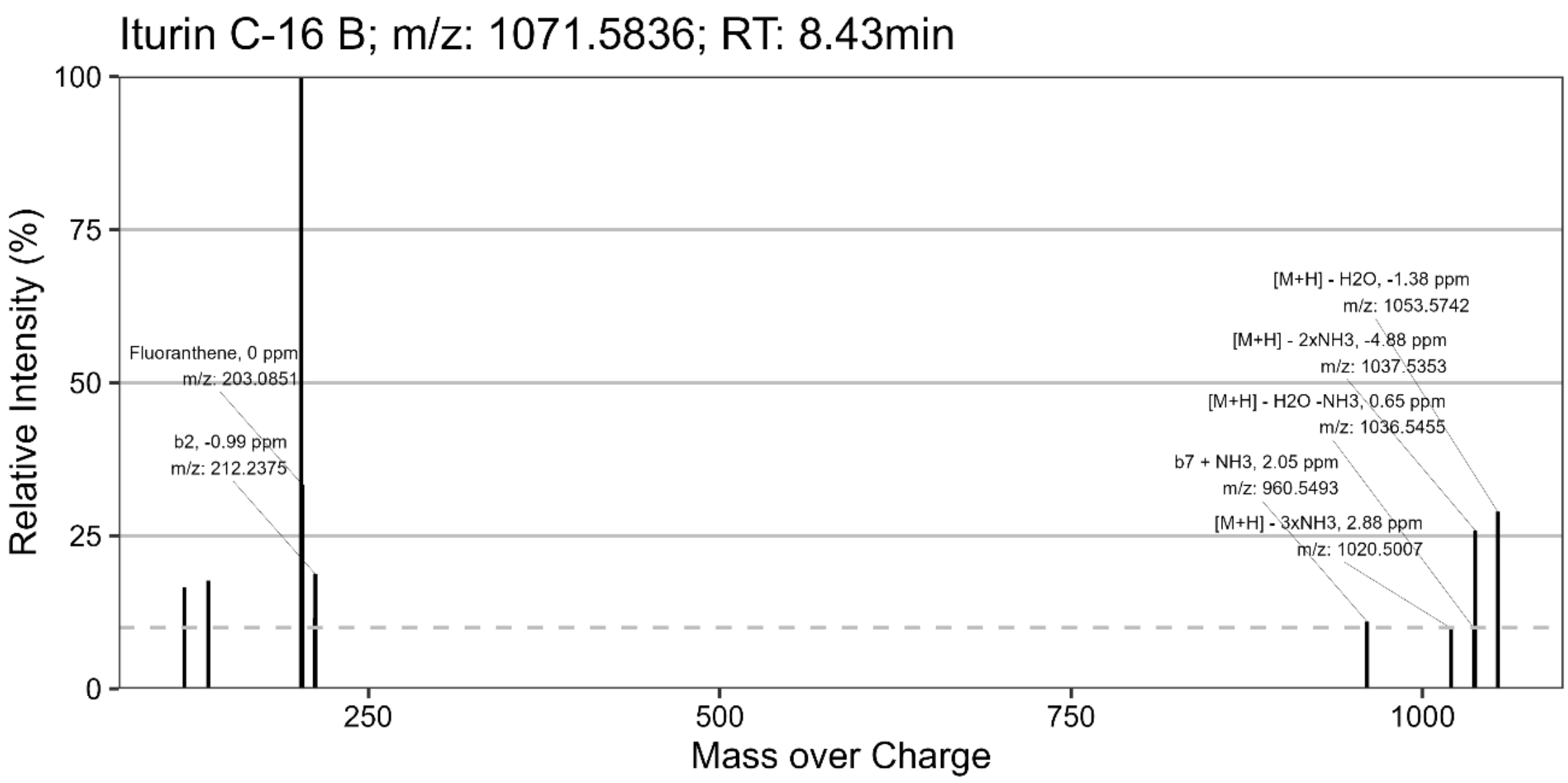

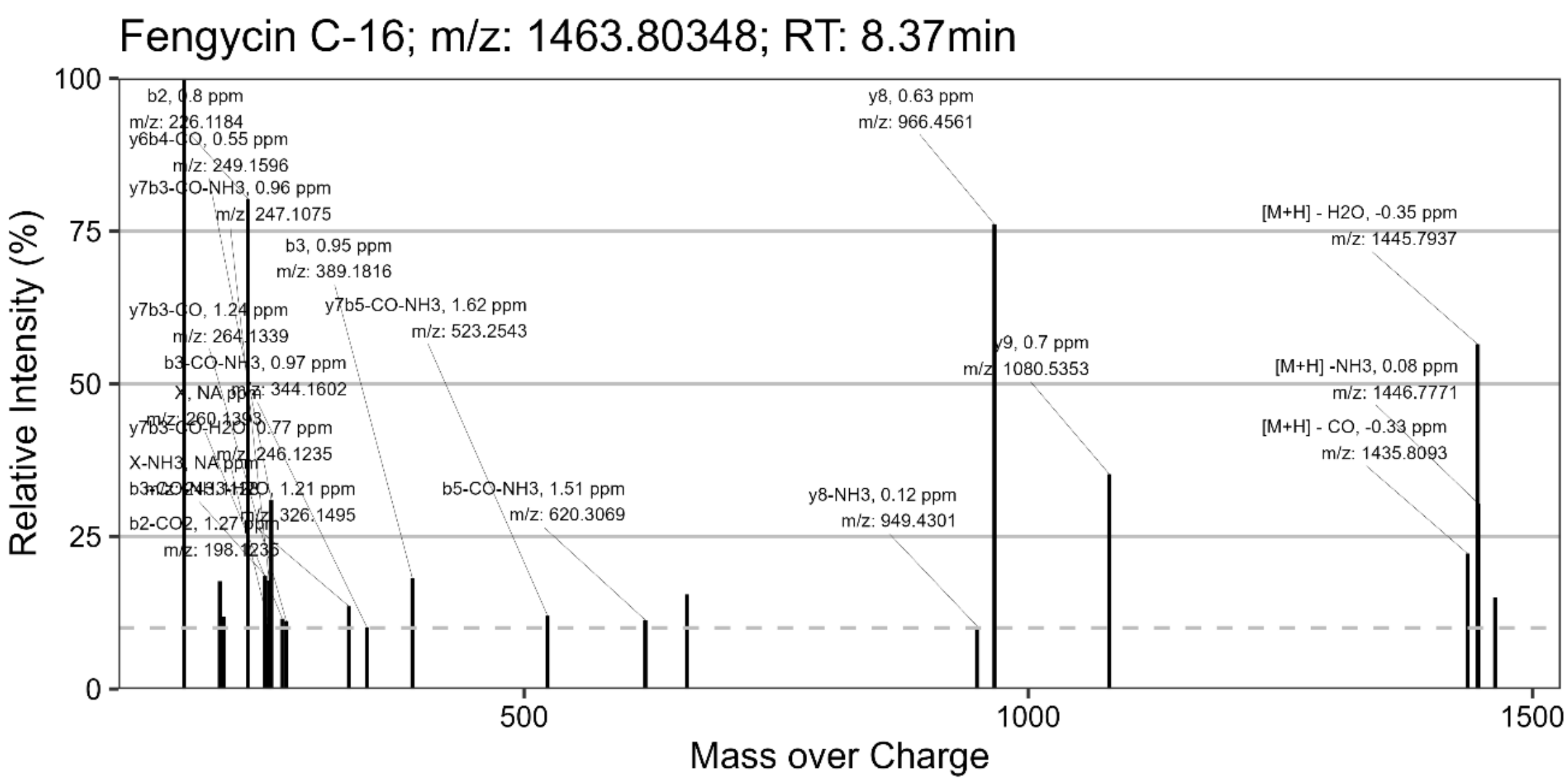

Figure SI 15 (continued)

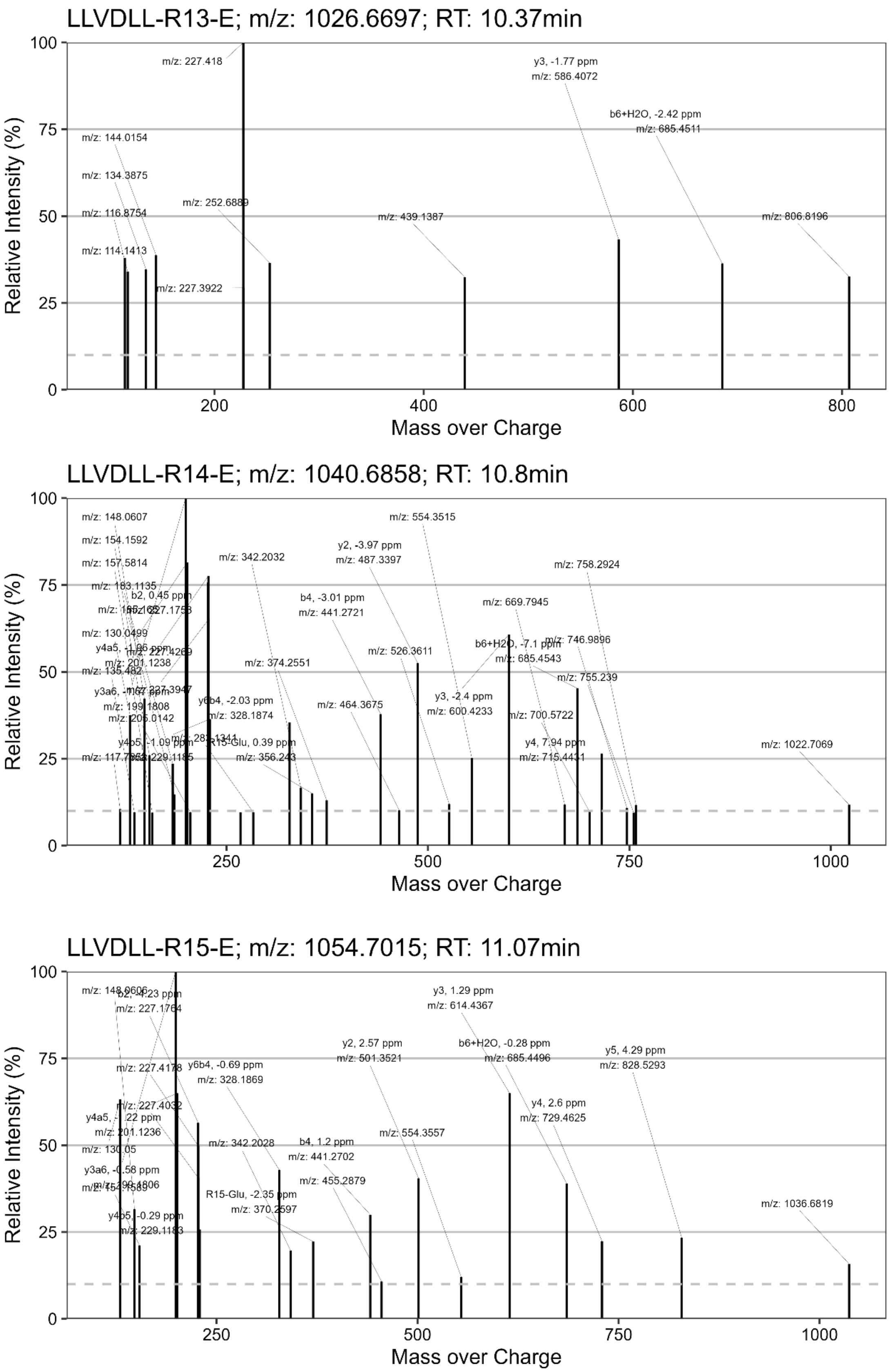

Figure SI 16: MS/MS Spectra of transformation products with fragments annotated in Roepstorff-Fohlmann-Biemann format. The precursor was removed from the spectra, and the remaining peaks were normalized to the most intense peak. Peaks smaller than 10% of the most intense peak were removed.

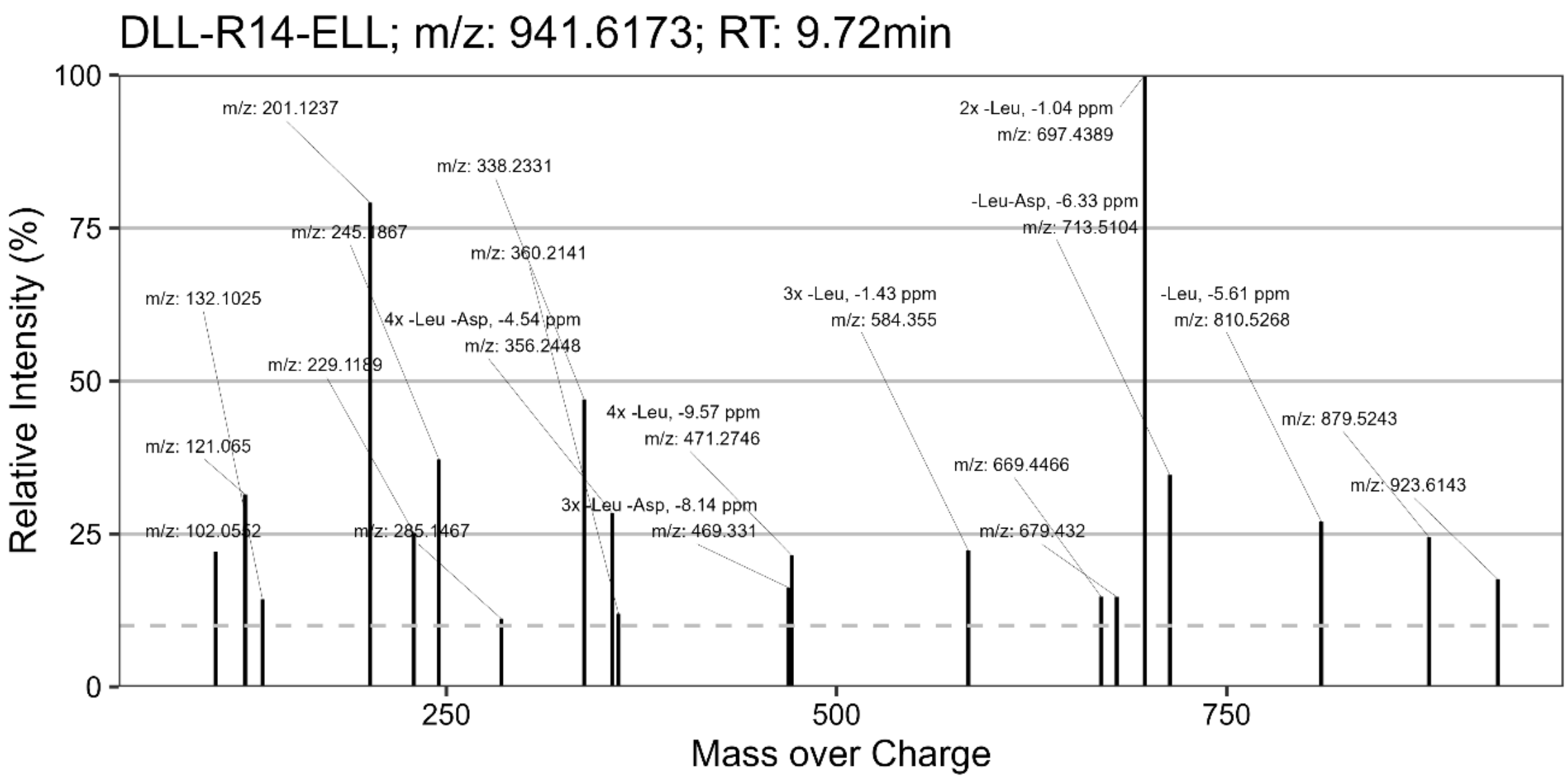

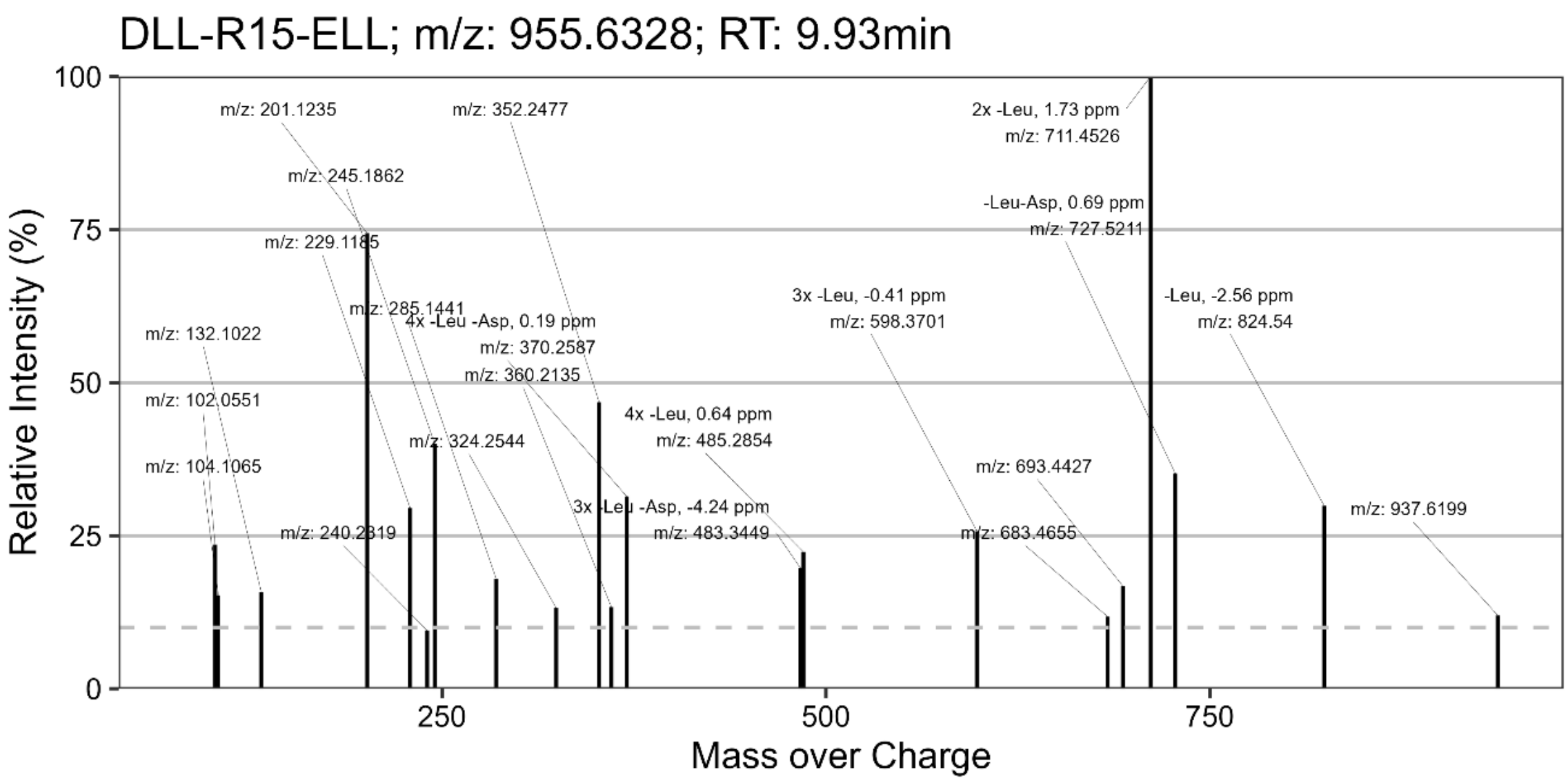

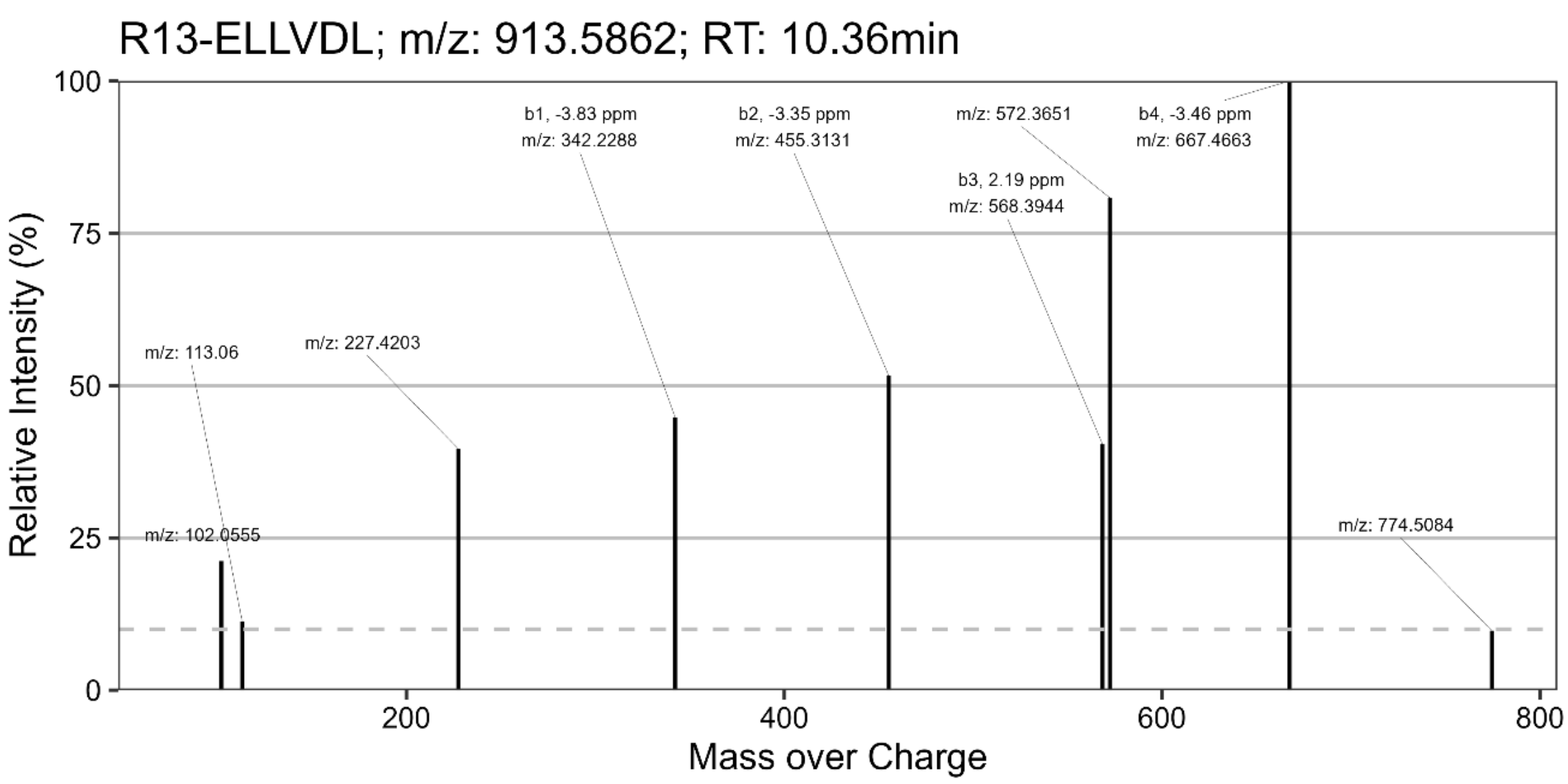

Figure SI 16 (continued)

R14A-ELLVDL; m/z: 927.6013; RT: 10.79min

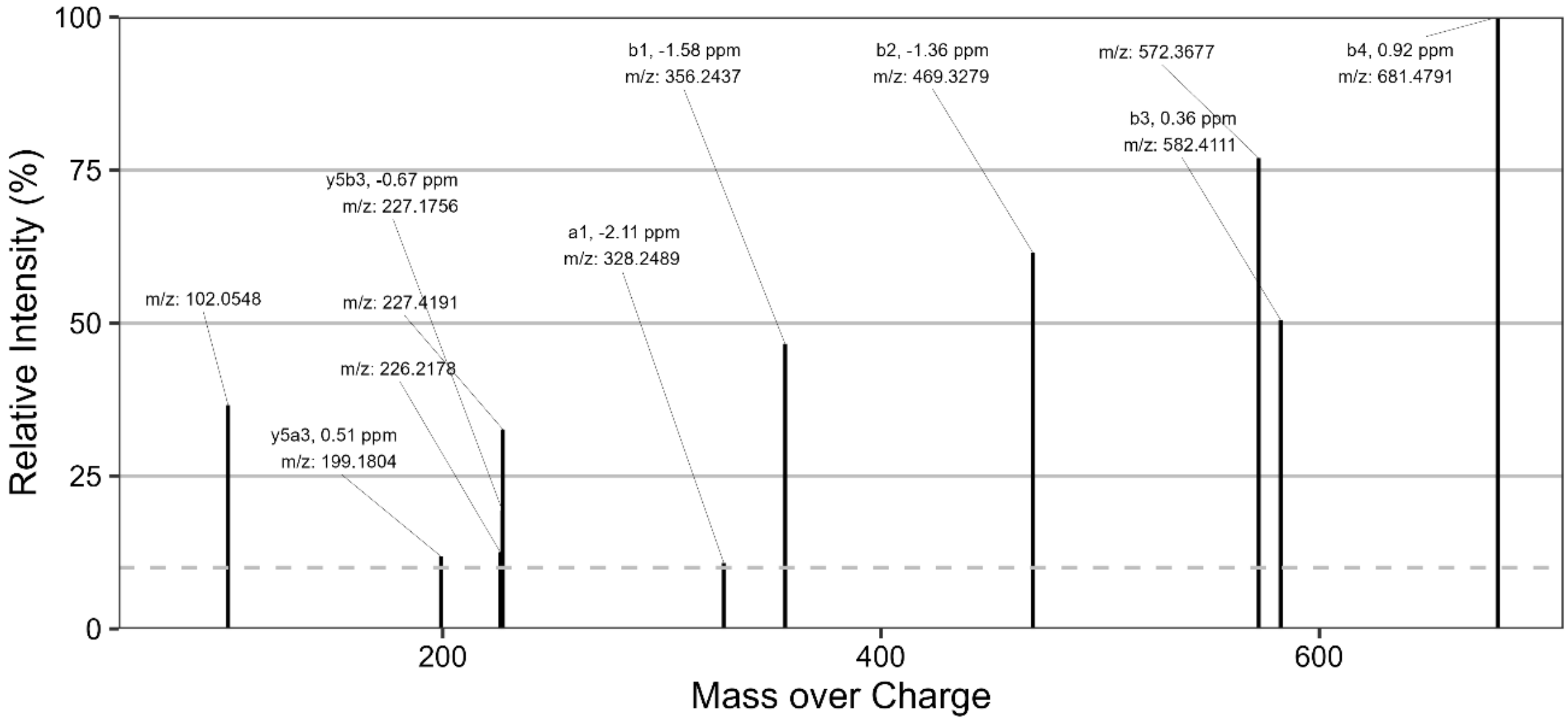

R14B-ELLVDL; m/z: 927.6013; RT: 10.74min

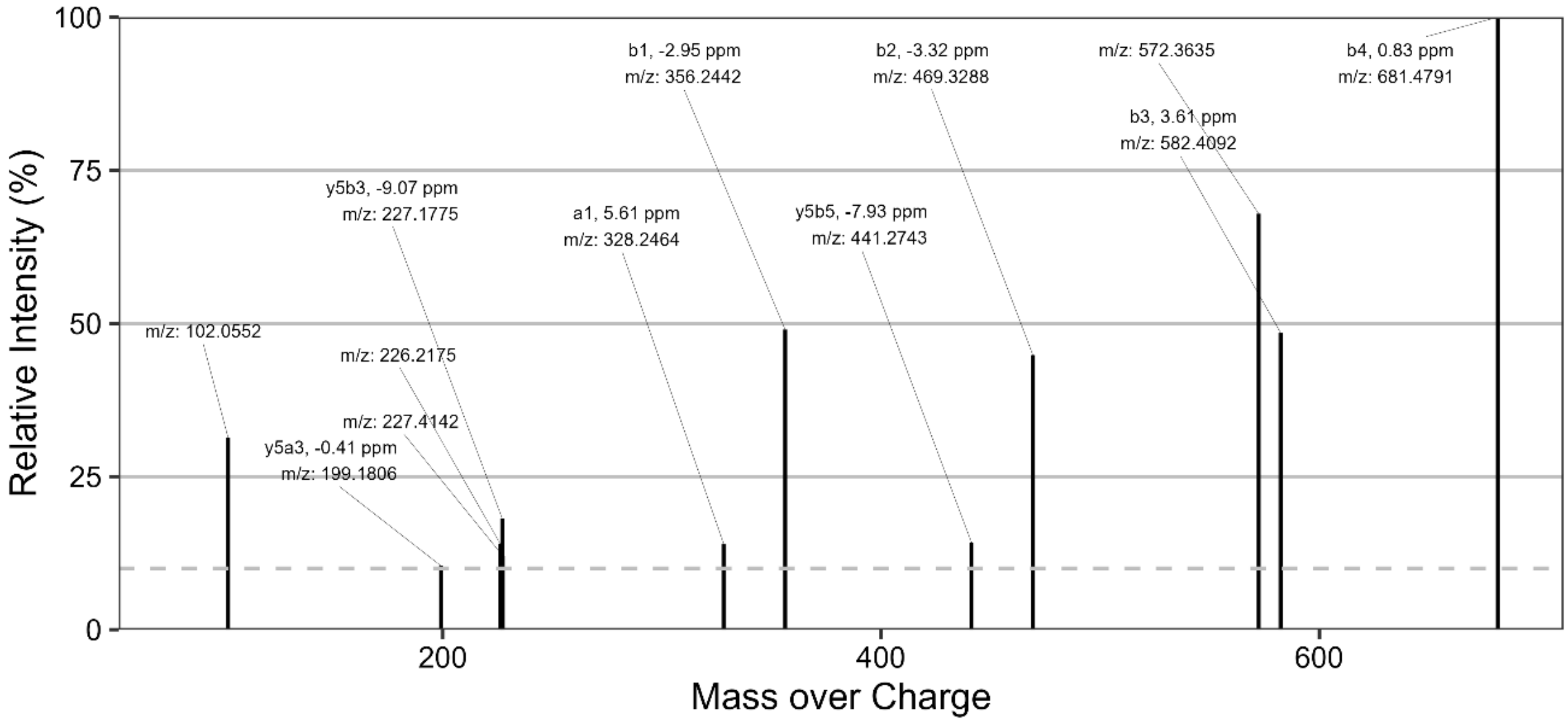

R15-ELLVDL; m/z: 941.6173; RT: 11.06min

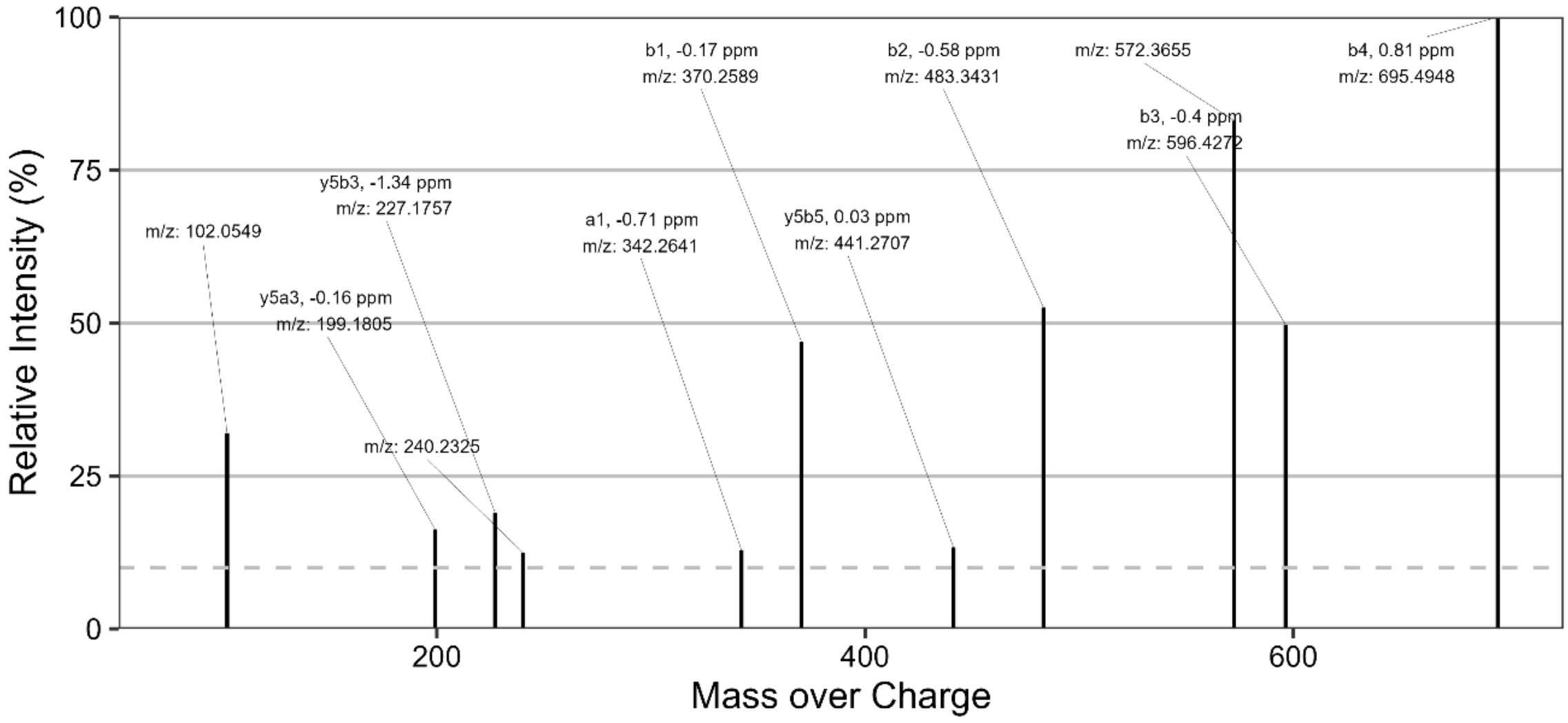

Figure SI 16 (continued)

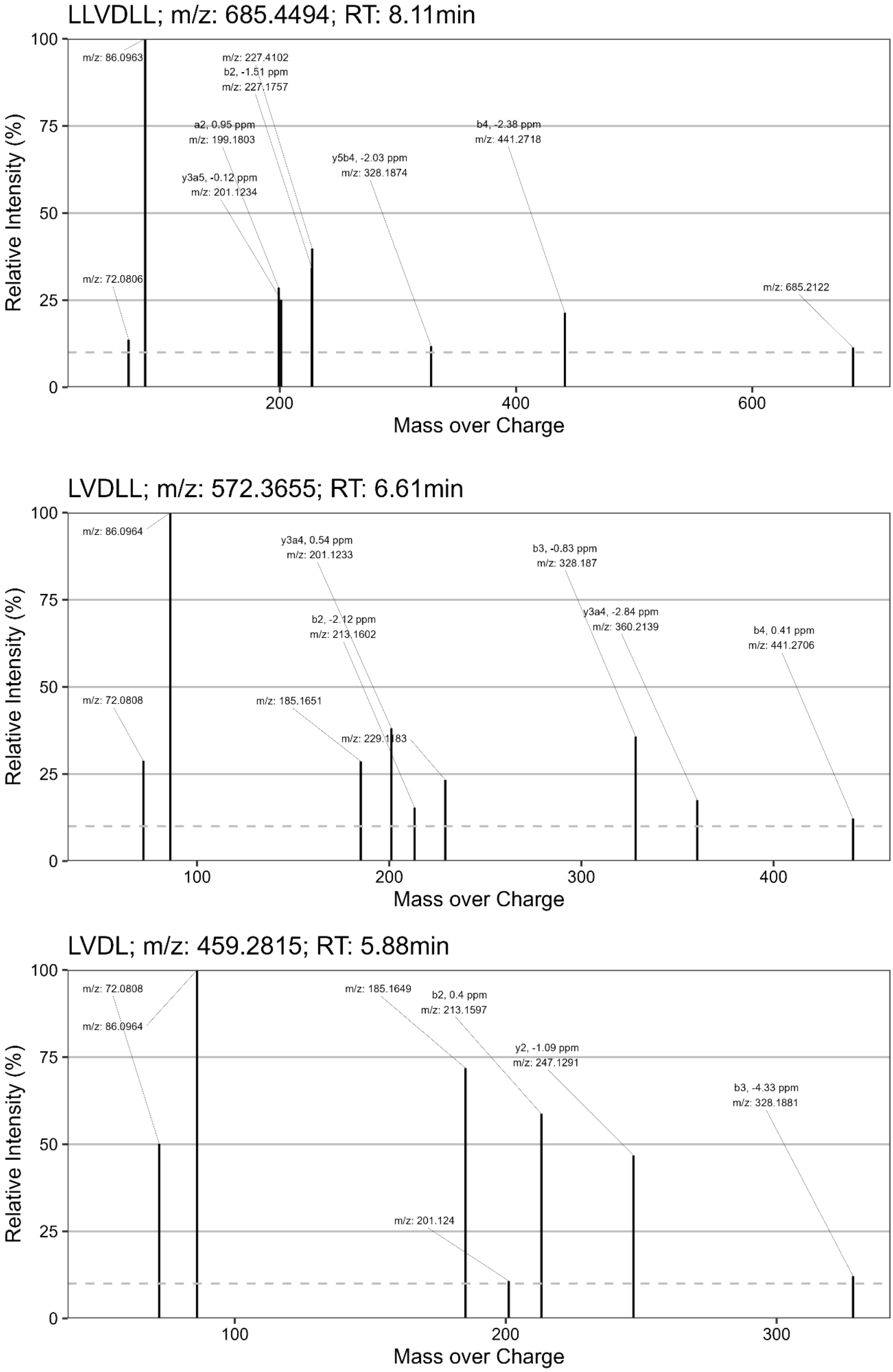

Figure SI 16 (continued)

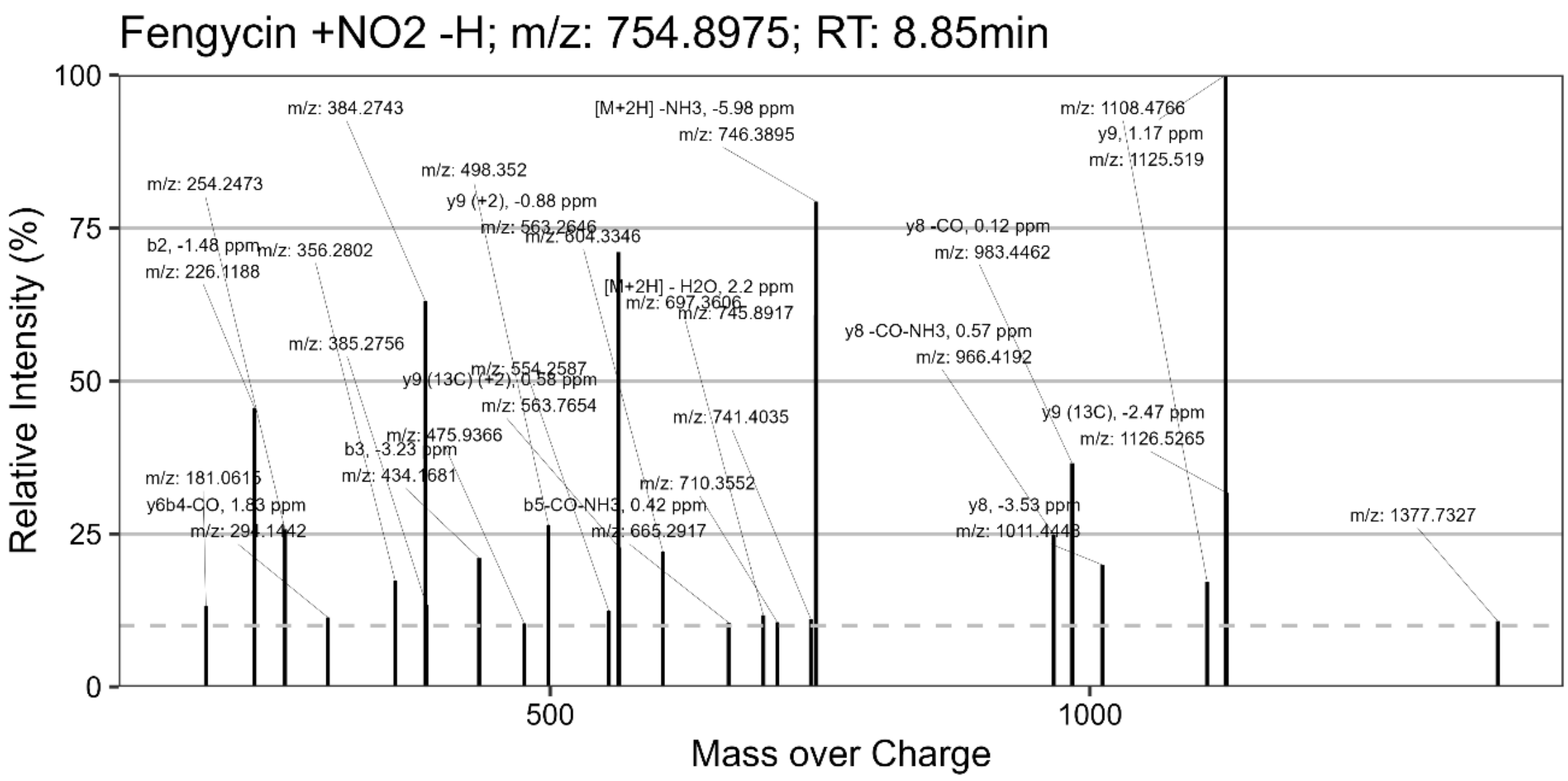

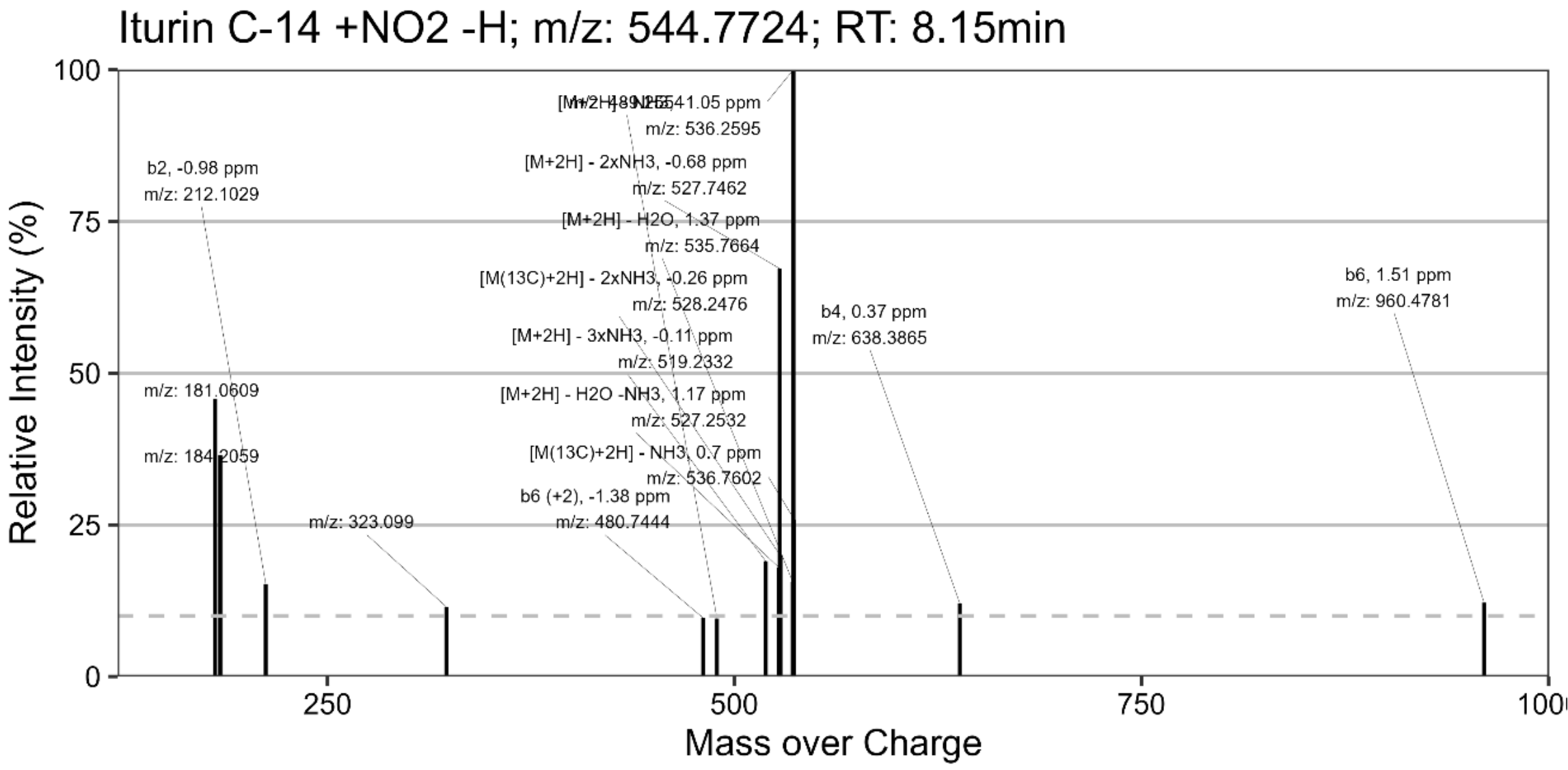

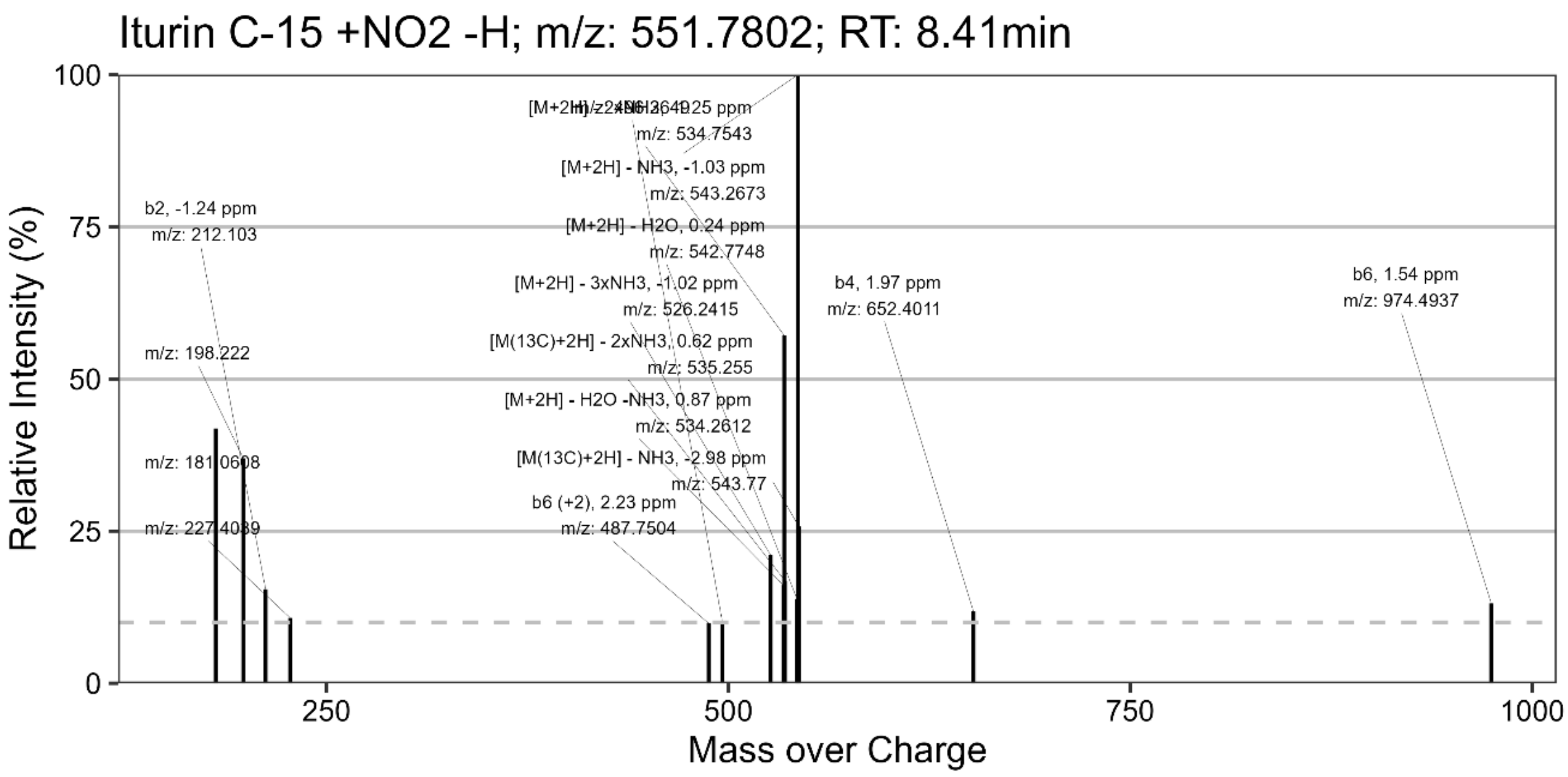

Figure SI 16 (continued)

Fengycin +O -2H; m/z: 739.394; RT: 7.4min

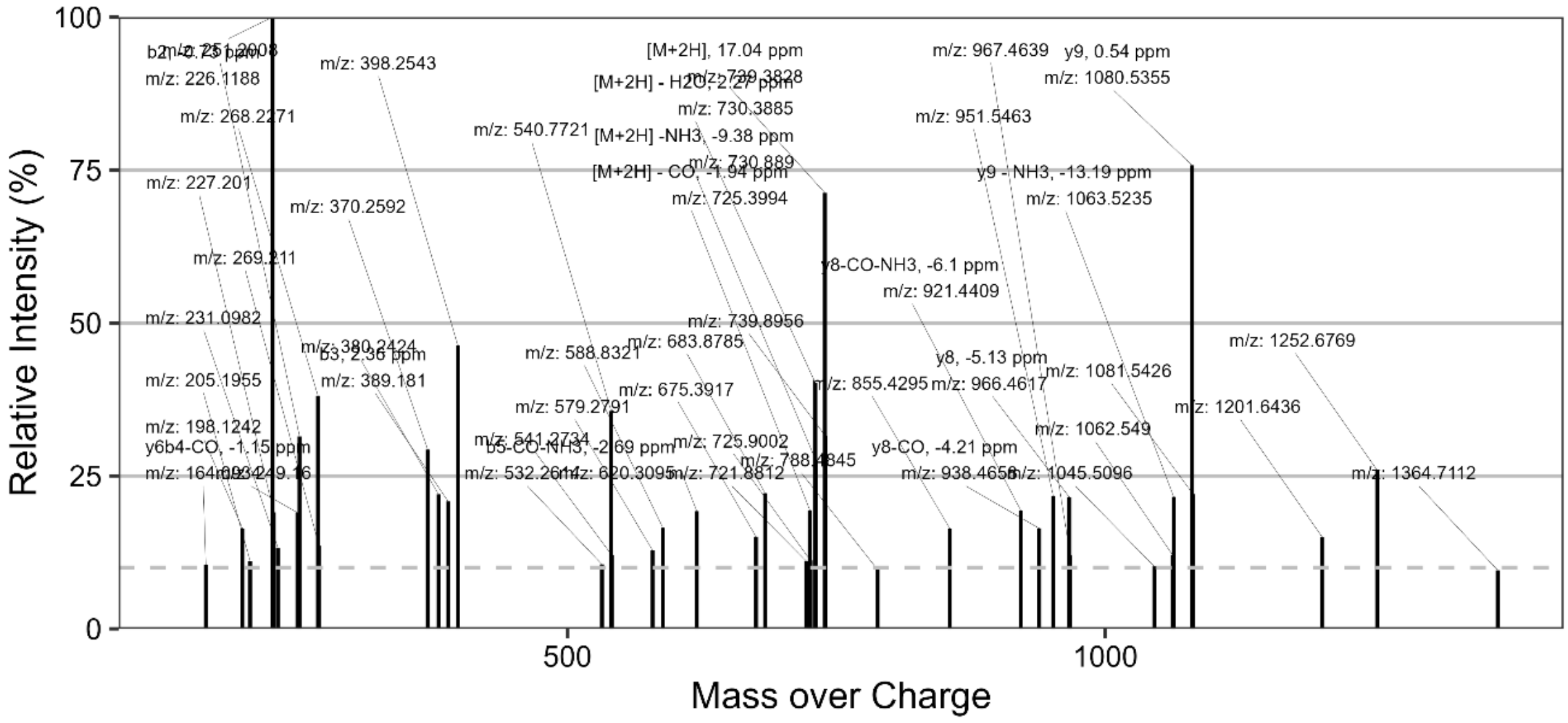

Iturin C-14 +O -2H; m/z: 529.269; RT: 7.65min

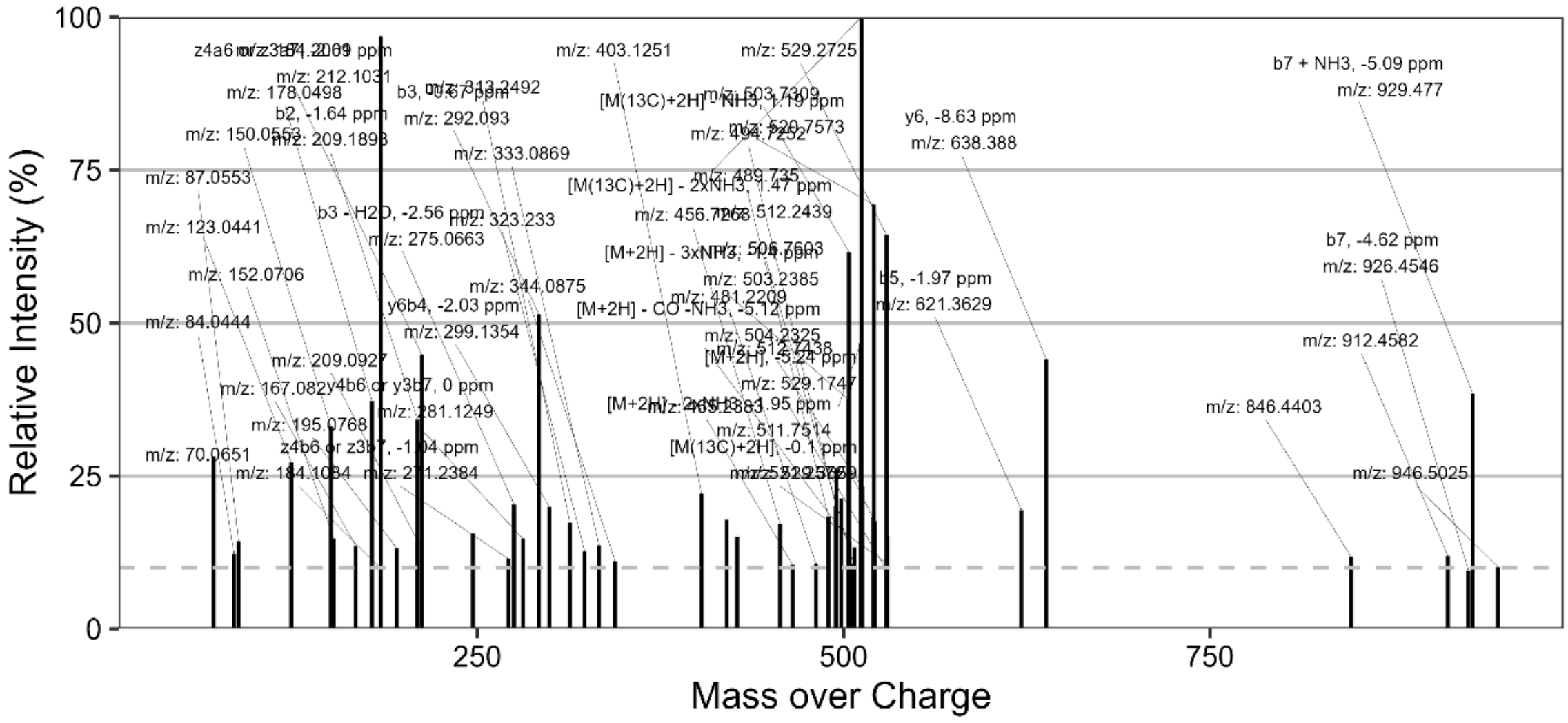

Iturin C-15 +O -2H; m/z: 536.277; RT: 7.95min

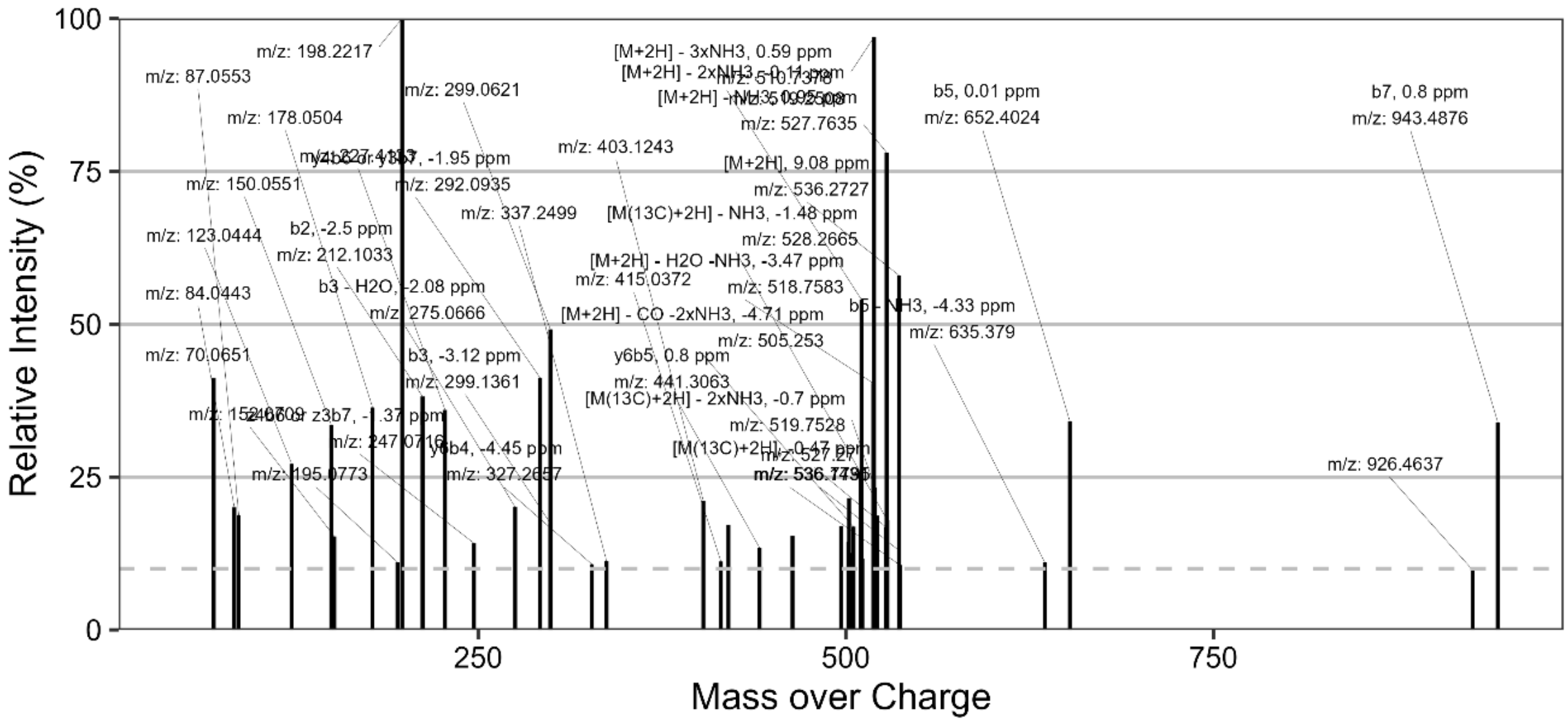

Figure SI 16 (continued)

Table SI 1: Soil parameters and classification adapted from "Landwirtschafltiche Untersuchungs- und Forschungsanstalt Speyer".

Soil Parameter	Soil 2.2	Soil 5M	Soil 6S
pH value (0.01 M CaCl2)	5.60 ± 0.29	7.45 ± 0.07	7.31 ± 0.06
Organic carbon (% C)	1.82 ± 0.48	1.18 ± 0.25	1.66 ± 0.20
Cation exchange capacity (meq/100g)	9.54 ± 1.36	9.86 ± 0.78	18.67 ± 1.65
Maximum water holding capacity (g/100g)	48.9 ± 5.6	41.5 ± 3.9	47.1 ± 7.1
Particle size distribution (mm)			
<0.002	9.7 ± 1.2	13.4 ± 0.6	41.2 ± 2.3
0.002 - 0.05	16.3 ± 1.5	33.1 ± 2.4	35.9 ± 1.3
0.05 - 2.0	74.0 ± 2.2	53.4 ± 2.2	22.9 ± 1.8
Soil Type (USDA)	sandy loam	sandy loam	clay

Table SI 2: Calculated lag phases (days) defined by different threshold values (i.e., 85%, 90%, and 95% of the concentration at day 0). For the final model, a lag phase with a threshold of 90% was selected.

	Soil 2.2			Soil 5M			Soil 6S		
**Name**	**85%**	**90%**	**95%**	**85%**	**90%**	**95%**	**85%**	**90%**	**95%**
Cyclic Surfactin C-12	14	7	2	0	0	0	1	0	0
Cyclic Surfactin C-13	14	14	14	2	2	0	2	2	1
Cyclic Surfactin C-14 7-Val	14	14	2	4	2	2	4	4	4
Cyclic Surfactin C-14 A	14	14	14	4	4	2	4	2	0
Cyclic Surfactin C-14 B	14	14	14	4	4	2	4	2	2
Cyclic Surfactin C-15 7-Val	14	14	14	7	7	4	14	14	14
Cyclic Surfactin C-15	14	14	14	7	4	4	7	7	4
Iturin C-14	0	0	0	1	0	0	4	4	0
Iturin C-15	0	0	0	0	0	0	0	0	0
Iturin C-16 A	0	0	0	0	0	0	1	1	0
Iturin C-16 B	0	0	0	0	0	0	2	2	0
Fengycin	0	0	0	0	0	0	1	1	0
Linear Surfactin C-12	1	0	0	0	0	0	0	0	0
Linear Surfactin C-13	2	1	0	0	0	0	0	0	0
Linear Surfactin C-14 A	2	2	0	0	0	0	1	0	0
Linear Surfactin C-14 B	2	2	2	1	0	0	1	0	0
Linear Surfactin C-15	2	2	0	1	1	0	1	1	1

Table SI 3: Summary of kinetic fits for lipopeptide concentrations in soil incubation experiments. Reported are the fitted starting concentration (C_0), first-order rate constant (k), half-life median, lower and upper values (68% confidence interval), relative mean square error (RMSE), overall recovery and start and end times of the model and the picked model type (i.e., 1-pool or 2-pool). The reported compartments represent operational parameters from kinetic model fitting and do not directly correspond to physically distinct sorbed or bioavailable fractions.

Name	Soil	Bioavailable Pool		Sorbed Pool		Half Life			RMSE (%)	Recovery (%)	Model		
		Concentration (nM)	Rate Constant (10^{-3} / d)	Concentration (nM)	Rate Constant (10^{-3} / d)	Lower (d)	Median (d)	Upper (d)			Start (d)	End (d)	Pools
Cyclic Surfactin C-12	2.2	320 ± 12	28 ± 3			23.0	25.2	27.7	5.7%	67 ± 4	7	102	1
Cyclic Surfactin C-13	2.2	341 ± 15	33 ± 3			18.9	20.7	22.9	5.9%	72 ± 1	14	102	1
Cyclic Surfactin C-14 7-Val	2.2	350 ± 13	30 ± 3			21.1	22.9	25.0	5.0%	79 ± 2	14	102	1
Cyclic Surfactin C-14 A	2.2	369 ± 15	31 ± 3			20.8	22.7	25.0	6.2%	78 ± 6	14	102	1
Cyclic Surfactin C-14 B	2.2	368 ± 15	32 ± 3			20.0	21.7	23.8	5.7%	77 ± 4	14	102	1
Cyclic Surfactin C-15 7-Val	2.2	360 ± 15	27 ± 3			23.9	26.1	28.9	5.6%	77 ± 6	14	102	1
Cyclic Surfactin C-15	2.2	367 ± 12	27 ± 2			23.8	25.6	27.7	4.9%	79 ± 2	14	102	1
Iturin C-14	2.2	422 ± 27	444 ± 65			1.4	1.6	1.8	8.0%	86 ± 3	0	4	1
Iturin C-15	2.2	397 ± 20	255 ± 30			2.4	2.7	3.1	7.6%	82 ± 4	0	10	1
Iturin C-16 A	2.2	359 ± 22	270 ± 38			2.3	2.6	3.0	7.7%	76 ± 3	0	10	1
Iturin C-16 B	2.2	362 ± 20	204 ± 26			3.0	3.4	3.9	7.9%	76 ± 4	0	14	1
Fengycin	2.2	176 ± 42	153 ± 66	73 ± 45	25 ± 17	7.0	7.9	9.5	4.5%	53 ± 3	0	70	2
Linear Surfactin C-12	2.2	183 ± 20	376 ± 89	111 ± 20	35 ± 9	3.6	4.0	4.4	3.5%	56 ± 4	0	55	2
Linear Surfactin C-13	2.2	84 ± 18	686 ± 364	171 ± 16	32 ± 4	9.7	11.1	12.6	3.8%	54 ± 4	1	70	2
Linear Surfactin C-14 A	2.2	66 ± 19	820 ± 591	190 ± 16	31 ± 4	13.1	14.4	16.1	4.1%	54 ± 3	1	102	2
Linear Surfactin C-14 B	2.2	221 ± 9	34 ± 4			18.6	20.5	22.9	5.1%	55 ± 6	2	102	1
Linear Surfactin C-15	2.2	227 ± 8	30 ± 3			21.5	23.4	25.8	4.5%	55 ± 4	2	102	1

Table SI 3 (continued):

Name	Soil	Bioavailable Pool		Sorbed Pool		Half Life			RMSE (%)	Recovery (%)	Model		
		Concentration (nM)	Rate Constant (10^-3 / d)	Concentration (nM)	Rate Constant (10^-3 / d)	Lower (d)	Median (d)	Upper (d)			Start (d)	End (d)	Pools
Cyclic Surfactin C-12	6S	200 ± 21	309 ± 83	144 ± 17	11 ± 3	5.3	6.1	7.3	5.9%	68 ± 8	0	102	2
Cyclic Surfactin C-13	6S	221 ± 34	168 ± 59	167 ± 31	8 ± 4	10.0	12.0	14.8	7.5%	84 ± 11	2	102	2
Cyclic Surfactin C-14 7-Val	6S	201 ± 51	77 ± 38	141 ± 54	5 ± 6	20.6	24.4	29.8	6.7%	72 ± 11	4	102	2
Cyclic Surfactin C-14 A	6S	223 ± 53	72 ± 32	140 ± 56	5 ± 6	19.9	22.8	26.8	6.5%	78 ± 12	2	102	2
Cyclic Surfactin C-14 B	6S	228 ± 50	67 ± 28	151 ± 53	3 ± 5	23.4	26.9	31.5	6.6%	77 ± 10	2	102	2
Cyclic Surfactin C-15 7-Val	6S	129 ± 52	86 ± 67	227 ± 54	5 ± 3	57.2	67.0	88.7	6.5%	76 ± 8	7	102	2
Cyclic Surfactin C-15	6S	167 ± 42	135 ± 76	215 ± 38	4 ± 3	31.8	42.6	55.8	7.0%	79 ± 10	14	102	2
Iturin C-14	6S	365 ± 11	182 ± 12			3.6	3.8	4.1	4.5%	74 ± 13	0	20	1
Iturin C-15	6S	316 ± 7	120 ± 6			5.5	5.8	6.1	2.7%	71 ± 13	1	40	1
Iturin C-16 A	6S	314 ± 8	90 ± 5			7.3	7.7	8.1	2.8%	70 ± 9	2	55	1
Iturin C-16 B	6S	338 ± 10	93 ± 6			7.0	7.4	7.9	3.9%	73 ± 10	1	55	1
Fengycin	6S	249 ± 5	45 ± 2			14.5	15.2	16.0	2.2%	55 ± 7	4	70	1
Linear Surfactin C-12	6S	200 ± 22	1336 ± 421	119 ± 17	50 ± 13	1.0	1.2	1.5	4.6%	64 ± 2	0	40	2
Linear Surfactin C-13	6S	215 ± 29	789 ± 236	127 ± 26	56 ± 17	1.7	1.9	2.3	5.0%	68 ± 6	0	40	2
Linear Surfactin C-14 A	6S	128 ± 45	538 ± 356	148 ± 45	59 ± 21	4.1	5.2	6.7	5.3%	54 ± 4	0	40	2
Linear Surfactin C-14 B	6S	142 ± 41	686 ± 411	178 ± 39	57 ± 15	3.9	4.9	6.2	6.1%	63 ± 3	0	40	2
Linear Surfactin C-15	6S	272 ± 16	82 ± 11			7.5	8.5	9.8	7.0%	61 ± 4	1	40	1

Table SI 3 (continued):

Name	Soil	Bioavailable Pool		Sorbed Pool		Half Life			RMSE (%)	Recovery (%)	Model		
		Concentration (nM)	Rate Constant (10^-3 / d)	Concentration (nM)	Rate Constant (10^-3 / d)	Lower (d)	Median (d)	Upper (d)			Start (d)	End (d)	Pools
Cyclic Surfactin C-12	5M	201 ± 77	146 ± 102	160 ± 82	20 ± 12	11.0	13.5	17.8	8.5%	75 ± 5	0	70	2
Cyclic Surfactin C-13	5M	230 ± 114	138 ± 120	200 ± 120	18 ± 13	12.4	15.8	20.9	11.1%	94 ± 1	2	70	2
Cyclic Surfactin C-14 7-Val	5M	215 ± 69	162 ± 101	196 ± 72	19 ± 8	11.1	13.8	18.2	9.1%	80 ± 2	2	102	2
Cyclic Surfactin C-14 A	5M	210 ± 83	183 ± 139	205 ± 84	18 ± 10	10.8	14.2	19.1	10.6%	90 ± 3	4	70	2
Cyclic Surfactin C-14 B	5M	144 ± 91	187 ± 217	254 ± 94	20 ± 7	18.1	22.4	28.5	10.3%	85 ± 1	4	102	2
Cyclic Surfactin C-15 7-Val	5M	157 ± 72	203 ± 182	265 ± 72	18 ± 6	17.4	21.5	27.0	9.8%	85 ± 6	4	102	2
Cyclic Surfactin C-15	5M	103 ± 70	285 ± 380	309 ± 70	21 ± 5	21.7	25.0	29.5	8.3%	90 ± 2	7	102	2
Iturin C-14	5M	456 ± 11	556 ± 31			1.2	1.2	1.3	3.5%	90 ± 3	0	4	1
Iturin C-15	5M	463 ± 10	375 ± 18			1.8	1.8	1.9	3.2%	92 ± 1	0	10	1
Iturin C-16 A	5M	108 ± 261	366 ± 122	306 ± 254	1134 ± 1957	2.3	2.8	3.6	5.3%	83 ± 6	0	10	2
Iturin C-16 B	5M	133 ± 203	392 ± 102	312 ± 198	1175 ± 1595	2.3	2.6	3.1	4.2%	89 ± 2	0	10	2
Fengycin	5M	365 ± 6	139 ± 5			4.8	5.0	5.2	2.7%	73 ± 1	0	20	1
Linear Surfactin C-12	5M	199 ± 45	887 ± 375	125 ± 45	112 ± 43	1.5	1.8	2.2	4.7%	64 ± 2	0	20	2
Linear Surfactin C-13	5M	174 ± 51	875 ± 490	169 ± 50	96 ± 30	2.2	2.7	3.5	5.9%	68 ± 4	0	28	2
Linear Surfactin C-14 A	5M	116 ± 64	937 ± 933	209 ± 63	98 ± 26	3.7	4.5	5.7	6.4%	64 ± 2	0	28	2
Linear Surfactin C-14 B	5M	130 ± 63	732 ± 643	194 ± 64	84 ± 25	3.8	4.8	6.1	6.8%	64 ± 4	0	40	2
Linear Surfactin C-15	5M	227 ± 86	161 ± 103	67 ± 90	29 ± 45	5.9	6.9	8.8	7.1%	67 ± 4	1	55	2

Table SI 4: Features, detected by non-target screening, for which no tentative structure could be proposed. Mass-to-charge ratio (m/z), retention time (RT in minutes), and highest peak intensity (a.u.) are reported.

Transformation	2.2			5M			6S		
Product	m/z	RT	Intensity	m/z	RT	Intensity	m/z	RT	Intensity
TP1	175.1481	9.35	1.60E+07	518.3242	9.42	1.00E+07	434.1348	9.33	4.00E+07
TP2	518.3242	9.40	4.50E+06	175.1481	11.22	9.00E+06	340.2496	8.83	4.00E+07
TP3	264.1418	11.53	1.40E+06	476.2769	9.39	3.00E+06	387.1931	9.12	3.00E+07
TP4	894.5420	12.82	6.80E+05	778.5378	13.62	2.00E+06	377.1132	9.25	2.00E+07
TP5	294.1523	10.69	6.60E+05	413.2257	9.36	2.00E+06	477.3475	12.07	1.00E+07
TP6	476.1174	9.90	6.00E+05	1078.7141	13.68	1.00E+06	593.2760	12.43	8.00E+06
TP7	512.3136	10.09	5.00E+05	419.1919	12.31	6.00E+05	354.2653	10.70	8.00E+06
TP8	478.1142	9.80	4.00E+05	287.6256	9.42	5.00E+05	493.3425	12.56	6.00E+06
TP9	248.1645	10.19	4.00E+05	944.7161	11.94	4.00E+05	452.2797	7.61	4.00E+06
TP10	522.9079	8.82	4.00E+05	959.7341	12.15	3.80E+05	265.1799	10.15	3.00E+06
TP11	703.1937	9.77	4.50E+05	477.3477	12.08	3.80E+05	175.1481	9.35	3.00E+06
TP12	417.1672	7.45	4.00E+05	963.5991	11.07	3.50E+05	436.1504	7.90	2.00E+06
TP13	248.1645	10.19	4.00E+05	294.1523	10.70	3.30E+05	505.3790	12.66	2.00E+06
TP14	478.1142	9.80	4.00E+05	282.1853	10.81	3.30E+05	476.2367	11.24	2.00E+06
TP15	522.5745	8.82	3.70E+05	1064.6982	13.37	3.20E+05	518.2668	11.54	1.60E+06
TP16	448.2454	7.45	3.70E+05	945.7189	11.94	3.10E+05	484.3035	9.36	1.50E+06
TP17	522.9079	8.82	3.60E+05	622.4471	13.28	3.10E+05	317.2687	10.30	1.40E+06
TP18	340.1908	11.18	3.40E+05	288.1245	9.42	3.10E+05	698.4320	10.51	1.30E+06
TP19	522.2411	8.82	3.00E+05	800.5240	13.62	2.40E+05	501.3037	10.30	1.20E+06
TP20	913.6377	10.76	3.00E+05	1101.7901	13.68	2.20E+05	490.7980	6.55	1.20E+06
TP21	150.0775	10.70	2.50E+05	548.3712	10.53	2.20E+05	506.2474	10.98	7.00E+05
TP22	225.1023	7.63	2.40E+05	476.2334	10.63	2.20E+05	380.3160	7.69	7.00E+05
TP23	402.1912	9.66	2.30E+05	955.6327	9.93	2.00E+05	583.3608	11.27	6.00E+05
TP24	783.3577	8.82	2.30E+05	399.7713	9.86	2.00E+05	479.3267	12.31	6.00E+05
TP25	783.8581	8.82	2.30E+05	307.1328	9.65	2.00E+05	497.8059	6.67	5.00E+05
TP26	739.8962	7.40	2.30E+05				479.1930	9.32	5.00E+05
TP27	532.3398	10.66	2.20E+05				136.0618	8.83	5.00E+05
TP28	523.2415	8.82	2.20E+05				420.1555	7.52	5.00E+05
TP29	432.3237	10.81	2.20E+05				684.4163	10.00	5.00E+05
TP30	570.3559	10.28	2.10E+05				289.4780	11.34	4.60E+05
TP31	818.4370	9.12	2.10E+05				339.1227	8.34	4.50E+05
TP32	452.3008	10.62	2.10E+05				524.3796	10.12	4.00E+05
TP33	1059.7713	11.16	2.00E+05				1036.6539	10.50	4.00E+05
TP34	873.6453	10.76	2.00E+05				529.2694	6.46	4.00E+05
TP35							710.4837	12.18	4.00E+05
TP36							479.3217	10.29	3.50E+05
TP37							361.1183	10.12	3.40E+05
TP38							1163.7412	12.15	3.00E+05
TP39							318.1560	6.75	2.60E+05
TP40							789.5124	12.44	2.50E+05
TP41							379.1288	7.53	2.10E+05
